# Naringenin attenuates slow-transit constipation by regulating the AMPK/mTOR/ULK1 signalling pathway: *in vivo* and *in vitro* studies

**DOI:** 10.3389/fphar.2025.1550458

**Published:** 2025-06-17

**Authors:** Yahui Wang, Xiaopeng Wang, Yifei Qian, Mingming Sun, Huiju Yang, Lianlin Su, Shuai Yan

**Affiliations:** ^1^ Department of Anorectal Surgery, Suzhou TCM Hospital Affiliated to Nanjing University of Chinese Medicine, Suzhou, Jiangsu, China; ^2^ Department of Chemistry, University of Wisconsin—Madison, Madison, WI, United States; ^3^ Department of Anorectal Surgery, The Third Affiliated Hospital of Henan University of Chinese Medicine, Zhengzhou, Henan, China; ^4^ School of Pharmacy, Nanjing University of Chinese Medicine, Nanjing, Jiangsu, China

**Keywords:** naringenin, autophagy, AMPK/mTOR/ULK1 signalling pathway, interstitial cells of Cajal, slow transit constipation

## Abstract

**Background:**

Slow-transit constipation (STC) is a widespread functional gastrointestinal condition distinguished by decreased colonic motility as an essential clinical characteristic. The excessive autophagy of interstitial cells of Cajal (ICCs) causes phenotypic changes and functional abnormalities, which are important in colonic dysmotility. Naringenin (NAR) has been shown to regulate gastrointestinal motility disorders. The present study aimed to elucidate the regulatory role of naringenin in autophagy in STC and its underlying mechanism.

**Methods:**

*In vitro*, ICCs were stimulated with L-glutamic acid (GA) to induce autophagy and treated with NAR. A CCK8 assay was performed to evaluate the cytotoxic effect of NAR. Annexin V-FITC/PI staining was used to examine NAR apoptosis. The expression of the autophagy markers Beclin1 and LC3B, as well as proteins related to the AMPK/mTOR/ULK1 pathway was investigated through quantitative PCR, Western blot analysis and immunofluorescence staining. The small interfering RNA (siRNA) technique was used to knockdown selective autophagy receptors (NDP52, OPTN, NBR1, and p62) in ICCs. Coimmunoprecipitation (co-IP) was used to evaluate the binding of pS757-ULK1 to the autophagy receptors NDP52 and OPTN in ICCs. Immunofluorescence (IF) staining was performed to observe the colocalization of pS757-ULK1 with exogenous NDP52 and OPTN in ICCs. *In vivo*, male C57BL/6 mice were administered loperamide (10 mg/kg) to establish a constipation model and then treated with NAR (75/150/300 mg/kg) for 2 weeks. Finally, colonic tissues were collected for a histological analysis and immunohistochemical for cell growth factor receptor kit (c-Kit) and anoctamin-1 (ANO1).

**Results:**

Our results indicated that NAR improved the survival and apoptosis of ICCs after GA by inhibiting autophagy through the partial suppression of the AMPK/mTOR/ULK1 signalling pathway. Moreover, NAR inhibited the autophagic degradation of pS757-ULK1 by weakening the interactions between pS757-ULK1 and the selective autophagy receptor genes NDP52 and OPTN. Further research revealed that NAR could increase the moisture content of faeces; increase the rate of small intestinal propulsion in mice; increase the serum concentrations of excitatory neurotransmitters such as GAS, 5-HT, MTL, and SP; and increase the expression levels of ANO1 and c-Kit in the colon, and the molecular mechanism was consistent with the *in vitro* results.

**Conclusion:**

NAR attenuates the AMPK/mTOR/ULK1 pathway in ICCs, thereby improving STC colonic dysmotility and underscoring its promise as a therapeutic option for STC.

## Graphical Abstract

**Figure:**
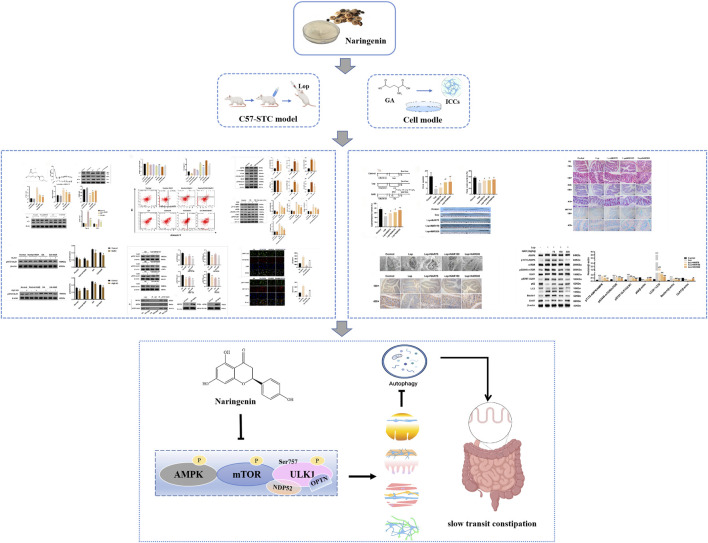
Naringenin attenuates slow-transit constipation by regulating the AMPK/mTOR/ULK1 signalling pathway, both *in vitro* and *in vivo*, improving colonic function.

## 1 Introduction

Slow transit constipation (STC) is among the most prevalent and challenging intractable digestive tract disorders and is characterized by weakened colonic transit function, delayed intestinal content discharge, and normal rectal output and pelvic floor performance. It is the most frequently occurring variant of functional constipation (Aziz et al., 2020). The increasing incidence of STC is attributable to an ageing population, evolving dietary patterns and lifestyles, and the overuse of stimulant laxatives (Vazquez Roque and Bouras, 2015). Epidemiological studies have shown that the global incidence of functional constipation, as defined by the Rome IV criteria, is 10.1%, among which the incidence of STC in FC patients in Western countries and Asia is 13%–37% (Barberio et al., 2021). Chronic constipation can lead to numerous health issues, including myocardial infarction, stroke, and colorectal cancer. Additionally, it is associated with various psychological problems, significantly contributing to a diminished quality of life and increased economic burden for STC patients (Ranasinghe et al., 2017). Despite ongoing advancements in treatment options in recent years, patient satisfaction with STC management remains low. Presently, no medication or intervention can fully prevent or cure STC (Aziz et al., 2020).

The main pathological feature of STC is colonic motility disorder. Interstitial cells of Cajal (ICCs) are the initiators and propagators of slow waves in gastrointestinal smooth muscle. They are regarded as the pacemaker cells of gastrointestinal motility and exert considerable influences on regulating the rhythm, amplitude and direction of gastrointestinal smooth muscle contraction (Foong et al., 2020). Colonic motility disorders are often closely associated with abnormalities in the ICC phenotype (morphology, distribution, number, and structure). Research by Zhu et al. has shown that the density of ICCs in the colons of STC patients is markedly lower than that in healthy individuals. This reduction leads to insufficient slow wave activity, adversely impacting the contractile response in STC patients and causing transmission disorders (Zhu et al., 2016). Postoperative pathology in clinical studies confirmed that in patients with STC, the size of the colonic ICCs was markedly decreased, accompanied by a reduction in cell processes, ganglion vacuolar degeneration, nuclear condensation, and ICC processes and disordered connections (Geramizadeh et al., 2009). In addition, the c-Kit ligand SCF has the same role as the c-Kit receptor in supporting the development, maturation and preservation of the normal phenotype of ICCs. Research has shown that after treatment with SCF for 1 week, the labelling of c-Kit (+) cells in the myenteric plexus increased markedly following exposure to the c-Kit antibody ACK2, and the amplitude and frequency of the electrical rhythm were significantly restored, indicating that the function of ICCs as pacemaker cells was restored (Kishi et al., 2020). Torihashi et al. injected ACK2 into mice for eight consecutive days after birth, performed immunohistochemistry and electron microscopy, and found after c-Kit receptor blockade, ICCs in the small intestine almost disappeared, and the morphology and structure of the remaining c-Kit-positive pacemaker cells were similar to those of smooth muscle cells (Shu et al., 2023). The aforementioned studies revealed that ICCs can undergo a smooth muscle cell phenotypic transformation and that transformed ICCs lose their normal morphology and function, causing gastrointestinal motility disorders.

Autophagy is a self-stress response in eukaryotic cells in which damaged and aged organelles are transferred from the cell to lysosomes, where these substrates are digested and degraded to produce energy for the body (Al-Bari, 2020; Zhao and Wang, 2021). Normal cellular autophagy is a physiological state through which cells can resist abnormal external metabolic pressure and nutrient deficiency to maintain the balance of the intracellular environment; however, excessive autophagy is a pathological state that produces an abundance of autophagosomes that exceed the degradation capacity of their own lysosomes, thereby destroying intracellular homeostasis, damaging the normal cell structure, and even causing autophagic cell death. The excessive autophagy and apoptosis of ICCs have been observed in individuals with gastrointestinal (GI) motility disorders, including STC. A recent study confirmed that the excessive autophagy of ICCs is a key factor contributing to gastrointestinal motility disorders (Zheng et al., 2021) and is closely associated with the occurrence of STC. The AMPK/mTOR/ULK1 signalling pathway stands as a cornerstone in cellular autophagy research and remains a prominent focus within the field. Autophagy activation in ICCs is intricately associated with the AMPK/mTOR/ULK1 signalling pathway. However, the regulatory effect of the AMPK/mTOR/ULK1 pathway on ICC autophagy and the consequent phenotypic alterations remain unknown.

Chinese herbal medicines have historically been utilized for millennia to treat intestinal disorders (China Association of Chinese Medicine, 2024; Liu et al., 2024). Some small-molecule compounds derived from these herbs are emerging as potential candidates for therapeutic interventions for STC. Naringenin (NAR), a bioactive flavanone derived from citrus fruits and tomatoes, has garnered significant attention in research owing to its diverse pharmacological properties (Salehi et al., 2019). Research has indicated that NAR is a helpful treatment for problems of the digestive system, including functional gastrointestinal disorders, alcoholic and nonalcoholic fatty liver diseases, colorectal cancer, and pancreatobiliary diseases (Arafah et al., 2020). The regulatory effects of NAR on intestinal motility disorders have been comprehensively observed. These effects are attributed primarily to its ability to promote Cl^−^ secretion in the colonic epithelium through the cAMP/PKA pathway and to increase the levels of markers (c-Kit and SCF), along with AQP3 (Yang et al., 2008; Yin et al., 2018). The regulation of autophagy by NAR, a citrus flavonoid, is predominantly associated with the AMPK/mTOR/ULK1 pathway (Foong et al., 2020; Wang et al., 2018), as evidenced by multiple studies. However, the current literature does not conclusively establish this pathway as the sole mechanism, as emerging evidence suggests the potential involvement of other pathways, although the PI3K/AKT pathway appears largely excluded. NAR activates AMPK, leading to mTOR inhibition. This process relieves the mTOR-mediated suppression of ULK1, initiating autophagy. Studies of hepatocytes (Yang et al., 2021) and non-small cell lung cancer cells (Chang et al., 2024) have shown that AMPK inhibition abolishes naringenin-induced autophagy, confirming its centrality. However, the mechanisms by which NAR modulates autophagy in individuals with constipation remain poorly understood. Our findings reveal how NAR inhibits STC by altering the expression of AMPK/mTOR/ULK1, paving the way for the application of a novel method for its prevention and therapy.

## 2 Materials and methods

### 2.1 Reagents

NAR (purity ≥99%, catalogue number: N1370), BafA1 (catalogue number: A8627), and dorsomorphin (catalogue number: B3252) were purchased from the APExBIO Technology Limited-liability Company (Houston, TX, United States). Loperamide (catalogue number: MDJ7007) was acquired from Xian Janssen Pharmaceutical Ltd. (Xi’an, China). Primary antibodies against LC3 (catalogue number: ab128025), Beclin1 (catalogue number: ab207612), pS757-ULK1 (catalogue number: ab229909) and c-Kit (catalogue number: ab256345) were acquired from Abcam (Cambridge, UK). Glutamate (catalogue number: V900408-100G) was acquired from Sigma‒Aldrich (St. Louis, MO, United States). Primary antibodies against P62 (catalogue number: 66184-1-Ig), AMPK (catalogue number: 66536-1-Ig), mTOR (catalogue number: 66888-1-Ig), pS2448-mTOR (catalogue number: 67778-1-Ig) and ChAT (catalogue number: 20747-1-AP) were acquired from Proteintech Group (Chicago, IL, United States). The antibodies directed against pT172-AMPK (catalogue number: 2,535) and ULK1 (catalogue number: 3602R) were acquired from Cell Signaling Technology (Danvers, MA, United States). Lipofectamine^™^ 2000 transfection reagent (catalogue number: 11668030) was acquired from Invitrogen (Carlsbad, CA, United States). Motilin (MTL), gastrin (GAS), substance P (SP), vasoactive intestinal peptide (VIP), somatostatin (SS), and 5-hydroxy tryptamine (5-HT) ELISA kits were acquired from Shanghai Enzyme Link Biotechnology Co. (Shanghai, China). Foetal bovine serum (FBS) (catalogue number: 10099141) and 0.25% trypsin were acquired from Gibco (NY, United States). A cell counting kit-8 (Lot number: NU679) was purchased from DOJINDO (Kamimashiki-gun, Kumamoto, Japan).

### 2.2 Cell culture and cell viability assays

Interstitial cells of Cajal (ICCs) were acquired from iCell Bioscience, Inc. (Shanghai, China). The ICCs were grown in mesenchymal cell complete culture medium (Lot number: PriMed-iCell-025, iCell Bioscience, Inc.) supplemented with 1% penicillin/streptomycin in a humidified incubator set at 37°C and containing 5% CO_2_ (see Supplementary Figure S1). Following a cultivation period of 4–8 days, the culture medium was removed and replaced with fresh complete culture medium for mesenchymal cells (containing 10% FBS). Half of the medium was subsequently replaced every 3 days. A range of concentrations of NAR (0 nM, 1 nM, 5 nM, 10 nM, 20 nM, 50 nM, 100 nM, 200 nM, 300 nM and 500 nM) was subsequently administered for 24 h. Cell viability was assessed using the CCK8 assay according to the manufacturer’s instructions. Cell viability was calculated as follows: Cell viability (%) = Cell survival rate = [(OD_a_ - OD_b_)/(OD_c_ - OD_b_)] × 100%. OD_a_ represents the absorbance of the experimental well, OD_b_ represents the absorbance of the blank well, and ODc denotes the absorbance of the control well.

### 2.3 Flow cytometry

For the flow cytometry analysis, the GA (5 mM) solution was prepared by dissolving GA in DMSO, and ICCs were incubated with GA for 24 h to construct the ICC model of autophagy. The ICCs from various groups were plated into 6-well plates. After treatment, the ICCs were collected by an incubation with trypsin without EDTA, washed twice with phosphate-buffered saline (PBS), and centrifuged at 2000 rpm for 5 min to collect approximately 3.2 × 10^5^ cells. Then, 500 μL of binding buffer was added to resuspend the cells. In accordance with the manufacturer’s instructions, the fixed cells were labelled using an Annexin V-APC Apoptosis Detection Kit (Keygen Biotechnology, KGA1030) by adding 5 μL of Annexin V-APC and mixing and then adding 5 μL of propidium iodide (PI) and mixing. The mixture was incubated at room temperature in the dark for 10 minutes. The samples were then subjected to an analysis with a BD FACSCalibur flow cytometer (BD Biosciences, San Diego, CA, United States), which collected data from at least 10,000 cells per sample. FlowJo software was used to analyse the data. The proportion of apoptotic cells was assessed by gating on the Annexin V-positive and PI-negative populations (early apoptotic cells), as well as the Annexin V-positive and PI-positive populations (late apoptotic/necrotic cells).

### 2.4 Western blot analysis

All the samples were harvested and lysed in RIPA lysis buffer. Protein concentrations were determined using a BCA protein quantification kit (Abiowell, Changsha, China), and equal quantities of protein from each sample were loaded into the lanes. These proteins were then separated via SDS‒PAGE and transferred onto a PVDF membrane. The membrane was blocked with 5% nonfat dry milk at room temperature for 90 min and then incubated at 4°C overnight with the designated antibodies. Afterwards, the PVDF membrane was exposed to an HRP-labelled secondary antibody at room temperature for another 90 min. The bands were finally detected using an enhanced chemiluminescence kit (Abiowell, Changsha, China), and the signals were detected with the ChemiScope imaging ChemiScope6100 system (Millipore, Billerica, MA, United States). ImageJ 1.41 software (Bethesda, MD, United States) was used for the quantitative analysis of western blots.

### 2.5 siRNA transfection

Small interfering RNAs (siRNAs) targeting ATG5, NDP52, OPTN, NBR1, and P62, along with the siRNA Mate transfection reagent, were procured from WellBio (Changsha, Hunan, China). The sequences for ATG5-siRNA, NDP52-siRNA, OPTN-siRNA, NBR1-siRNA, and P62-siRNA are detailed in Table 1. The powders of siRNA targeting the relevant genes were dissolved in diethyl pyrocarbonate (DEPC)-treated water to a final concentration of 20 μM and then stored at −20°C. The ICCs exhibiting good growth were selected, digested with trypsin, and subsequently plated in 6-well plates at a density of 1 × 10^5^ cells/cm^2^. Once the cells reached 60%–80% confluence, preparations for siRNA transfection began. For the transfection, 108 μL of Lipofectamine^®^ RNAiMAX was introduced to 900 μL of Opti-MEM^®^ medium and mixed thoroughly to create the Lipofectamine^®^ RNAiMAX dilution. Separately, 18 μL of siRNAs targeting the relevant genes was added to another 900 μL of Opti-MEM^®^ medium and mixed thoroughly to dilute the siRNAs. Equal volumes of the Lipofectamine^®^ RNAiMAX dilution and the siRNA dilution mixture were then mixed and allowed to incubate at room temperature for 10 min. Next, 300 μL of the siRNA–lipid complexes were introduced into each well of a 6-well plate containing 1.7 mL of complete medium and gently shaken to ensure thorough mixing (the final volume per well was 2 mL, with 18 μL of Lipofectamine^®^ RNAiMAX and an siRNA concentration of 30 nM). The cells were then placed in an incubator at 37°C and cultured for 48–72 h before observation and further experiments.

**TABLE 1:** Sequences of ATG5-siRNA, NDP52-siRNA, OPTN-siRNA, NBR1-siRNA, and P62-siRNA fragments used in the present study.

Gene	Sequence	
	Sense (5′-3′)	Antisense (5′-3′)
R ATG5-343	AGU​AAA​GCA​CGU​UGG​AAU​CCG	GAU​UCC​AAC​GUG​CUU​UAC​UCU
R ATG5-480	UCA​CUA​ACA​UCU​UCU​UGU​CUC	GAC​AAG​AAG​AUG​UUA​GUG​AGA
R ATG5-600	AAA​CUC​UUG​AAA​UGU​ACU​GUG	CAG​UAC​AUU​UCA​AGA​GUU​UUC
R NDP52-675	UAU​CUU​CUG​GUU​GUA​UUC​CUU	GGA​AUA​CAA​CCA​GAA​GAU​AAC
R NDP52-2,860	UGA​CUU​AGU​GUC​AAU​CAG​CUG	GCU​GAU​UGA​CAC​UAA​GUC​AUU
R NDP52-3,253	ACA​AUU​CCU​AAU​UAG​UCA​CUA	GUG​ACU​AAU​UAG​GAA​UUG​UUA
R OPTN-245	AUA​UUG​GAG​GGU​CCA​UUU​CCU	GAA​AUG​GAC​CCU​CCA​AUA​UGG
R OPTN-1092	UUG​AAC​AUU​CAG​AGU​UUC​CAC	GGA​AAC​UCU​GAA​UGU​UCA​AGU
R OPTN-1380	AUU​GUG​UUC​CUG​AAG​AAG​CUU	GCU​UCU​UCA​GGA​ACA​CAA​UAA
R NBR1-260	UUA​CCA​UAG​CUU​CAA​CAU​CAG	GAU​GUU​GAA​GCU​AUG​GUA​AAA
R NBR1-290	UUU​GGA​UAG​UAU​UCA​GAU​CAA	GAU​CUG​AAU​ACU​AUC​CAA​AUA
R NBR1-337	UUG​ACU​AUU​GAU​GGA​UAU​CUC	GAU​AUC​CAU​CAA​UAG​UCA​AGG
R P62-313	UUC​UCU​UUA​AUG​UAG​AUG​CGG	GCA​UCU​ACA​UUA​AAG​AGA​AGA
R P62-1391	UAC​AUG​AUG​CAA​CUA​GAA​GAC	CUU​CUA​GUU​GCA​UCA​UGU​AGA
R P62-1705	UAU​CAG​UUG​UAC​UAA​UCC​CUU	GGG​AUU​AGU​ACA​ACU​GAU​AGU

Upon reaching 60%–70% confluence, the cells were subjected to transfection with either a negative control siRNA (100 nM) or siRNAs targeting ATG5, NDP52, OPTN, NBR1, or P62 (100 nM each) for 6 h using the Lipofectamine 2000 transfection reagent. After transfection, the cells were maintained in complete mesenchymal cell culture medium for subsequent experiments (see Supplementary Figure S2).

### 2.6 Real-time PCR

According to the manufacturer’s guidelines, TRIzol reagent was used to extract total RNA from each group of ICC samples. The extracted total mRNA served as the template for synthesizing cDNA via reverse transcription. The reverse transcription product was suitable for direct use in real-time PCRs for amplification. The primers were designed using Primer five software. The specific sequences of the PCR primers were synthesized by Qingke Biotechnology (Beijing) Co., Ltd., and are listed in Table 2. The quantitative PCR amplification protocol included initial predenaturation at 95°C for 10 min, followed by subsequent cycling steps. The PCR program included denaturation at 95°C for 15 s, and annealing at 60°C for 30 s, which was repeated for a total of 40 cycles. β-Actin served as an internal reference, and target gene expression levels were determined using the relative quantification method. The relative expression was calculated via the 2^−ΔΔCt^ method. Each experiment was conducted in triplicate for each sample.

**TABLE 2:** Primers for qPCR examination.

Gene	Primer sequence	Sequence (5′-3′)	Product length
ATG5	Forward primer Reverse primer	TTGTGCCCCAGCCAACAGAC CATGGAATCTTCTGCCGCCTT	158bp
NDP52	Forward primer Reverse primer	GTCGTGTGCTGTTACACCCT TTTAGGTCATCGGGCATGGG	134bp
OPTN	Forward primer Reverse primer	GGGTTTCCCAGAACCGACTT CAGTTCTGAGACGATGCCCA	138bp
NBR1	Forward primer Reverse primer	GCCTCTGCCCATCCTACAAC AAATCACAACAGGTCTCCGAA	102bp
P62	Forward primer Reverse primer	CTTTGATTTGAGGCACCCCGTA ATTCAACCGCCATGTGCTT	179bp
β-actin	Forward primer Reverse primer	ACATCCGTAAAGACCTCTATGCC TACTCCTGCTTGCTGATCCAC	223bp

### 2.7 Coimmunoprecipitation (Co-IP)

ICCs with a good growth status were selected, and after trypsin digestion, they were inoculated in cell culture plates at a concentration of 1 × 10^5^ cells/cm^2^. Once the confluence reached 80%–90%, the cells were subjected to treatment based on the designated experimental groups. Cell lysis buffer (10×) was diluted at a ratio of 1:10, the corresponding volume of the protease/phosphatase inhibitor mixture was added to prepare the cell lysis solution, and the resulting solution was placed on ice for later use. The cell culture medium was removed, the cells were carefully washed 3 times with precooled PBS, the supernatant was aspirated, and the corresponding volume of precooled cell lysis solution was added. The cell lysate was harvested by scraping the cells and placing them into an EP tube. This mixture was then incubated on ice for 30 min, with gentle mixing every 10 min. Afterwards, centrifugation was performed at 12,000 rpm for 15 min at 4°C. The supernatant was carefully collected into a new EP tube. The protein concentrations in each sample were quantified using the BCA method, and all concentrations were then adjusted to a uniform level of 1 mg/mL. A volume of 100 μL from each sample was combined with 4× loading buffer at a 3:1 volume ratio; after an incubation for 10 min in a metal bath at 95°C, the samples were cooled to room temperature and stored at −80°C as the input. The primary antibody was added to the samples at a ratio of 1:100 (1 μg primary antibody per 100 μL sample), and incubated at 4°C overnight with gentle shaking. The Protein G agarose bead slurry was diluted by one-half with PBS, and 10 μL of the diluted Protein G agarose beads was introduced into each sample and gently mixed, followed by an incubation of 3 h at 4°C. The samples were then subjected to centrifugation at 4°C at 3,000 rpm for 3 min, after which the supernatant was meticulously discarded. Each sample was rinsed 5 times with 1 mL of cell lysis buffer. Subsequently, an appropriate volume of 4× SDS sample buffer was added, and the mixture was incubated in a metal bath at 95°C for 10 min. The processed samples were then loaded onto an SDS‒PAGE gel for Western blot analysis to evaluate the expression levels of the target proteins.

### 2.8 Immunofluorescence staining

For NDP52 and OPTN immunofluorescence staining, the cells were plated into 24-well plates. The cells were incubated in postfixation medium supplemented with 4% paraformaldehyde for 40 min, after which they were washed 3 times with PBS. Next, the cells were permeabilized with 0.1% Triton X-100 for 40 min, followed by blocking in PBS with 5% BSA for 1 h. The ICCs were subsequently incubated overnight at 4°C with primary antibodies against OPTN and NDP52 (diluted 1:100). The following day, the cells were exposed to a FITC-conjugated goat anti-rabbit secondary antibody (diluted 1:50) in the dark for 1 h at room temperature. Next, the samples were washed 3 times with PBS. The ICCs were then counterstained with DAPI (Boster, China) for 10 min. Fluorescence images were subsequently obtained using a fluorescence microscope (Motic, Model: BA210T).

### 2.9 Animals

Each animal study protocol received evaluation and approval from the Institutional Animal Care and Use Committee (IACUC) in compliance with international guidelines. The number of animals utilized in each study was minimized to the necessary number to meet the study objectives, considering the regulatory requirements, statistical power, and historical data availability. All the experimental procedures described in this article were conducted with the utmost consideration for animal welfare. The reporting of animal studies adheres to the ARRIVE guidelines (Kilkenny et al., 2010; McGrath and Lilley, 2015). Male C57BL/6 mice were acquired from Hunan Slake Jingda Experimental Animal Co., Ltd. (Changsha, China) with certificate number SCXK (Xiang) 2019–0004. All the mice were maintained in a controlled environment at 23°C with a 12-h dark/light cycle for 1 week, during which time they had unrestricted access to food and water. All the mice used in our research were operated under a protocol approved by the IACUC of Nanjing University of Chinese Medicine (Ethics approval L20211086. Date: 2021.10.10). The NAR dosing regimens (75, 150, and 300 mg/kg) were selected based on the protocol established by Yin et al. (Yin et al., 2018). The oral gavage administration route was chosen to ensure direct delivery of NAR into the mouse digestive system, thereby mimicking clinical drug absorption *in vivo*. According to the research literature on relevant constipation mouse models (Li et al., 2023; Wen et al., 2023), forty C57BL/6 mice were used for the animal experiment, and the mice were randomly allocated into five groups, comprising eight mice per group: control, Lop, Lop + NAR75, Lop + NAR150, and Lop + NAR300. The STC mouse model was established by administering 10 mg/kg·d loperamide to the mice by gavage once a day for 16 days. The intervention started on the third day of modelling, and the groups, with the exception of the control group, continued to be administered loperamide at the same dose described above. The Lop + NAR groups were treated with 75, 150 or 300 mg/kg NAR twice daily for 2 weeks. The mice in the control group received an equivalent volume of saline. All the mice were sacrificed on the 24th day, and after they had fasted for 12 h, the mice were anaesthetized through an intraperitoneal injection of 1% sodium pentobarbital. Colon tissues and blood from the left ventricle were collected, and the length of the colon was recorded.

### 2.10 Observation of the general condition and faecal characteristics of themice

The fur colour, mental state and activity of the mice were observed daily, and their body weight and food intake were monitored regularly. After the intervention, the mice were fasted but not restricted from water for 16 h. Faecal samples from each mouse were collected within a 6-h period the next day, and the number of particles was recorded. The samples were then weighed, and the wet weight of the faeces was recorded. The fecal samples were transferred to a temperature-controlled box and dried at 90°C for 3 h. After drying, they were weighed again, and the dry weight of the faeces was recorded. The water content of the faeces was determined using the following equation: water content of faeces (%) = (wet weight of faeces‒dry weight of faeces)/wet weight of faeces × 100%.

### 2.11 Measurement of intestinal transit function

A 10% activated carbon suspension was administered by gavage, and the samples were collected after a 30-min interval. The entire small intestine was excised, and the small intestine was peeled off without traction to flatten the tissue; the distance from the mouse pyloric sphincter position to the end of carbon powder propulsion and the total length of the small intestine were measured using a ruler and documented accordingly. The small intestine propulsion rate was calculated using the following formula: small intestine propulsion rate (%) = distance of carbon powder propulsion in the intestine/total length of the small intestine × 100%.

### 2.12 Detection of neurotransmitter concentrations in the serum of mice

After anaesthesia, blood was obtained from the orbital region of each mouse. The samples were permitted to clot at room temperature for 15 min before being centrifuged at 3,000 rpm for 10 min at 4°C. The resulting supernatant was then collected, and the levels of MTL, GAS, SP, VIP, SS, and 5-HT in the mouse serum were assessed using ELISA kits according to the manufacturer’s guidelines.

### 2.13 Histopathological observation of mouse colon tissues

The distal colon tissue from the mice was collected, repeatedly rinsed with saline, fixed with 4% paraformaldehyde, and then subjected to dehydration, clearing, and embedding in wax before being sliced into sections. HE (haematoxylin‒eosin) staining was performed to observe the muscular thickness and pathological changes in the colon, and a semiquantitative analysis of colonic pathological changes in the colon was performed.

The overall morphological scoring of the colonic tissue was as follows (Li et al., 2022; Du et al., 2018): a score of 0 was assigned when the colonic tissue structure was clear and the villi were neatly arranged; a score of one was recorded when the colonic tissue structure was vague but the villi were neatly arranged; and a score of two was assigned when the colonic tissue structure was vague and the villi were disorganized. For epithelial cell shedding, a score of 0 indicated no shedding of colonic epithelial cells; a score of one indicated shedding of colonic epithelial cells without forming sheets; and a score of two indicated shedding of colonic epithelial cells that formed sheets. For inflammatory cell infiltration, a score of 0 was assigned when 0–5 inflammatory cells were observed under the microscope; a score of one was assigned for 6–10 inflammatory cells; and a score of two was assigned when more than 10 inflammatory cells were observed. AB-PAS (Alcian blue–periodic acid–Schiff) staining was used to observe the colon muscle layer, mucosal layer thickness, goblet cell number, mucus area in the mucosal layer and other histological parameters of the colon.

For each section stained with Alcian blue, eight random images were captured at ×400 magnification from different locations. The integrated optical density (IOD) and the effective statistical area were assessed using Image-Pro Plus 6.0 software. Following this analysis, the mean optical density (MOD) was computed, and a subsequent analysis was performed using the average values obtained (Chen K. D. et al., 2024). The mucus layer thickness for each group was assessed using PAS staining.

### 2.14 Ultrastructural examination of colonic ICCs via by transmission electron microscopy

Mouse colon tissue was obtained, cut into 1 mm^3^ blocks, fixed with 2.5% glutaraldehyde, and stored at 4°C. The cut colon tissue was removed, washed with phosphate-buffered saline (PBS), and then fixed with 1% osmic acid for 2 h. Following fixation, the tissue was dehydrated using a gradient of ethanol solutions, impregnated with epoxy resin, and embedded. After ultrathin sectioning, double electron staining with uranyl acetate and lead citrate was performed, and the ultrastructure of the ICCs was examined using a transmission electron microscope (JEM1400, JEOL) and recorded.

### 2.15 Immunohistochemistry

The tissue sections embedded in paraffin were first rehydrated with xylene, followed by a series of graded ethanol solutions. For antigen retrieval, the sections were placed in 0.01 M citrate buffer (pH 6.0) and heated to boiling in either an electric or microwave oven. After boiling for 20 min, the heat was turned off, and the sections were allowed to cool for 20 min before being brought to room temperature. The samples were subsequently rinsed with 0.01 M PBS (pH 7.2–7.6). Periodic acid (1%) was added, and the samples were incubated at room temperature for 10 min to inactivate endogenous enzymes and then rinsed with PBS. Following blocking with normal goat serum, the sections were subjected to immunostaining with primary antibodies against c-Kit (1:300) and ANO1 (1:100) and incubated overnight at 4°C. Then, the sections were rinsed with PBS and subsequently incubated with secondary antibodies for 30 min at room temperature. Afterwards, they were washed with PBS and exposed to the reagents in the DAB kit for 1–5 min at room temperature. Finally, the sections were counterstained with haematoxylin, washed with distilled water, and blued with PBS. For dehydration and transparency, the samples were dehydrated with increasing concentrations of alcohol (60%–100%) for 5 min. After the final alcohol wash, the samples were placed in xylene for 10 min, and this process was repeated twice for thorough clearing. The sections were mounted with neutral gum, and the slides were examined using an optical microscope. All images were captured at a magnification of ×400.

### 2.16 Statistical analysis

All the statistical analyses were performed using GraphPad Prism 9.0 software. The data are shown as the means ± SDs of at least triplicate experiments. One-way ANOVA was used to assess the significance of the statistical findings for comparisons among multiple groups of data. The results were deemed statistically significant when *P* values were <0.05.

## 3 Results

### 3.1 NAR inhibits GA-induced autophagy in ICCs

**FIGURE 1:**
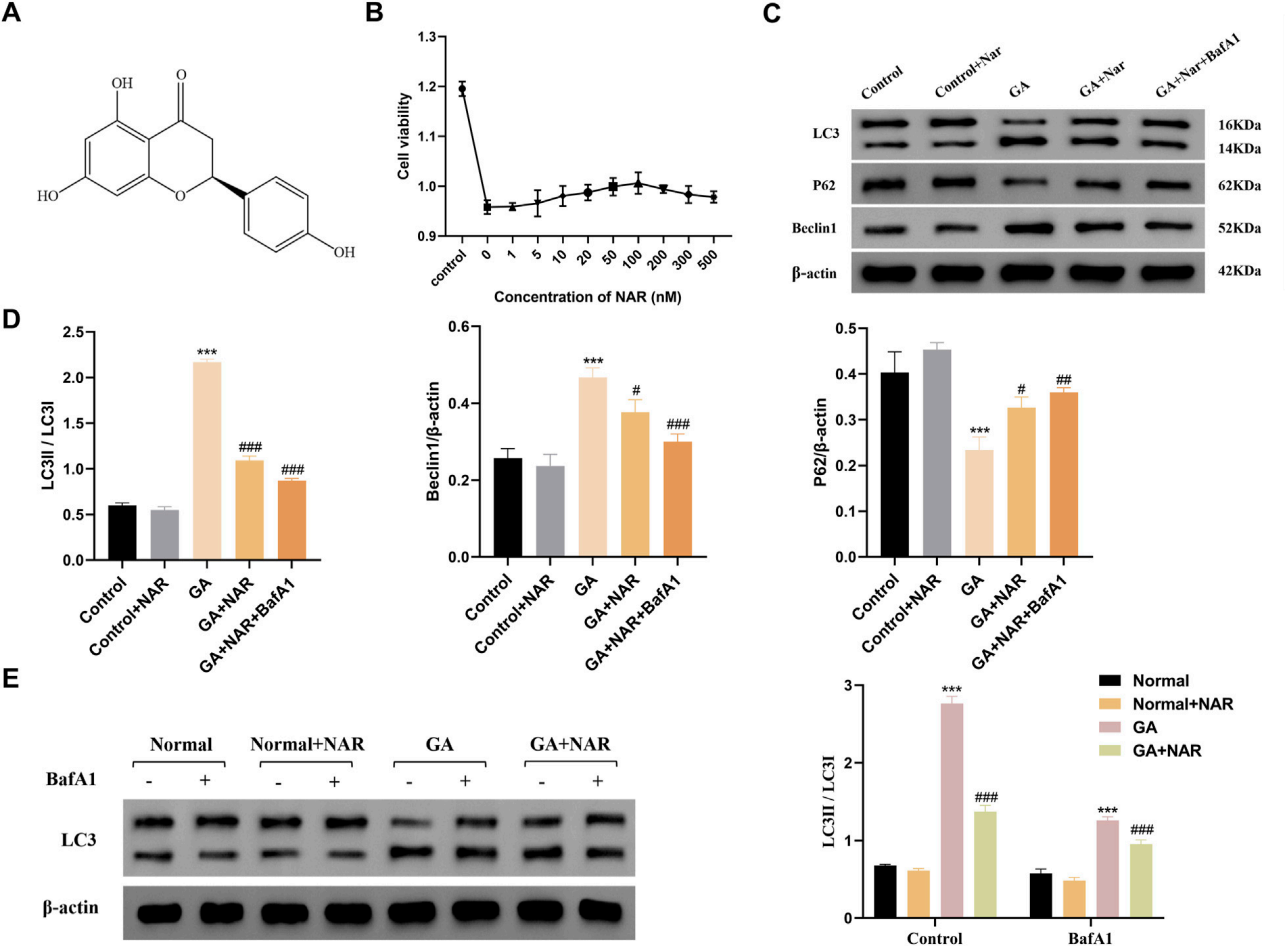
NAR suppressed autophagy in ICCs stimulated by GA. **(A)** Chemical structure of NAR. **(B)** Cytotoxicity of NAR in ICCs (n = 3). **(C,D)** The expression levels of LC3, P62, and Beclin1 were detected by Western blot (n = 3). **(E)** The expression level of LC3 was detected by Western blot (n = 3). Data are presented as the means ± SD. ^***^ *P* < 0.001 vs Control group. ^#^ *P* < 0.05, ^##^ *P* < 0.01, and ^###^ *P* < 0.001 vs GA group. NAR: Naringenin; GA: L- Glutamic acid; BafA1: Bafilomycin A1.

We initially investigated the toxicity of NAR to ICCs to evaluate the cytoprotective function of NAR (Figure 1A). As shown in Figure 1B, the results of the methylthiazolyl tetrazolium assay indicated that NAR had no cytotoxic effects (cell viability greater than 80% of that of the normal control) on ICCs at concentrations less than 100 nM. In the range of 100–500 nM, cell viability decreased with increasing NAR concentrations. Thus, NAR concentrations of 100 nM and 200 nM were selected for the subsequent *in vitro* experiments. Consequently, 100 nM was established as the maximum concentration for further studies. The impact of NAR on autophagy activity was investigated by treating ICCs with GA, and Western blot analysis was subsequently performed to detect the protein expression levels of the autophagy markers LC3, P62, and Beclin1 and analyse the level of autophagy. Compared with the levels in. the control group, the ratios of LC3B II/LC3B I and the expression level of Beclin1 were markedly increased in the GA group, whereas the expression of P62 was noticeably reduced (*p* < 0.001), suggesting that the autophagy activity of ICCs was increased after GA. This effect was attenuated by NAR (*p* < 0.05), indicating that NAR can inhibit autophagy in ICCs under both normal and GA conditions (Figures 1C,D). In addition, we distinguished whether the increases in the levels of autophagy markers in ICCs after GA induction were due to enhanced autophagic activity or disrupted autophagosome degradation by applying the late autophagy inhibitor BafA1 to block the fusion of autophagosomes and lysosomes, namely, to block the autophagic flux, and to observe the changes in the content of LC3II in ICCs after GA treatment and after the naringenin intervention. The total protein extracted from each cell group was subjected to WB to determine the expression level of the LC3 protein. The relative quantitative analysis showed that after BafA1 was administered, the level of LC3II in each group decreased. Compared with the normal group, the GA group presented a more pronounced accumulation of LC3II, suggesting that autophagic flux was markedly increased after GA treatment (*p* < 0.01). Compared with that in the GA group, the accumulation of LC3II in the GA + NAR group was reduced (*p* < 0.01), indicating that NAR treatment weakened the autophagic flux in ICCs after GA exposure (Figure 1E).

### 3.2 Effects of NAR on the survival and apoptosis of ICCs exposed to GA

**FIGURE 2:**
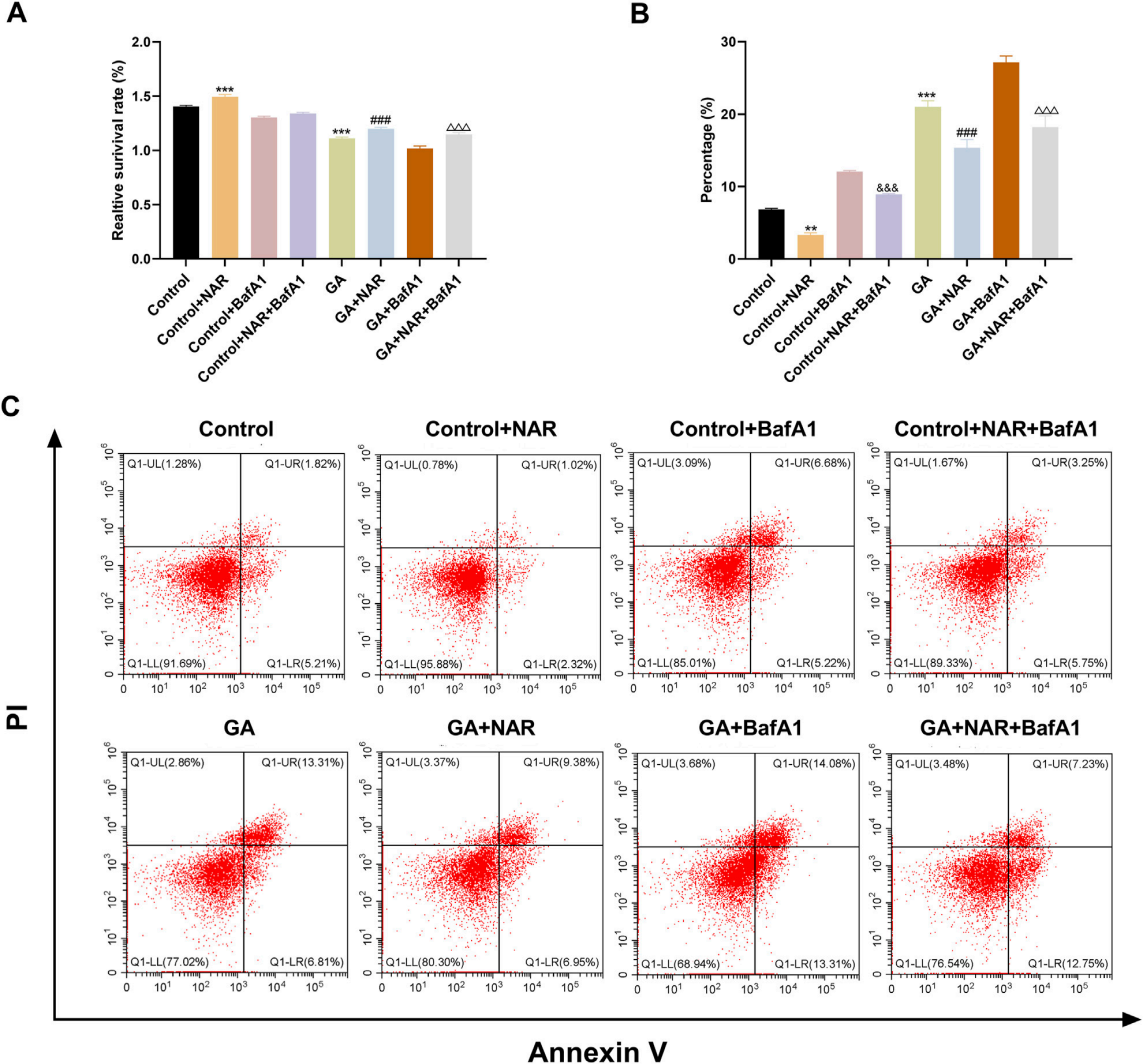
NAR improved the survival and apoptosis of ICCs after GA by suppressing autophagy. **(A)** CCK8 assay was used to determine the survival of ICCs in each treatment group (n = 3). **(B,C)** Flow cytometry (FCM) was used to assess the apoptosis of ICCs in each treatment group (n = 3). Data are presented as the means ± SD. ^**^ *P* < 0.01, ^***^ *P* < 0.001 vs. Control group. ^###^ *P* < 0.001 vs. GA group. ^&&&^ *P* < 0.001 vs Control + BafA1 group. ^△△△^ *P* < 0.001 vs GA + BafA1 group. NAR: Naringenin; GA: L- Glutamic acid; BafA1: Bafilomycin A1.

We initially employed the CCK8 method to assess the survival of ICCs across each group, to investigate the impact of autophagy on the survival and apoptosis of ICCs following GA treatment, and to understand the regulatory role of NAR. Figure 2A shows that the survival rate of ICCs in the GA group was noticeably reduced (*p* < 0.01) compared with that in the control group. After the autophagy inhibitor BafA1 was administered to the GA group, the cell survival rate further increased to approximately 44%, and the difference was statistically significant (*p* < 0.01), indicating that blocking autophagy markedly increased the survival of ICCs following GA treatment (*p* < 0.05). In addition, compared with that of the GA group, the cell survival rate of the GA + NAR group increased to approximately 79% (*p* < 0.01), and compared with that of the GA + NAR group, the cell survival rate of the GA + NAR + BafA1 group increased to approximately 88% (*p* < 0.05). Flow cytometry was subsequently used to evaluate the degree of ICC apoptosis in each treatment group. We investigated how autophagy influences the activity of ICCs after GA treatment and the regulatory effect of NAR on this activity. As shown in Figures 2B,C, compared with that in the control group, the apoptosis rate of ICCs in the GA group was markedly increased (*p* < 0.01). NAR treatment significantly reduced the rate of ICC apoptosis (*p* < 0.01). The results presented above suggested that NAR improved the survival rate of ICCs and reduced their apoptosis rate by inhibiting autophagy.

### 3.3 NAR attenuates autophagy in ICCs after GA treatment by inhibiting the AMPK/mTOR/ULK1 signaling pathway

**FIGURE 3:**
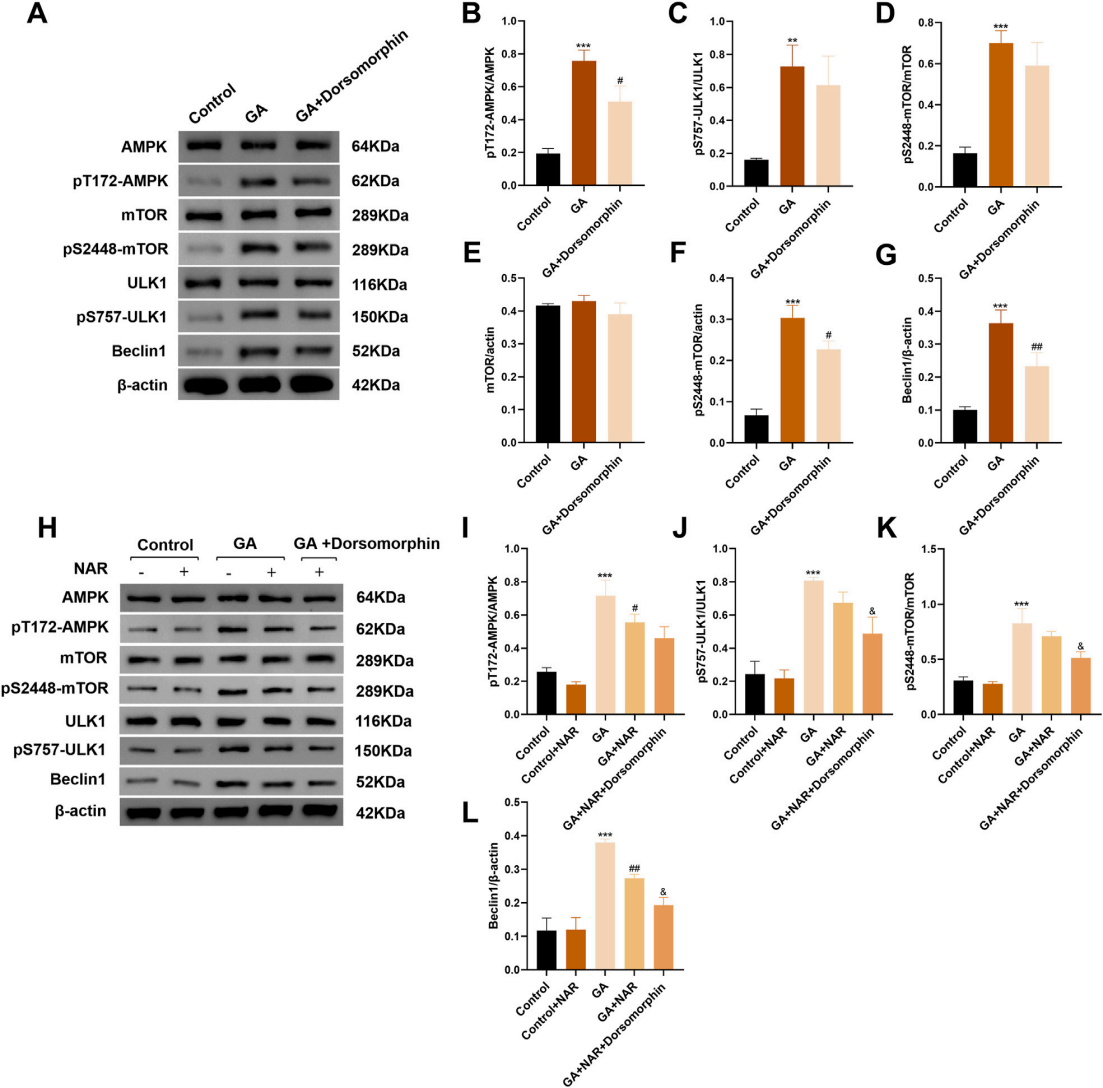
NAR influenced autophagy by inhibiting the activation of the AMPK/mTOR/ULK1 signaling pathway in ICCs after GA. **(A–L)** Western blot was used to assess the expression of AMPK, mTOR, ULK1 and their phosphorylated proteins (n = 3). Data are presented as the means ± SD. ^**^ *P* < 0.01, ^***^ *P* < 0.001 vs. Control group. ^#^ *P* < 0.05, ^##^ *P* < 0.01 vs. GA group. ^&^ *P* < 0.05 vs. GA + NAR group. NAR: Naringenin; GA: L- Glutamic acid.

The AMPK protein plays a vital role in monitoring and maintaining energy homeostasis at both the cellular and systemic levels. Under conditions of stress, AMPK activation can inhibit the activity of mTOR, a critical regulator of autophagy and apoptosis, thereby promoting autophagy (Chen Y. F. et al., 2024). The initiation of autophagy relies mainly on the ULK1 complex. mTOR binds to serine 757 of p-ULK1 to inhibit the initiation of autophagy (Wei et al., 2023). Therefore, we performed Western blotting to measure the expression levels of AMPK, mTOR, and ULK1 and their phosphorylated proteins in ICCs from each treatment group, observed changes in the AMPK/mTOR/ULK1 pathway after GA treatment and its effect on the level of autophagy in ICCs. In the GA group, the ratios of pT172-AMPK/AMPK and pS757-ULK1/ULK1 were markedly increased (*p* < 0.05) (Figures 3A–C), the mTOR protein level was slightly decreased, the pS2448-mTOR protein level was noticeably decreased (*p* < 0.05) (Figures 3A,E,F), and the ratio of pS2448-mTOR/mTOR decreased (*p* < 0.05) (Figures 3A,D). Concurrently, the expression level of the autophagy marker Beclin1 was notably increased (*p* < 0.05) (Figures 3A,G), indicating that the AMPK/mTOR/ULK1 pathway was activated and that autophagy activity was increased in ICCs after GA treatment. After treatment with the AMPK inhibitor dorsomorphin, the ratios of pT172-AMPK/AMPK and pS757-ULK1/ULK1 were markedly decreased (*p* < 0.05), whereas the ratios of pS2448-mTOR/mTOR were dramatically increased (Figures 3A–D), indicating that the increase in autophagic activity in ICCs after GA treatment may be mediated by the AMPK/mTOR/ULK1 pathway. As a method to verify whether NAR inhibits autophagy in ICCs after GA treatment via the AMPK/mTOR/ULK1 signalling pathway, we performed Western blotting to assess the expression levels of AMPK, mTOR, and ULK1 and their phosphorylated proteins in the ICCs of each group. Compared with those in the GA group, the pT172-AMPK/AMPK and pS757-ULK1/ULK1 ratios in the GA + NAR group were significantly reduced, whereas the pS2448-mTOR/mTOR ratio was increased (Figures 3H–K). Moreover, the expression of autophagy marker Beclin1 was noticeably decreased (Figures 3H,L). After treatment with the AMPK inhibitor dorsomorphin, the abovementioned changes in each group were further amplified (Figures 3H–L). These results indicated that NAR inhibited autophagy by inhibiting the activation of the AMPK/mTOR/ULK1 pathway in ICCs following GA treatment.

### 3.4 The mechanism of pS757-ULK1 degradation induced by glutamate in ICCs and the regulatory effect of NAR on pS757-ULK1 degradation

Cellular autophagy is a critical process that enables the body to resist stress and increase survival. The formation and activation of the ULK1-ATG13-FIP200 complex is a crucial step in autophagy initiation. During nutrient deficiency, activated AMPK directly phosphorylates several sites on ULK1, thereby triggering autophagy (Kim et al., 2011; Wang and Jia, 2023). Nonetheless, mTOR, a cell growth regulator and autophagy inhibitor, can phosphorylate ULK1 at Ser757. The cell can interfere with the interaction between ULK1 and AMPK, which in turn prevents autophagy (Wang et al., 2019). Hence, the phosphorylation of Ser757 on ULK1 is crucial for the role of mTOR in regulating autophagy, with the level of pS757-ULK1 being closely linked to the initiation of autophagy (Wang et al., 2018). Nevertheless, previous studies have been limited to assessing the regulation of autophagy by pS757-ULK1, and whether changes in the pS757-ULK1 content are affected by the overall level of cellular autophagy remains unclear. In this study, we examined the underlying mechanism of pS757-ULK1 in ICCs after glutamate induction, explored whether it could be a target for the treatment of slow-transit constipation, and provided a stronger theoretical foundation for the clinical application of NAR.

#### 3.4.1 Level of pS757-ULK1 in ICCs induced by glutamate through autophagy and the role of NAR in its regulation

**FIGURE 4:**
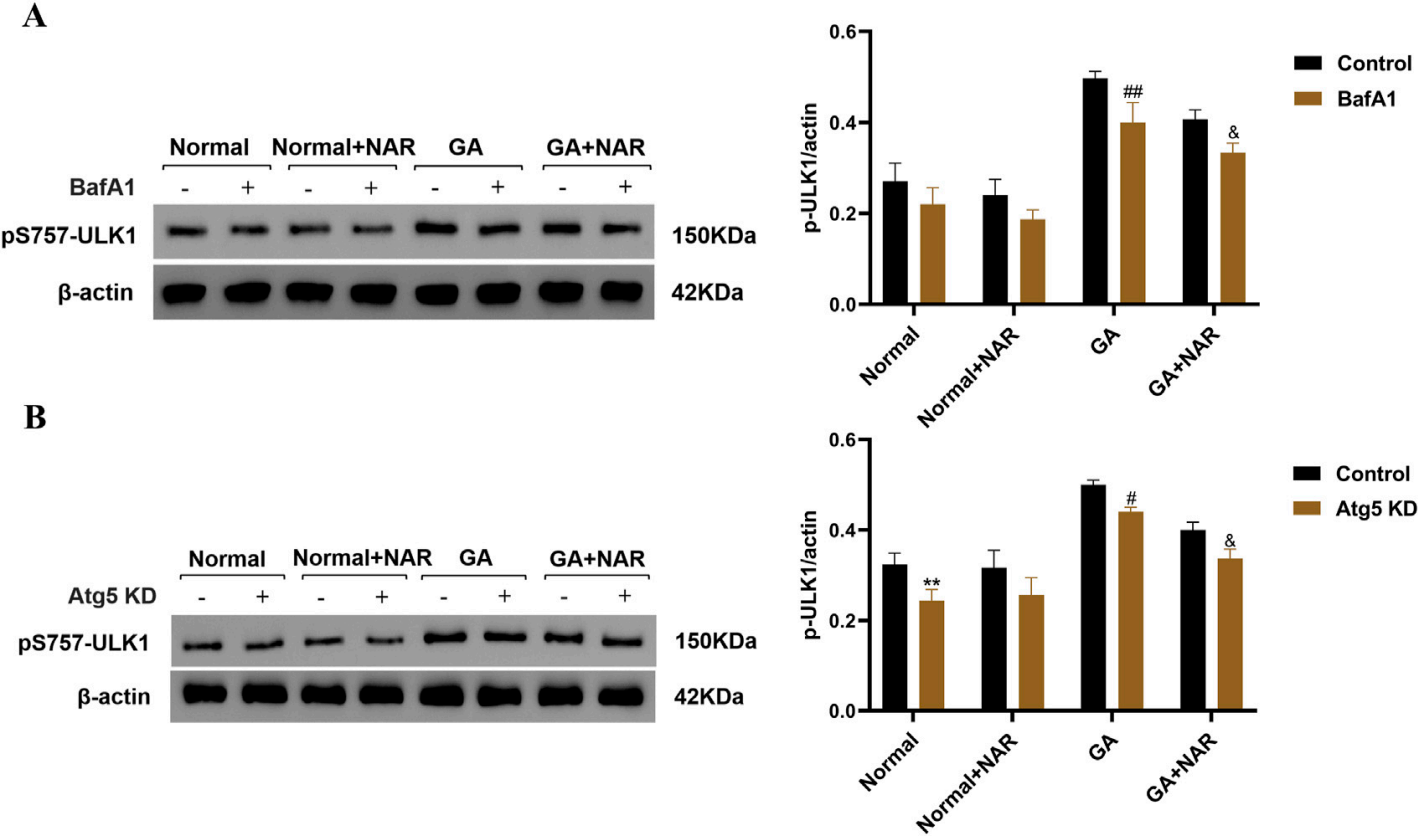
NAR inhibited the autophagic degradation of pS757-ULK1 in ICCs after GA. **(A)** Before and after treatment with the autophagy inhibitor BafA1, the expression level of pS757-ULK1 was detected by Western blotting (n = 3). **(B)** After knockdown of the autophagy-related gene Atg5 in murine ICCs, the expression level of pS757-ULK1 was detected by Western blotting (n = 3). Data are presented as the means ± SD. ^**^ *P* < 0.01 vs. Control group. ^#^ *P* < 0.05, ^##^ *P* < 0.01 vs. GA group. ^&^ *P* < 0.05 vs. GA + NAR group. NAR: Naringenin; GA: L- Glutamic acid; BafA1: Bafilomycin A1.

BafA1 is a common late-stage autophagy inhibitor that can inhibit cellular autophagy by blocking the combination of lysosomes and autophagosomes. We investigated the relationship between the decrease in pS757-ULK1 protein levels and autophagy in the ICCs of each treatment group by performing Western blotting to assess the variations in pS757-ULK1 expression levels in each group of ICCs before and after the autophagy inhibitor BafA1 was administered to observe whether it accumulated and to determine whether the pS757-ULK1 protein was degraded through autophagy. The relative quantitative analysis revealed no statistically significant difference in the expression level of pS757-ULK1 in the normal group before and after treatment with BafA1 (ns). In the GA group, the expression level of pS757-ULK1 decreased (*p* < 0.01), whereas in the GA + NAR group, pS757-ULK1 expression was markedly reduced (*p* < 0.05), with a further obvious decrease upon NAR treatment (Figure 4A). Furthermore, at the gene level, when siRNA technology was used to knock down the autophagy-related gene Atg5 in mouse ICCs, a relationship between decreases in pS757-ULK1 protein levels and autophagy was observed across treatment groups. The relative quantitative analysis indicated that after Atg5 gene knockdown, pS757-ULK1 expression levels decreased (*p* < 0.05), with a more pronounced reduction observed upon NAR treatment (*p* < 0.05) (Figure 4B). These findings suggested that pS757-ULK1 did not accumulate in astrocytes from each group after Atg5 knockdown, and NAR treatment led to a significant decrease in pS757-ULK1 levels.

#### 3.4.2 Relationship between pS757-ULK1 expression and selective autophagy in glutamate-treated ICCs and the regulatory role of NAR

**FIGURE 5:**
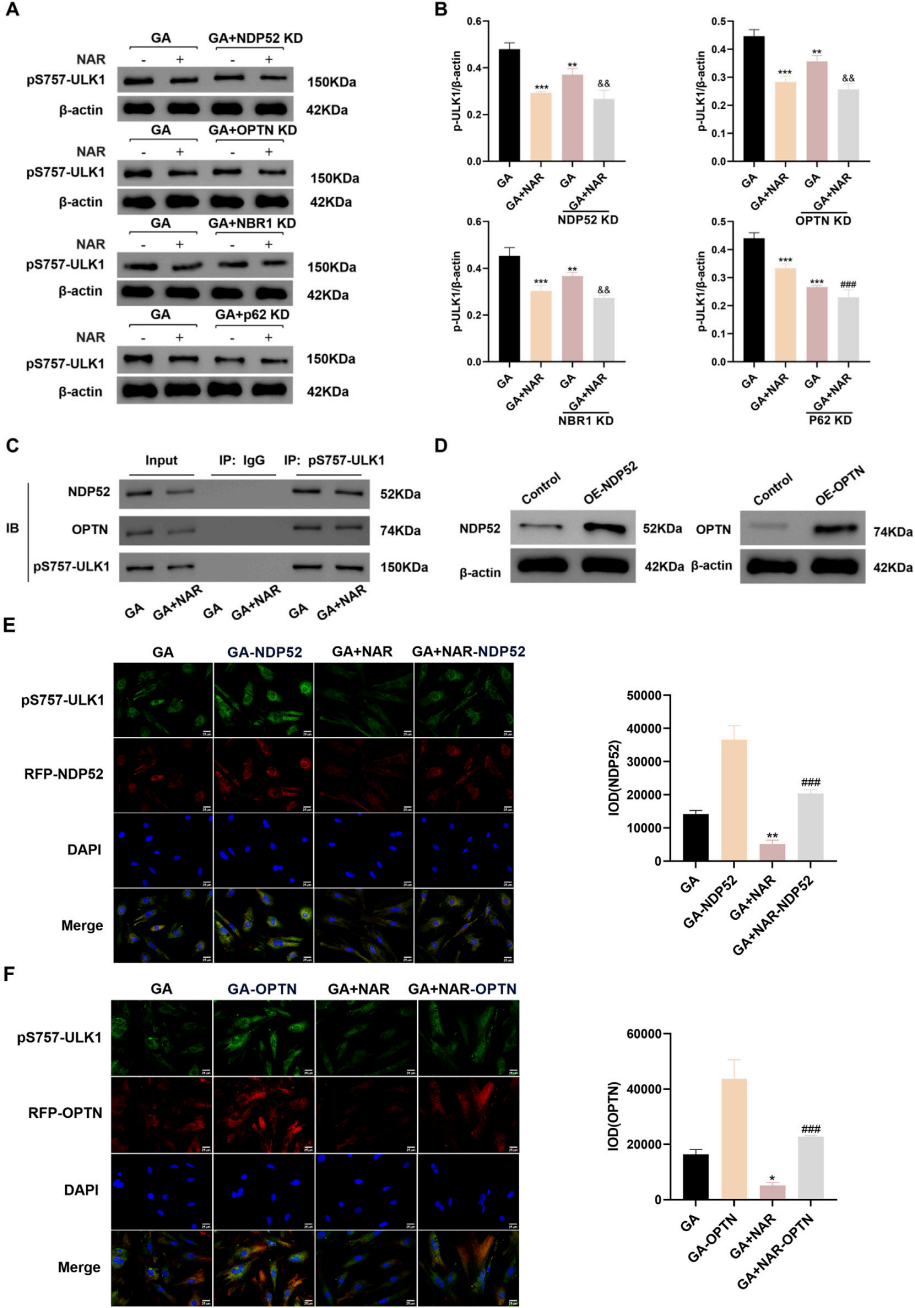
NAR inhibited autophagic activity in ICCs after GA by suppressing the interaction between pS757-ULK1 and the selective autophagy receptor genes NDP52 and OPTN. **(A,B)** After knockdown of the selective autophagy receptor gene NDP52, OPTN, NBR1 and p62, Western blot was used to assess the expression levels of pS757-ULK1 protein (n = 3). **(C)** Co-immunoprecipitation was performed to evaluate the interactions between pS757-ULK1 and NDP52, as well as between pS757-ULK1 and OPTN. **(D)** The expression levels of NDP52 and OPTN were detected by Western blot. **(E,F)** Immunofluorescence staining was performed to observe the co-localization of pS757-ULK1 with NDP52 and OPTN, and representative images are displayed (n = 3). Data are presented as the means ± SD. ^*^ *P* < 0.05, ^**^ *P* < 0.01, and ^***^ *P* < 0.001 vs. Control group. ^###^ *P* < 0.001 vs. GA + NAR group. ^&&^ *P* < 0.01 vs. GA + KD group. NAR: Naringenin; GA: L- Glutamic acid; NDP52: Nuclear dot protein 52 kDa; OPTN: Optineurin; NBR1: Neighbor of BRCA1 gene one protein.

We investigated the relationship between pS757-ULK1 and selective autophagy by employing siRNA technology to knock down the genes encoding the selective autophagy receptors NDP52 (nuclear dot protein 52 kDa, NDP52), OPTN (optineurin, OPTN), NBR1 (neighbour of BRCA1 gene one protein, NBR1) and p62. As shown in Figure 5, before NDP52, OPTN, NBR1, and p62 gene knockdown, unlike in the GA group, the expression level of the pS757-ULK1 protein in ICC was significantly reduced, and the expression level of the pS757-ULK1 protein in the GA + NAR group was noticeably decreased (*p* < 0.01) (Figures 5A,B). After NDP52 and OPTN gene knockdown, the pS757-ULK1 protein expression level in the GA + NAR group was markedly reduced compared with the GA group (*p* < 0.01). In the GA + NAR group, after NDP52 and OPTN gene knockdown, the expression level of the pS757-ULK1 protein was dramatically reduced (*p* < 0.01). In contrast to that in the GA group, the expression level of the pS757-ULK1 protein in the GA + NAR group was noticeably reduced (*p* < 0.01). In the GA + NAR group, after NBR1 and p62 gene knockdown, the expression level of the pS757-ULK1 protein did not change markedly (*p* > 0.05). The evidence suggested that pS757-ULK1 protein expression in ICCs after GA treatment was related to the selective autophagy receptors NDP52 and OPTN but not to NBR1 or p62 and that NAR decreased the levels of the pS757-ULK1 protein through NDP52 and OPTN. Furthermore, we investigated the effect of NAR on the interaction between pS757-ULK1, NDP52, and OPTN in ICCs following GA treatment. The co-IP analysis revealed that NAR treatment markedly attenuated the interaction between pS757-ULK1 and NDP52 as well as OPTN (Figure 5C). Moreover, cell transfection technology was used to overexpress the selective autophagy receptor genes NDP52 and OPTN in ICCs (see Figure 5D for the transfection of transfection). Immunofluorescence staining revealed that NAR treatment significantly weakened the protein fluorescence intensity, and the degree and quantity of colocalization of pS757-ULK1 with RFP-NDP52 and RFP-OPTN were also noticeably reduced (Figures 5E,F). These results suggest that NAR reduces the levels of pS757-ULK1 by weakening the interactions between pS757-ULK1 and NDP52 and OPTN, thereby further inhibiting the autophagic activity of ICCs after GA treatment.

### 3.5 Effect of NAR on defecation in mice with lop-induced constipation

**FIGURE 6:**
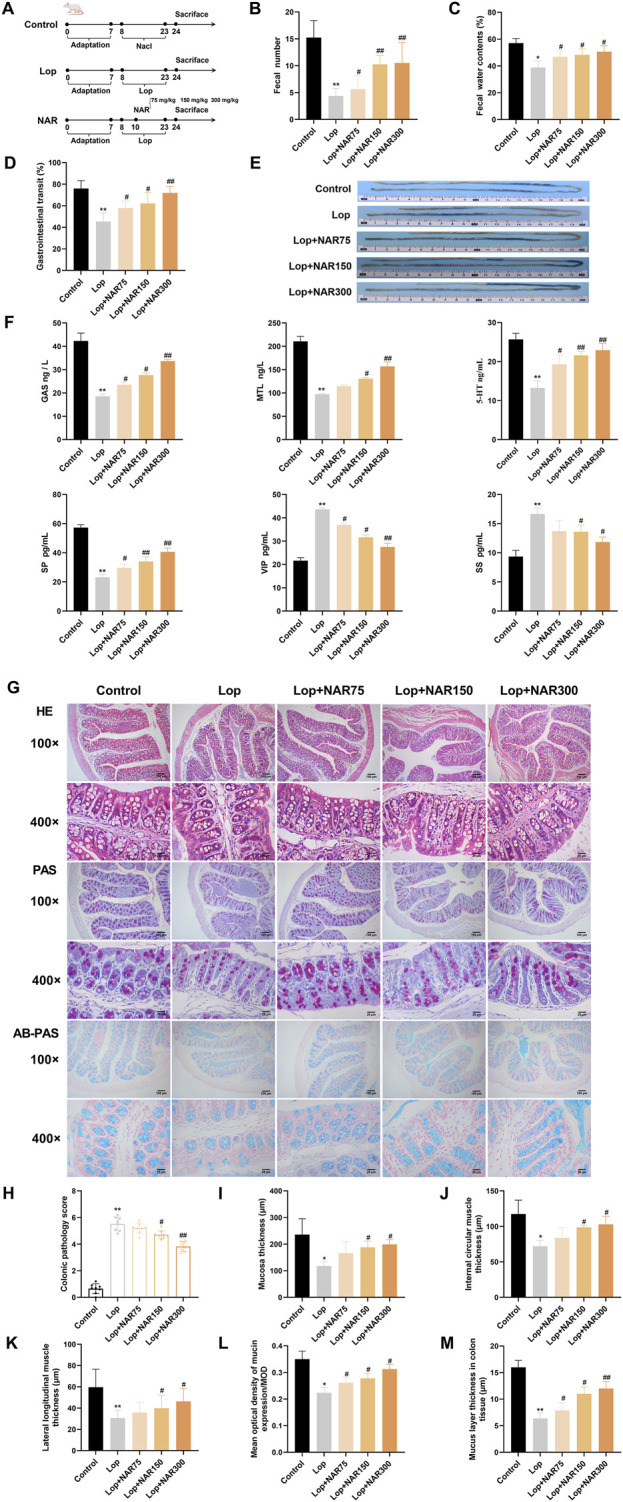
NAR alleviates loperamide-induced constipation in mice. Constipation was induced by administering Lop intragastrically, followed by treatment with or without NAR. **(A)** Flow chart of the establishment of the mouse model and drug administration. During the experiment, the following parameters were measured simultaneously: **(B)** fecal quantity, **(C)** fecal water content, and **(D,E)** small intestinal propulsion rate (n = 8). The length of charcoal from stomach in the intestine was measured after the administration of charcoal. Charcoal transit ratio (%) = (distance travelled by the charocal)/(total length of small intestine) × 100%. **(F)** Detection of neurotransmitter concentrations in the serum of mice (n = 3). **(G)** HE staining and AB-PAS staining were used to observe morphological changes in the colonic tissue of mice (magnification = ×100 , 400 ×) **(H)** Colonic pathology score of mice in each group (n = 8). **(I)** Changes of mucosal layer thickness in colon tissue of mice in each group (n = 6). **(J,K)** Changes of the thickness of Internal circular muscle layer and lateral longitudinal muscle layer in mice of each group (n = 6). **(L)** Mean optical density of mucin expression/MOD (n = 8). **(M)** Changes of mucus layer thickness in colon tissue of mice in each group (n = 8). Data are presented as the means ± SD. ^*^ *P* < 0.05, ^**^ *P* < 0.01 vs. Control group. ^#^ *P* < 0.05, ^##^ *P* < 0.01 vs. Lop group. NAR: Naringenin; Lop: loperamide; Gas: gastrin; MTL: motilin; 5-HT: 5-hydroxy tryptamine; SP: substance P; VIP: vasoactive intestinal peptide; SS: somatostatin.

Throughout the experiment, no mice died. Before model induction, the mice in each group exhibited glossy fur; normal activity, eating, and drinking habits; and moderately dry and hard stools. We primarily evaluated constipation by assessing the faecal pellet count, water content, and small intestinal transit rate to assess the effects of NAR on loperamide-induced constipation. A flow chart illustrating the modelling and drug administration process is shown in Figure 6A. Compared with mice in the normal group, the mice in the Lop group presented markedly reduced faecal pellet counts, decreased water contents, and lower small intestinal transit rates (*p* < 0.01). Compared with the mice in the Lop group, the quantity of faecal particles of mice in the NAR treatment exhibited an was lower but showed an overall increasing trend, and the quantity of faecal particles of mice in the Lop + NAR300 group reached the level of that in the Lop group (*p* < 0.01). Additionally, the NAR treatment group increased faecal water content and small intestinal transit rate to varying degrees (*p* < 0.05, Figures 6B–E). These results suggest that NAR treatment could ameliorate the altered excretory parameters in mice with Lop-induced constipation.

### 3.6 Effects of NAR on serum neurotransmitters levels in mice

Gastrointestinal peptides can generally be categorized into two types: excitatory and inhibitory (Mu et al., 2020). MTL, GAS, 5-HT, and SP are excitatory gastrointestinal peptides (Shen et al., 2024), whereas SS and VIP are inhibitory gastrointestinal peptides (Zhang et al., 2023). This study investigated whether NAR restores intestinal function by influencing the levels of peptide hormones in the host. Compared with those in the control group, the levels of MTL, GAS, 5-HT, and SP in the serum of the model group were notably decreased (*p* < 0.01), whereas the levels of SS and VIP were markedly increased. Compared with those in the model group, the levels of MTL, GAS, 5-HT, and SP in the serum of the mice in the NAR medium- and high-dose groups were clearly increased, whereas the levels of SS and VIP were significantly decreased (*p* < 0.05, *p* < 0.01) (Figure 6F). These findings indicate that NAR has the ability to modulate the levels of gastrointestinal neurotransmitters to some extent and improve the reduction in gastrointestinal motility in mice with constipation induced by loperamide hydrochloride.

### 3.7 Effects of NAR on colonic pathology in mice with lop-induced constipation

Under a light microscope, the colon tissue of the mice in the blank control group showed no significant abnormalities, with abundant intestinal glands, neatly arranged villi, and signs of inflammatory cell infiltration. In the Lop group, the colonic structure was unclear, the glands were atrophied, the villi were disorganized, and some mucosal epithelium was shed, with a visible inflammatory exudate. The NAR groups showed varying degrees of improvement in these pathological changes in the colon (Figure 6G).

A semiquantitative analysis of the overall colonic morphology, mucosal epithelial cell shedding, and inflammatory cell infiltration revealed that the pathological scores of the colons in the Lop group were markedly greater than those in the blank control group (*p* < 0.01). Compared with those in the Lop group, the pathological scores of the NAR group markedly decreased, with both the medium- and high-dose groups displaying statistically significant differences (*p* < 0.05, *p* < 0.01) (Figure 6H). Compared with those in the control group, the thicknesses of the external longitudinal muscle layer, inner circular muscle layer and mucosal layer of the colon in the Lop-treated group mice were notably decreased (*p* < 0.05); in contrast to those in the Lop group, the same parameters in the high- and medium-dose NAR groups were markedly increased (*p* < 0.05) (Figures 6I–K). PAS staining revealed that the mucosal layer thickness in the Lop group was meaningfully decreased compared with that in the blank group after modelling. After various doses of NAR were administered, the differences in the thickness of the colonic mucus layer in yhe mice were statistically significant.

### 3.8 Impact of NAR on colonic mucus secretion in mice with lop-induced constipation

Constipation can lead to increased colonic mucosal permeability, reduced goblet cell numbers, and decreased mucus secretion, thereby compromising the intestinal mucus barrier (Wang J. K. et al., 2022; Baidoo and Sanger, 2024). Alcian blue staining was used to stain mucin-producing goblet cells blue (Figure 6G).

Compared with those in the control group, the number of goblet cells and the mucus area in the colon tissue of the Lop-treated group were markedly decreased. After the NAR intervention, the number of goblet cells and the mucus area in the colon tissue of the mice in the low-, middle- and high-dose NAR groups were notably increased (*p* < 0.001, Figure 6). These findings indicated that after the NAR intervention, the quantity of goblet cells in the colon tissue of the mice increased, mucus secretion increased, and the intestinal mucus barrier was repaired. The improvement was greater in the high-dose group (Figures 6L,M).

### 3.9 Effects of NAR on the ultrastructure of the colon in mice with lop-induced constipation

**FIGURE 7:**
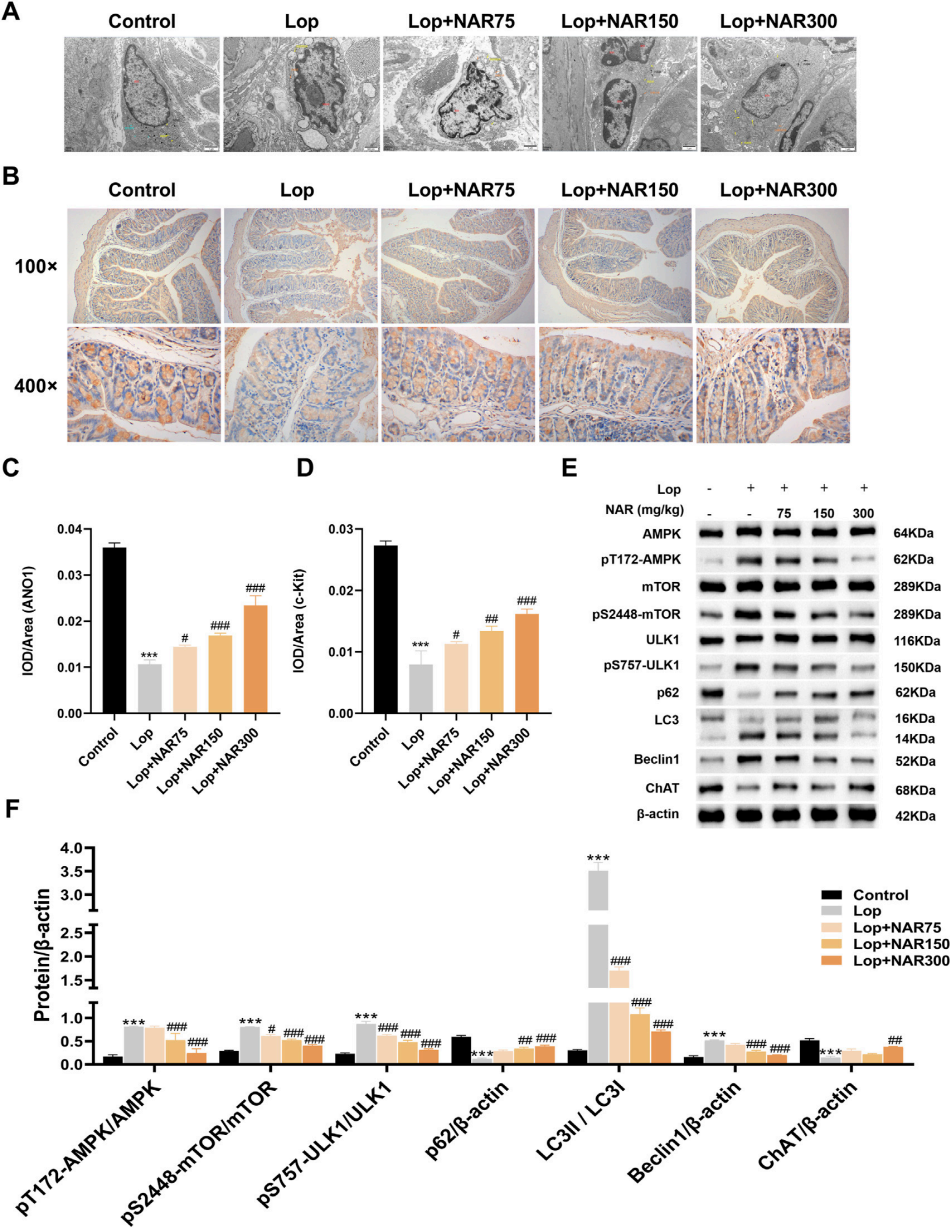
Effect of NAR on AMPK/mTOR/ULK1 signaling pathway in colon tissues of Lop-induced constipated mice. **(A)** Evaluation of cellular microstructures in the colonic mucosa by transmission electron microscopy. **(B)** Immunohistochemical analysis was performed to detect the expression of ANO1 and c-Kit in the colonic tissue of Lop-induced constipation mice (magnification = × 400). **(C,D)** The IOD value of immunohistochemical positive signals (n = 3). **(E,F)** The expression levels of AMPK, pT172-AMPK, mTOR, pS2448-mTOR, ULK1, pS757-ULK1, P62, LC3II/I, Beclin1, and ChAT were detected by Western blot (n = 3). Data are presented as the means ± SD. ^***^ *P* < 0.001 vs. Control group. ^#^ *P* < 0.05, ^##^ *P* < 0.01, and ^###^ *P* < 0.001 vs Lop group. TEM: transmission electron microscopy; NAR: Naringenin; Lop: loperamide.

The colon tissue of the normal group of mice exhibited a complete cell morphology and structure, regular nuclei, and abundant organelles (mitochondria, the endoplasmic reticulum, and the Golgi apparatus). Unlike those in the normal group, the cells in the Lop group presented partial membrane rupture, an incomplete cell structure, slight swelling of mitochondria, an obvious crista structure, sparse degradation of some mitochondria, vacuolation, increased numbers of autophagosomes and autophagic mitochondria, and overall shrinkage of cells, which were smaller than those in the blank group. Unlike the Lop group, the NAR treatment group exhibited a dramatic improvement, with the Lop + NAR300 group exhibiting the most notable changes. The cell morphology was better, organelle structures were more intact, the mitochondrial volume was increased, and the quantity of autophagosomes was noticeably decreased (Figure 7A).

### 3.10 Expression levels of the ANO1 and c-kit proteins in the colon tissue of mice with lop-induced constipation that were treated with NAR

ICCs are associated with the progression of numerous gastrointestinal motility disorders, such as idiopathic STC. ANO1 is closely associated with the generation of ICC pacemaker currents and can regulate the excitability of intestinal smooth muscle in patients with constipation (Kashyap et al., 2011). c-Kit is considered a distinct marker of ICC and promotes the proliferation and growth of ICCs by binding to the receptor SCF (Zhang et al., 2021; Tong et al., 2005). In this study, IHC was used to assess the impact of NAR on ICCs, with a focus on changes in ANO1 and c-Kit protein levels in the colons of mice with Lop-induced constipation after they were treated with NAR. The experimental data suggested that ANO1 and c-Kit were expressed mainly in the apical and lateral mucosal epithelial cells of the colon tissue. Compared with the normal group, the Lop group presented decreased levels of ANO1 and c-Kit in the colon tissue of constipated mice (*p* < 0.001). However, this phenomenon was attenuated after NAR treatment, and Lop + NAR300 had the most significant effect (*p* < 0.001). NAR increased the levels of ANO1 and c-Kit in the colon tissue of mice with Lop-induced constipation mice (Figures 7B–D).

### 3.11 NAR regulates the expression of vital factors in the AMPK/mTOR/ULK1 pathway in the colon tissue of mice with lop-induced constipation

Unlike those in the normal group, the ratios of pT172-AMPK/AMPK, pS757-ULK1/ULK1 and LC3Ⅱ/Ⅰ and the level of the autophagy marker Beclin1 in the colon tissue of Lop-treated group of mice were dramatically increased, whereas the levels of pS2448-mTOR/mTOR, p62 and ChAT were markedly reduced (*p* < 0.001). Compared with those in the Lop group, the ratios of pT172-AMPK/AMPK, pS757-ULK1/ULK1 and LC3Ⅱ/Ⅰ and the expression levels of the autophagy marker Beclin1 in the NAR treatment group were noticeably reduced, whereas the levels of pS2448-mTOR/mTOR, p62 and ChAT showed increasing trends (*p* < 0.05, Figures 7E,F).

## 4 Discussion

STC is characterized by reduced intestinal transit function and is a type of refractory constipation with a complex pathogenesis. It is commonly thought to be linked to factors such as enteric nervous system disorders, ICC damage, abnormal gastrointestinal hormone levels, neurotransmitter imbalances, gut microbiota dysbiosis, psychological factors, and abnormalities in the morphology and function of the intestinal smooth muscle (Huizinga et al., 2021; Tian et al., 2021; Tian et al., 2020). Research has shown that colonic ICCs from individuals with STC have fewer synapses, damaged network-like structures, and increased numbers of autophagosomes. Severe cellular autophagy can lead to smooth muscle contraction and dysmotility, which in turn causes colon conduction dysfunction (Wu et al., 2020). Therefore, decreasing autophagy in STC colonic ICCs is a crucial approach for treating STC.

Currently, the commonly used medications for treating STC include prokinetics, stimulant laxatives, and osmotic laxatives, which have unsatisfactory clinical efficacy and significant side effects (Sailer, 2022). Dietary fibre is recommended as a cornerstone therapy for chronic constipation (Wong and Lubowski, 2007), as increasing dietary fibre intake can reduce the incidence of constipation (Schmier et al., 2014). However, studies also present contrary viewpoints (Markland et al., 2013). Numerous clinical studies have confirmed that acupuncture effectively treats STC by promoting intestinal motility and improving symptoms related to defecation difficulties (Peng et al., 2012). However, the pain caused by needle insertion makes patients fearful and increases the difficulty of maintaining periodic treatment. In addition, each treatment requires an appointment with a specialist to select acupoints based on individual conditions, which is time-consuming and laborious. The clinical effects of internal medicine and nondrug therapies on treating STC patients are poor. In addition, their practicality and efficacy in treating STC are hindered by several factors, such as the optimal dosage, treatment duration, and safety issues.

STC patients often choose surgical treatment, but postoperative complications such as infection, intestinal adhesion, and ileal reflux are also more common (Perivoliotis et al., 2022), which causes considerable trauma to patients both physically and psychologically. The attributes of traditional Chinese medicine (TCM) in addressing diseases involve multiple components, various pathways, and numerous targets, and its limited side effects determine its advantages in clinical treatment (Zhou et al., 2024; Wang L. et al., 2022). He et al. discovered that astragaloside IV treatment for STC increased ICC proliferation, modulated the gut microbial structure and butyric acid production, and regulated the protein kinase B–nuclear factor–kappa B (AKT-NF-κB) pathway, which is involved in inflammatory factor expression (He et al., 2020). Kim et al. reported that quercetin promotes faecal excretion and increases gastrointestinal motility, simultaneously ameliorating colonic tissue pathology in STC rats. This outcome is facilitated by the modulation of muscarinic acetylcholine receptors (mAChRs) and an increase in mucin secretion (Kim et al., 2018). NAR has pharmacological effects, including antiatherosclerotic, hepatoprotective, anticancer properties, and anti-inflammatory effects (Ekambaram et al., 2008; Renugadevi and Prabu, 2010). Yang et al. discovered that NAR induces Cl^−^ secretion in the colonic epithelium by engaging the signaling pathways of cyclic adenosine monophosphate (cAMP) and protein kinase A (PKA) (Yang et al., 2008) signalling pathways. Similarly, Yin et al. showed that NAR mitigated constipation in mice by modulating the expression of ICC markers, including c-Kit and stem cell factor (SCF), as well as aquaporin 3 (AQP3) (Yin et al., 2018). As explained above, NAR treatment has a certain scientific basis, but whether there are other potential specific treatment mechanisms remains unclear.

ICCs are situated mainly between the colonic nervous system and the smooth muscle layer, where they function as pacemaker cells, generating slow waves that regulate gastrointestinal smooth muscle contractions and participating in their formation and mediating enteric nervous signalling to regulate gastrointestinal muscle contraction (Lies et al., 2015; Drumm et al., 2020). The structure and/or quantity of alterations in ICCs can lead to changes in gastrointestinal function (Streutker et al., 2007). Various clinical gastrointestinal dysbiosis diseases, such as STC (Lee et al., 2005), functional dyspepsia (FD) (Zhang et al., 2018), and pseudointestinal obstruction (Foong et al., 2020), are associated with the ICC quantity and/or physiological abnormalities. A reduction in the number of ICCs, change in ICC volume, uneven distribution and functional abnormality of ICCs often play crucial roles in the pathological changes in STC and are considered key pathological factors of this condition.

Autophagy is a unique self-protective mechanism of the body and a lysosome-dependent degradation pathway in eukaryotic cells. It breaks down unnecessary or damaged cellular components, maintaining intracellular homeostasis (Levine and Kroemer, 2019). In this study, our findings revealed that after GA induction, the expression of the autophagy markers Beclin1 and LC3B increased in ICCs. Furthermore, the late-stage autophagy inhibitor BafA1, which blocks the fusion of autophagosomes with lysosomes, significantly reduced LC3B II levels in ICCs. Therefore, in this study, we explored the impact of increased autophagic activity in ICCs following GA treatment on their survival status. We observed that treatment with the autophagy inhibitor BafA1 could increase the survival rate and reduce the apoptosis rate of ICCs following GA treatment, suggesting a detrimental role of autophagy in ICC damage induced by GA. As an upstream regulatory factor of autophagy, the AMPK-mTOR signalling pathway is recognized as a key classical pathways regulating autophagy (Tong et al., 2020). AMP-activated protein kinase (AMPK), a widely distributed energy sensor in cells, has increasingly been recognized in recent years as a key regulatory protein in the autophagy process (An et al., 2014). It is a core component of cellular energy homeostasis, monitoring and maintaining the energy balance at both the cellular and organismal levels. AMPK activates autophagy through two main mechanisms: first, AMPK directly phosphorylates the Ser317, Ser555, and Ser777 sites of the ULK1 protein, promoting the formation of the ULK1-ATG13-FIP200 protein complex and thereby initiating autophagy; second, AMPK activation inhibits mTOR activity, reducing the mTOR-mediated phosphorylation of the Ser757 site on the ULK1 protein and relieving its inhibitory effect, thereby inducing autophagy (Mao and Klionsky, 2011; Iorio et al., 2021). Therefore, the interactions and balance between the AMPK, mTOR and ULK1 proteins are pivotal in regulating autoimmune diseases.

In this study, we observed the significant activation of the AMPK/mTOR/ULK1 pathway in ICCs following glutamate treatment. Treatment with the AMPK inhibitor dorsomorphin blocked autophagy activation in ICCs by inhibiting the activity of the AMPK/mTOR/ULK1 pathway, indicating that the AMPK/mTOR/ULK1 pathway mediates glutamate-induced autophagy in ICCs. After NAR treatment, the pT172-AMPK/AMPK and pS757-ULK1/ULK1 ratios increased, the expression of the autologous marker Beclin1 increased, and the pS2448-mTOR/mTOR ratio decreased.

Some studies have corroborated our experimental findings. Lin et al. discovered that the knockdown of AMPK led to a notable decrease in the level of Beclin-1, with an increase in phosphorylated ULK1 levels, whereas the phosphorylated mTOR levels decreased after treatment with BPA. The inhibition of autophagy using 3-methyladenine (3-MA) resulted in BPA-induced alterations in the protein levels of LC3B, Beclin1, LC3-II, LC3-I, and P62, along with modifications in the phosphorylation status of ULK1 and mTOR (Lin et al., 2021).

mTOR is a major regulator of autophagy. Under nutrient-rich conditions, mTOR directly phosphorylates ULK1 and Atg13, inhibiting the generation of the ULK1-ATG13-FIP200 complex and suppressing autophagy. Under stress conditions, mTOR no longer inhibits ULK1, inducing autophagy (Kim et al., 2011). However, autophagy is not constantly activated. Nazio and Cecconi proposed the concept of autophagic oscillation in 2017 as a method to prevent excessive autophagy activation. They suggested that during the initial induction of autophagy, ULK1 undergoes phosphorylation and ubiquitination for activation. Under prolonged stress conditions, neural precursor cell expressed developmentally downregulated gene 4-like (NEDD4L), increases the degradation of ULK1 via the proteasome, reducing its protein levels and inhibiting autophagy. Concurrently, the transcription and translation of the ULK1 mRNA are increased, followed by reinhibition by mTOR, restoring balance (Nazio and Cecconi, 2017). Therefore, the function of ULK1 is crucial for the initiation and balance of autophagy. ULK1 contains multiple functional phosphorylation sites, among which, activated AMPK directly phosphorylates residues Ser317, Ser555, and Ser777 of ULK1 under nutrient deprivation conditions (Egan et al., 2011). Conversely, the autophagy inhibitor mTOR can phosphorylate ULK1 at Ser757, disrupting the interaction between ULK1 and AMPK (Kim et al., 2011). Thus, as a downstream target of AMPK and mTOR, pS757-ULK1 is fundamental for enhancing the cellular autophagy mediated by the AMPK/mTOR/ULK1 pathway. However, current studies have focused predominantly on how the level of pS757-ULK1 regulates autophagic function, with little exploration into whether changes in its own levels are similarly regulated by intracellular autophagy. In this study, we treated groups of ICCs with or without BafA1. We found that BafA1 treatment reduced the levels of pS757-ULK1 in ICCs, with a more pronounced decrease observed in the GA + NAR group. Using the autophagy inhibitor BafA1 and Atg5 knockdown technology, we further treated groups of ICCs with or without BafA1 and Atg5 KD and observed the levels of pS757-ULK1 in ICCs. These findings confirmed that GA treatment reduced pS757-ULK1 levels in ICC and that NAR treatment significantly enhanced this reduction.

While this study primarily elucidates the role of NAR in regulating autophagy via the AMPK/mTOR/ULK1 pathway, its well-documented antioxidant properties are important to acknowledge. Previous studies have shown that NAR scavenges reactive oxygen species (ROS) and enhances cellular antioxidant defences (Salehi et al., 2019; Arafah et al., 2020), which could mitigate oxidative stress-induced damage in individuals with STC. Oxidative stress is a known trigger of autophagy dysregulation through pathways involving AMPK and mTOR (Scherz-Shouval and Elazar, 2007). The antioxidant activity of NAR may thus act in concert with its autophagy-modulating effects to restore ICC function. Although oxidative stress markers were not directly measured here, future studies could explore the interplay between the antioxidant mechanisms of NAR and AMPK/mTOR/ULK1 signalling in STC pathogenesis.

In recent years, as selective autophagy has garnered attention, researchers have discovered that many cellular components, including misfolded and modified proteins, can undergo selective autophagy. We considered pS757-ULK1 to be a protein that underwent mismodification when investigating the relationship between pS757-ULK1 and selective autophagy. Selective autophagy receptors can selectively bind to cargoes and, through their LIR sequence motif, indirectly bind to the cargo LC3, thereby mediating the autophagy of specific cargoes (Birgisdottir et al., 2013). Currently, widely studied selective autophagy receptors in the mammalian cytoplasm include p62, NBR1, NDP52 and OPTN (Turco et al., 2019; Vargas et al., 2019). Therefore, we employed siRNA technology to individually knock down the genes encoding these four autophagy receptors. By observing the accumulation of pS757-ULK1 in ICCs, we aimed to infer the relationship between pS757-ULK1 and autophagy receptors. We found that the autophagic degradation of pS757-ULK1 in ICCs induced by GA may be associated with the selective autophagy receptors NDP52 and OPTN rather than NBR1 and p62. Additionally, the inhibitory effect of NAR on autophagy may be associated with the selective autophagy receptors NDP52 and OPTN rather than NBR1 and p62. These results suggest that NAR decreases pS757-ULK1 levels by attenuating the interactions between pS757-ULK1 and NDP52 and OPTN, thereby further suppressing the autophagic activity of GA-induced ICCs. We generated a mouse model of hydrochloric acid-induced STC. The findings indicated that the faecal output, faecal water content, and intestinal transit rate in STC mice were significantly reduced, and colon tissue damage was also notable, which are consistent with the findings of Huang et al. (Huang et al., 2020), indicating pronounced colonic dysfunction. These results confirmed the successful construction of the STC mouse model. Faecal parameters, including faecal quantity, water content, weight, and the intestinal charcoal transit rate, are considered crucial factors for assessing constipation symptoms and the effectiveness of therapeutic interventions. In our study, no significant differences in body weight were observed between the experimental groups. However, the levels of faecal-related factors, including the faecal quantity, water content, and small intestinal transit rate, were markedly reduced in the mice that were administered Lop. These results validate the effective establishment of a constipation model in our study. However, these changes were dramatically reversed following NAR treatment. Yin et al. (Yang et al., 2008; Yin et al., 2018) reported that NAR has a laxative effect on Lop-induced constipation in mice. Notably, the effect of NAR on increasing the faecal water content may be associated with multiple mechanisms, including increases in the number of goblet cells and mucus secretion, the inhibition of autophagy in the colonic epithelium, and the regulation of secretory neurotransmitters (Karaki and Kuwahara, 2004). These coordinated effects collectively contribute to an improved faecal water content in the constipation model.

In mice with Lop-induced constipation, the serum levels of GAS, MTL, 5-HT, and SP were lower than those in control mice. However, the VIP and SS levels increased. The greatest peripheral neural system in an organism is the enteric nervous system (ENS), as previously noted. Its primary physiological role is to control digestion in the gastrointestinal tract (Holland et al., 2021). By releasing different neurotransmitters from neurons, the ENS controls the gut. Two categories of neurotransmitters have been established: excitatory and inhibitory. Excitatory neurotransmitters such as GAS, MTL, 5-HT, and SP can enhance intestinal smooth muscle contraction and promote gastrointestinal peristalsis; inhibitory neurotransmitters, such as VIP and SS, have inhibitory effects on gastrointestinal motility and reduce colonic peristalsis (Zhang et al., 2023). These findings indicate that the abnormal synthesis, storage, degradation, and release of neurotransmitters, or changes in receptor affinity and quantity, can lead to conduction disorders, resulting in gastrointestinal dysfunction (Chen et al., 2022). The results of our investigation revealed that the serum levels of different neurotransmitters in Lop + NAR group of mice were precisely the opposite of those in the Lop group and that the levels of neurotransmitters detected in the Lop group were reversed in a dose-dependent manner by NAR. These findings suggest that NAR reduces feelings of constipation by increasing the blood levels of GAS, MTL, 5-HT, and SP that are decreased by Lop, while lowering the levels of VIP and SS.

The colonic wall consists of several layers arranged from the innermost to the outermost layers as follows: the mucous layer (including the epithelial layer, lamina propria, and muscularis mucosae), submucosa, muscularis, and serosa. Goblet cells within the mucous layer synthesize, store and secrete mucus into the enteric cavity, with crucial functions in lubricating and protecting the colonic wall. Research indicates that during constipation, atrophy of the colonic mucosal glands occurs along with a reduction in the number of glandular acini and sparse distribution (Gao et al., 2022).

The neutral mucopolysaccharides represented by aminohexose and free hexosyl groups contained in the mucus secreted by goblet cells appear red when stained with PAS; the acidic mucopolysaccharides represented by sialic acid and sulfate esters at the molecular end appear blue–green when stained with AB; and the mixed mucus appears purple (Musch et al., 2013).

This study revealed that, after the intervention with three doses of NAR, varying degrees of improvement in the atrophy of the colonic mucosa and colonic glands were observed compared with the Lop group, as well as remarkable increases in mucosal thickness and mucus secretion. These results provide experimental data to elucidate the pharmacological mechanism by which NAR alleviates constipation from the perspective of colonic mucus secretion.

An increase in the number of goblet cells, an improvement in the crypt depth, and a significant trend towards thickened mucosal and muscular layers were observed. ANO1 is closely associated with the generation of pacing currents by the ICCs, which can regulate the excitability of intestinal smooth muscle. ICCs are widely distributed in various muscle layers of the digestive tract and act as pacemakers for gastrointestinal motility, participating in the development of various gastrointestinal motility disorders, including STC (Drumm et al., 2020). ANO1 is closely involved in the generation of pacing currents in the ICCs, which can regulate the excitability of intestinal smooth muscle (Sanders et al., 2023). c-Kit is considered a particular indicator of ICC and promotes the proliferation and growth of ICCs by binding to the receptor SCF (Al-Ahmadi et al., 2023). In our study, NAR notably increased the expression of ANO1 and c-Kit in the colonic tissues of mice with Lop-induced constipating. These findings indicated that NAR increases the amount of ICCs in mice with Lop-induced constipation.

Pharmacokinetic studies indicate that orally administered NAR has low systemic bioavailability (5.8%–15% in humans) due to extensive phase II metabolism (Deleu et al., 2001; Kanaze et al., 2007). Notably, intestinal perfusion studies revealed that NAR is absorbed mainly in the colon, accounting for 68% of the perfused amount (Xu et al., 2009). Its conjugated metabolites (glucuronides/sulfates) are likely to accumulate in colonic tissues via enterohepatic recirculation. This pattern mirrors baicalin metabolism, where colonic microbiota-mediated hydrolysis converts the poorly absorbed glycoside into bioactive baicalein (Noh et al., 2016). While our current methodology lacks a direct quantification of NAR metabolites in serum or intestinal tissues via mass spectrometry, future studies will incorporate pharmacokinetic profiling and a tissue-specific metabolite analysis to validate these distribution patterns.

## 5 Conclusion

In summary, our study elucidated how NAR promoted colonic motility by regulating the AMPK/mTOR/ULK1 signalling pathway. Specifically, NAR inhibited STC development by increasing the expression of the proteins ANO1 and c-Kit, attenuating the autophagic response and regulating the AMPK/mTOR/ULK1 pathway.

This research highlights how NAR affects the AMPK/mTOR/ULK1 pathway to suppress autophagy and ameliorate colonic motility disorders triggered by STC. In summary, these results highlight the potential of NAR as a modulator of the AMPK/mTOR/ULK1 pathway for the potentially effective prevention and treatment of STC, providing an experimental basis for its future clinical application. However, extensive safety and efficacy studies are needed before its use in the clinic. Furthermore, the specific mechanisms of NAR and STC need further investigation in future research, particularly in the context of selective genes that warrant further study.

## Data Availability

The original contributions presented in the study are included in the article/Supplementary Material, further inquiries can be directed to the corresponding author.

## Supplementary material

Supplementary Figure S1 Results of immunofluorescence identification of rat ICCs. DAPI was used for nuclear staining (blue), and the target fluorescent marker (green) showed the characteristic expression of ICC. The cell morphology and fluorescence distribution were observed at magnifications of 100× and 200×.

Supplementary Figure S2 FIGURE S2 Relative mRNA quantities of Atg5, NDP52, OPTN, NBR1, and p62 in ICCs following transfection with si - NC, siRNA - 1, siRNA - 2, and siRNA – 3. Data are presented as the means ± SD. ***P < 0.001 vs. Control group. NDP52: Nuclear dot protein 52 kDa; OPTN: Optineurin; NBR1: Neighbor of BRCA1 gene 1 protein.
