# Supplementary figures and images for: Naringenin attenuates slow-transit constipation by regulating the AMPK/mTOR/ULK1 signalling pathway: in vivo and in vitro studies (part 2 of 2)
Source: Front Pharmacol. 2025 Jun 17;16:1550458. doi: 10.3389/fphar.2025.1550458 (PMC12209368; doi:10.3389/fphar.2025.1550458)

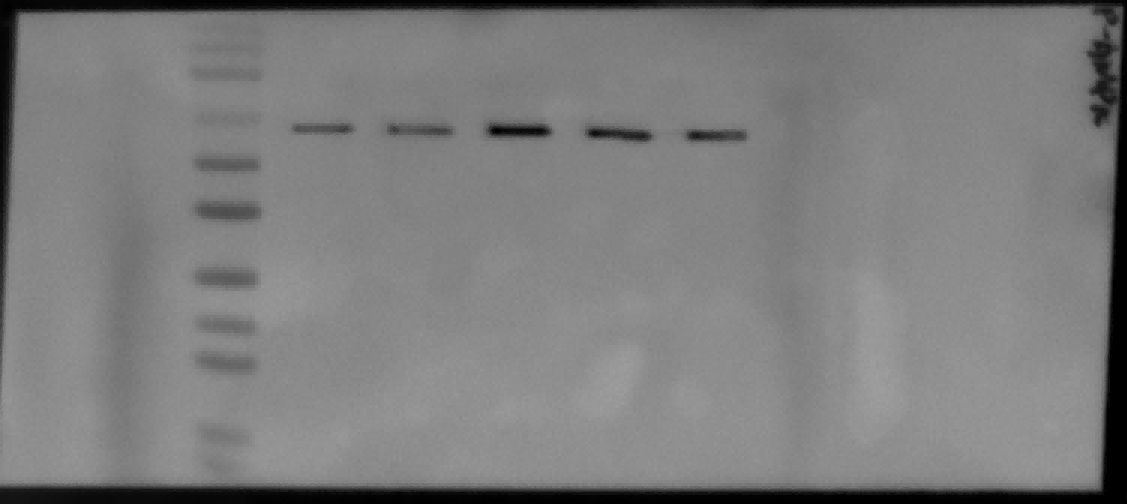

Supplement: Supplementary file 1 [file DataSheet1.zip › Data/Figure.3/Figure/Western Blot/Figure.3 2/P-AMPK 1.png]

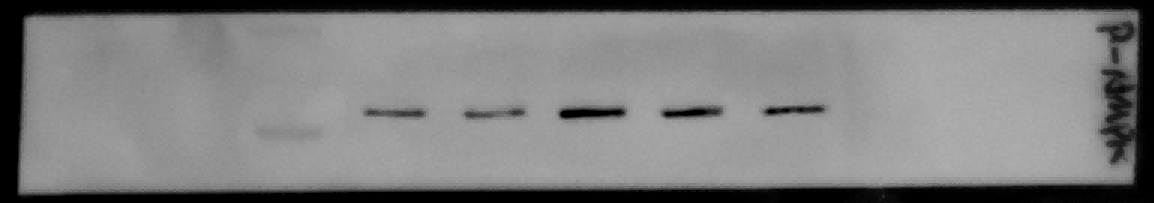

Supplement: Supplementary file 1 [file DataSheet1.zip › Data/Figure.3/Figure/Western Blot/Figure.3 2/P-AMPK 2.png]

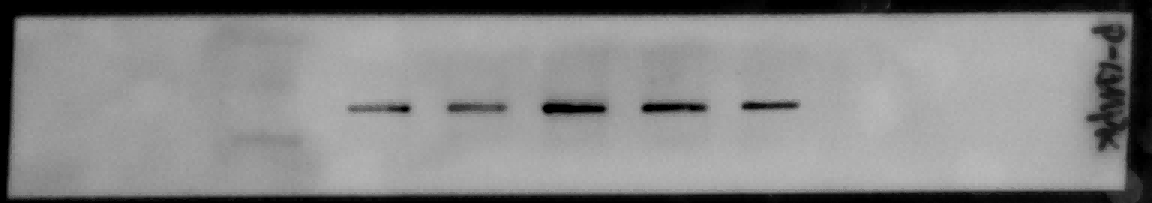

Supplement: Supplementary file 1 [file DataSheet1.zip › Data/Figure.3/Figure/Western Blot/Figure.3 2/P-AMPK 3.png]

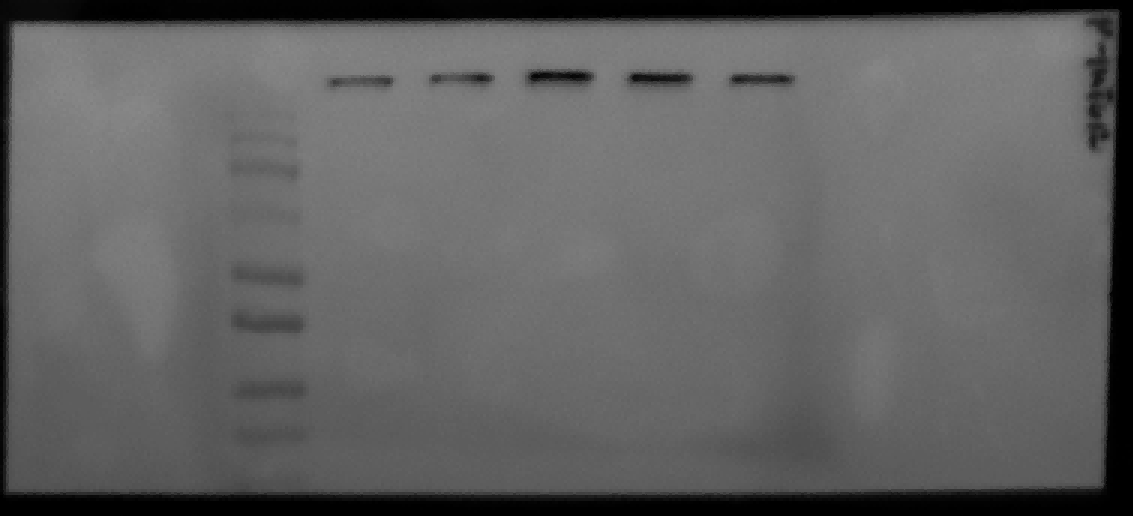

Supplement: Supplementary file 1 [file DataSheet1.zip › Data/Figure.3/Figure/Western Blot/Figure.3 2/P-MTOR 1.png]

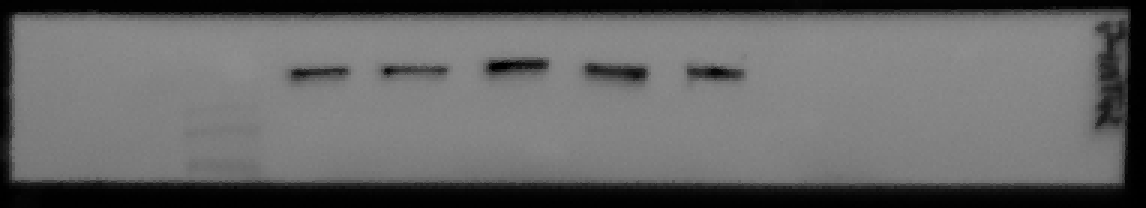

Supplement: Supplementary file 1 [file DataSheet1.zip › Data/Figure.3/Figure/Western Blot/Figure.3 2/P-MTOR 2.png]

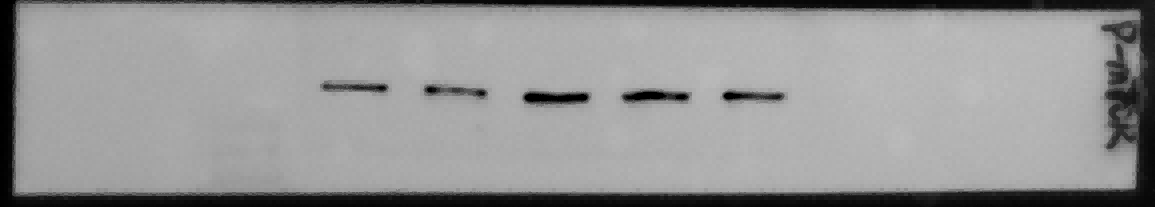

Supplement: Supplementary file 1 [file DataSheet1.zip › Data/Figure.3/Figure/Western Blot/Figure.3 2/P-MTOR 3.png]

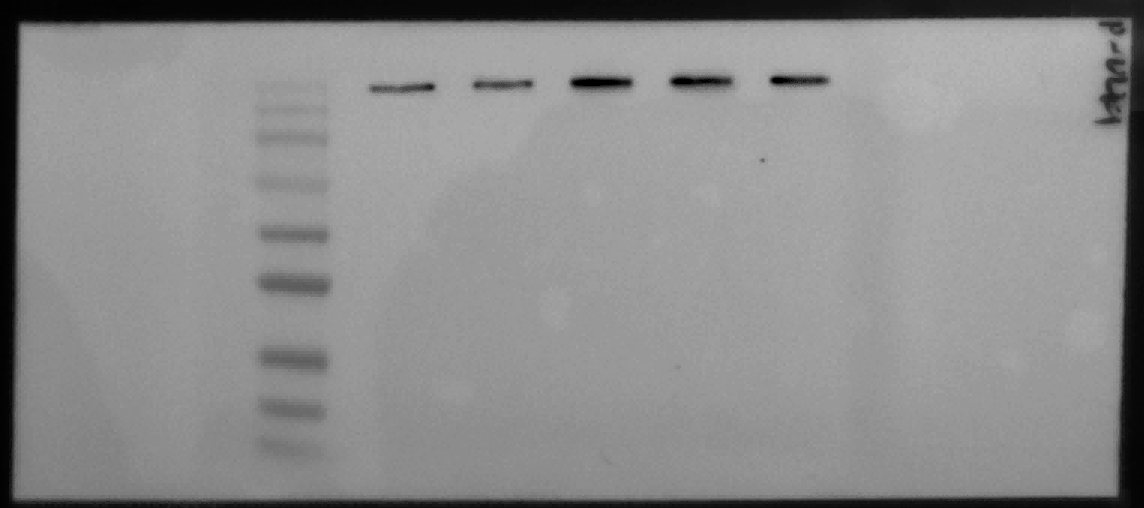

Supplement: Supplementary file 1 [file DataSheet1.zip › Data/Figure.3/Figure/Western Blot/Figure.3 2/P-ULK1 1.png]

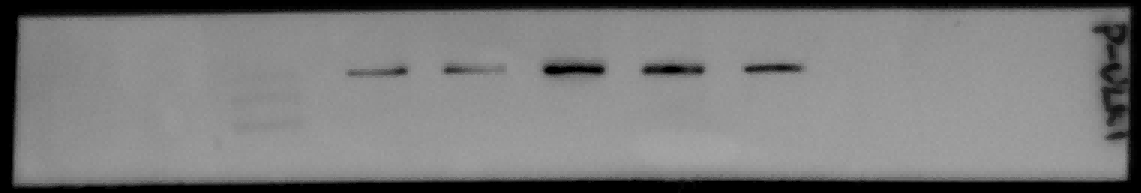

Supplement: Supplementary file 1 [file DataSheet1.zip › Data/Figure.3/Figure/Western Blot/Figure.3 2/P-ULK1 2.png]

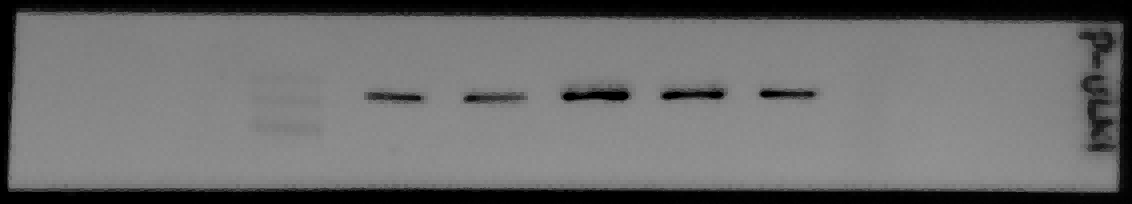

Supplement: Supplementary file 1 [file DataSheet1.zip › Data/Figure.3/Figure/Western Blot/Figure.3 2/P-ULK1 3.png]

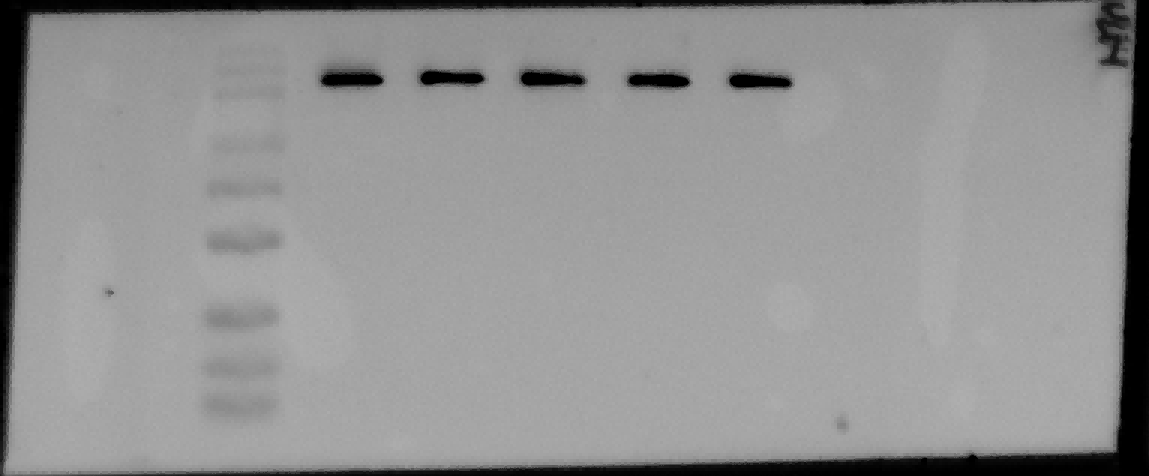

Supplement: Supplementary file 1 [file DataSheet1.zip › Data/Figure.3/Figure/Western Blot/Figure.3 2/ULK1 1.png]

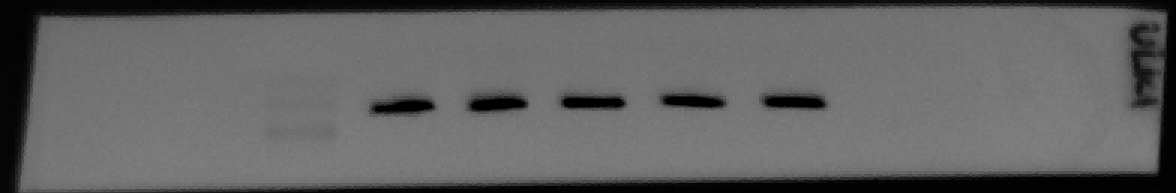

Supplement: Supplementary file 1 [file DataSheet1.zip › Data/Figure.3/Figure/Western Blot/Figure.3 2/ULK1 2.png]

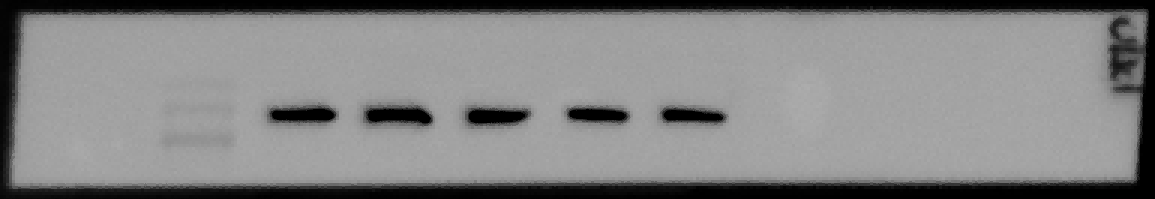

Supplement: Supplementary file 1 [file DataSheet1.zip › Data/Figure.3/Figure/Western Blot/Figure.3 2/ULK1 3.png]

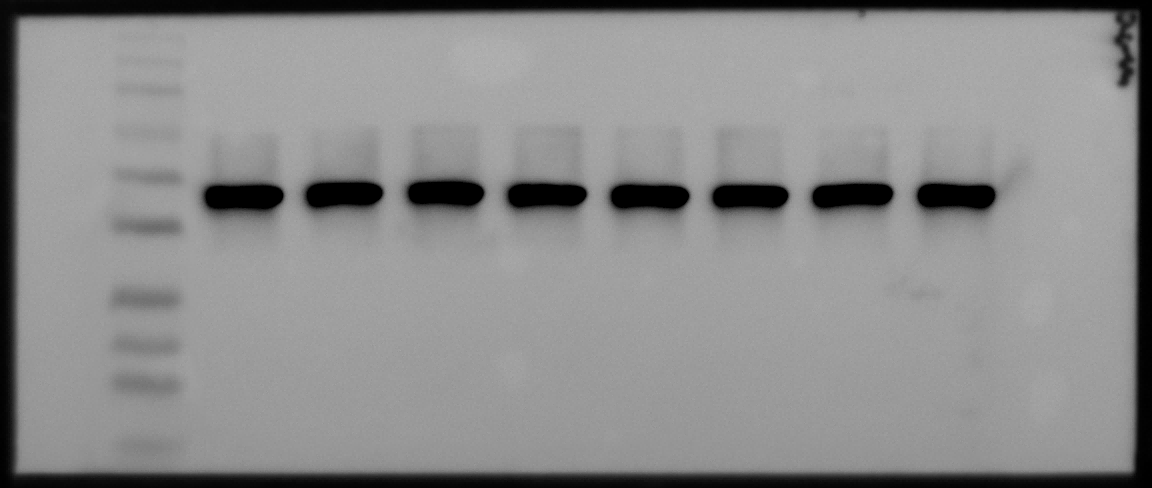

Supplement: Supplementary file 1 [file DataSheet1.zip › Data/Figure.4/Figure/Western Blot/Figure.4 1/actin 1.png]

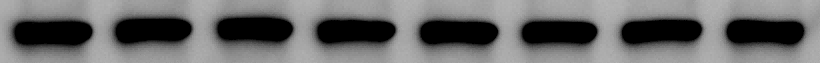

Supplement: Supplementary file 1 [file DataSheet1.zip › Data/Figure.4/Figure/Western Blot/Figure.4 1/actin 1灰.png]

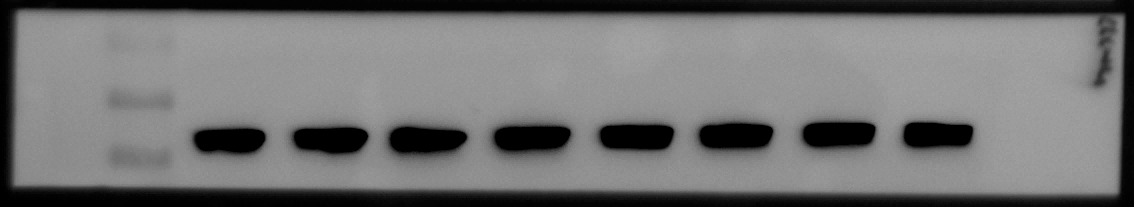

Supplement: Supplementary file 1 [file DataSheet1.zip › Data/Figure.4/Figure/Western Blot/Figure.4 1/actin 2.png]

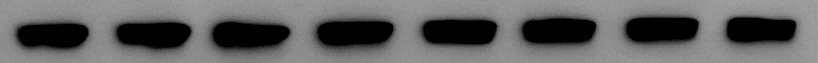

Supplement: Supplementary file 1 [file DataSheet1.zip › Data/Figure.4/Figure/Western Blot/Figure.4 1/actin 2灰.png]

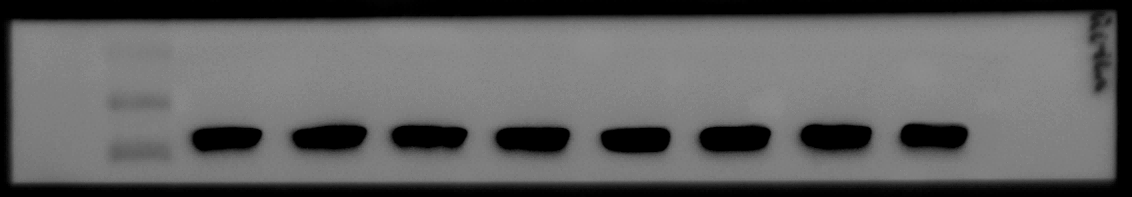

Supplement: Supplementary file 1 [file DataSheet1.zip › Data/Figure.4/Figure/Western Blot/Figure.4 1/actin 3.png]

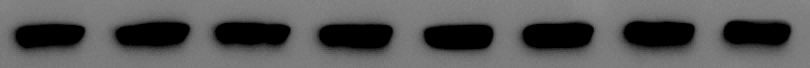

Supplement: Supplementary file 1 [file DataSheet1.zip › Data/Figure.4/Figure/Western Blot/Figure.4 1/actin 3灰.png]

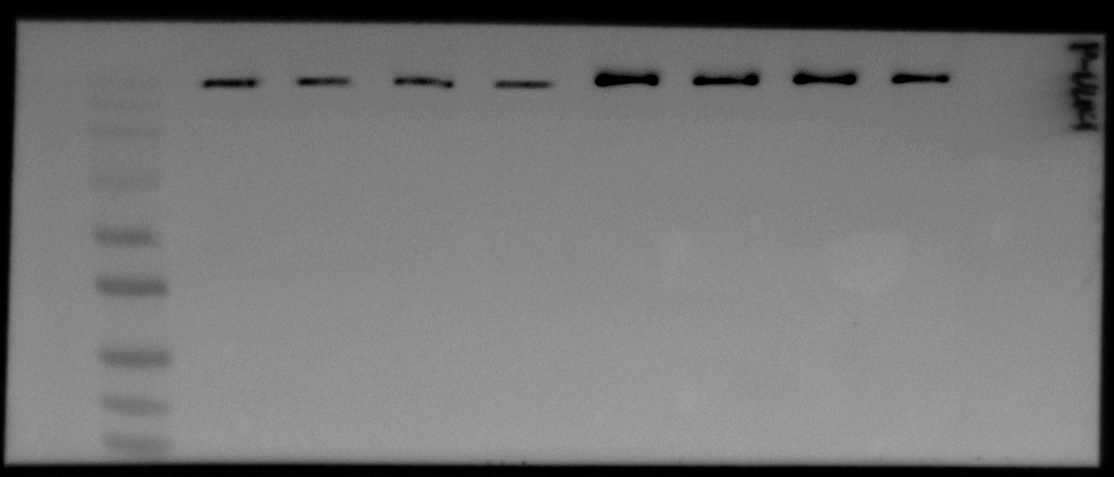

Supplement: Supplementary file 1 [file DataSheet1.zip › Data/Figure.4/Figure/Western Blot/Figure.4 1/P-ULK1 1.png]

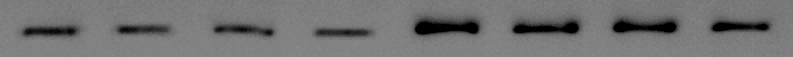

Supplement: Supplementary file 1 [file DataSheet1.zip › Data/Figure.4/Figure/Western Blot/Figure.4 1/P-ULK1 1灰.png]

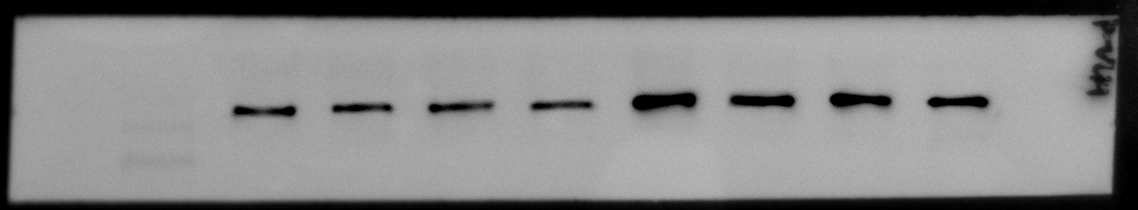

Supplement: Supplementary file 1 [file DataSheet1.zip › Data/Figure.4/Figure/Western Blot/Figure.4 1/P-ULK1 2.png]

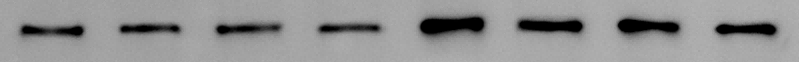

Supplement: Supplementary file 1 [file DataSheet1.zip › Data/Figure.4/Figure/Western Blot/Figure.4 1/P-ULK1 2灰.png]

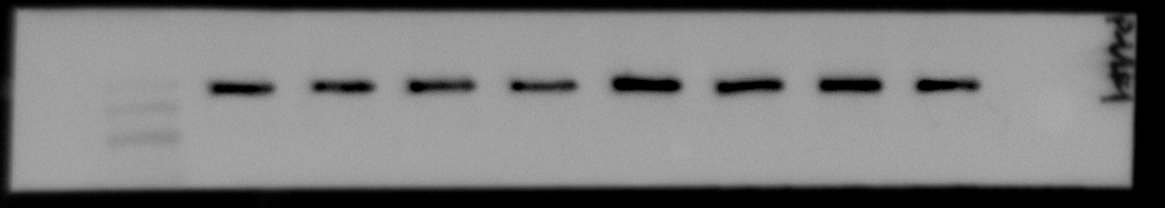

Supplement: Supplementary file 1 [file DataSheet1.zip › Data/Figure.4/Figure/Western Blot/Figure.4 1/P-ULK1 3.png]

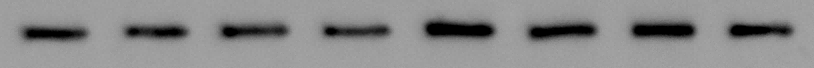

Supplement: Supplementary file 1 [file DataSheet1.zip › Data/Figure.4/Figure/Western Blot/Figure.4 1/P-ULK1 3灰.png]

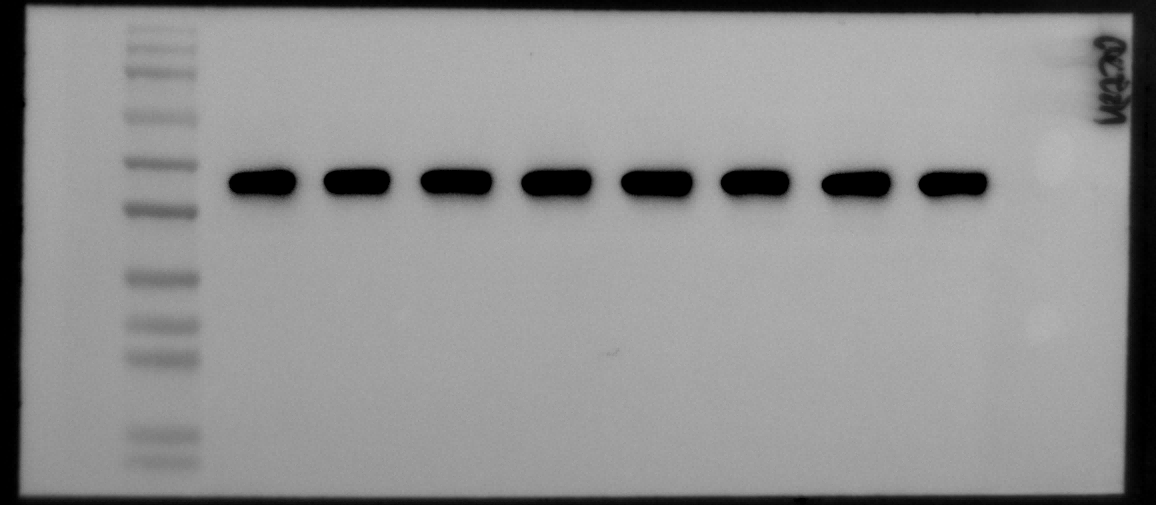

Supplement: Supplementary file 1 [file DataSheet1.zip › Data/Figure.4/Figure/Western Blot/Figure.4 2/actin 1.png]

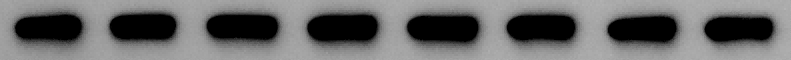

Supplement: Supplementary file 1 [file DataSheet1.zip › Data/Figure.4/Figure/Western Blot/Figure.4 2/actin 1灰.png]

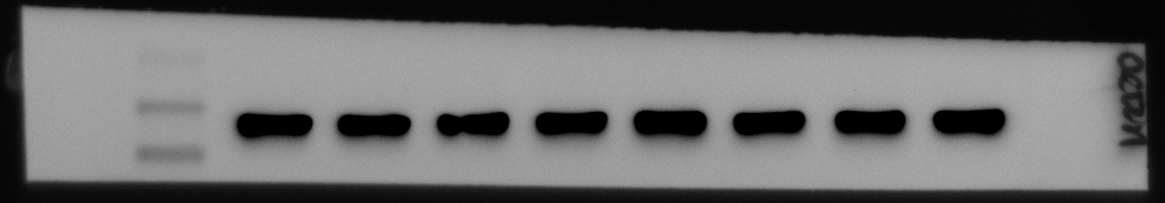

Supplement: Supplementary file 1 [file DataSheet1.zip › Data/Figure.4/Figure/Western Blot/Figure.4 2/actin 2.png]

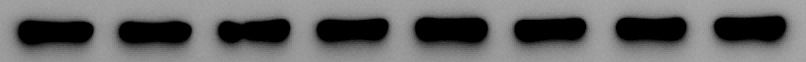

Supplement: Supplementary file 1 [file DataSheet1.zip › Data/Figure.4/Figure/Western Blot/Figure.4 2/actin 2灰.png]

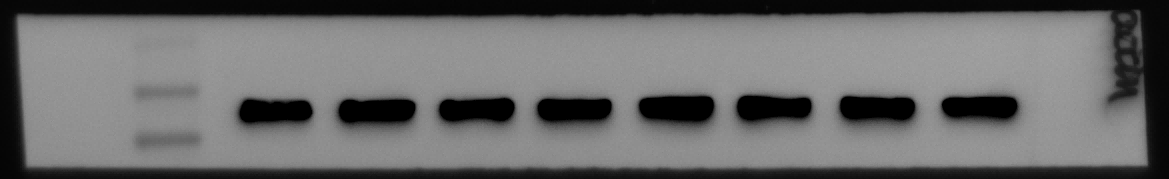

Supplement: Supplementary file 1 [file DataSheet1.zip › Data/Figure.4/Figure/Western Blot/Figure.4 2/actin 3.png]

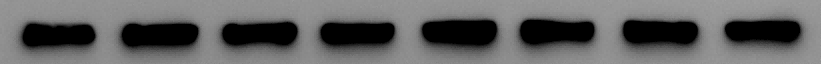

Supplement: Supplementary file 1 [file DataSheet1.zip › Data/Figure.4/Figure/Western Blot/Figure.4 2/actin 3灰.png]

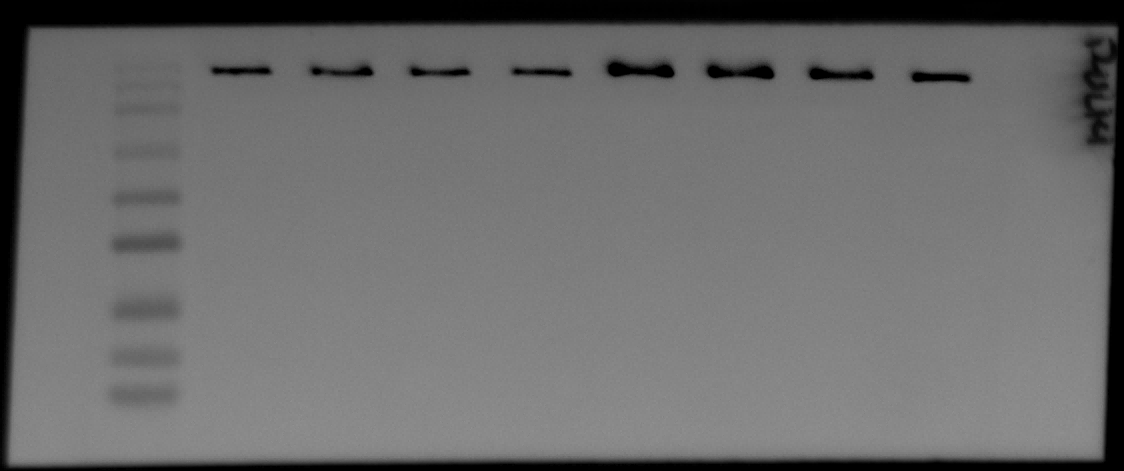

Supplement: Supplementary file 1 [file DataSheet1.zip › Data/Figure.4/Figure/Western Blot/Figure.4 2/p-ULK1 1.png]

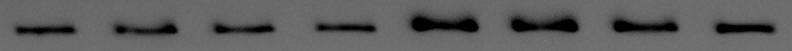

Supplement: Supplementary file 1 [file DataSheet1.zip › Data/Figure.4/Figure/Western Blot/Figure.4 2/p-ULK1 1灰.png]

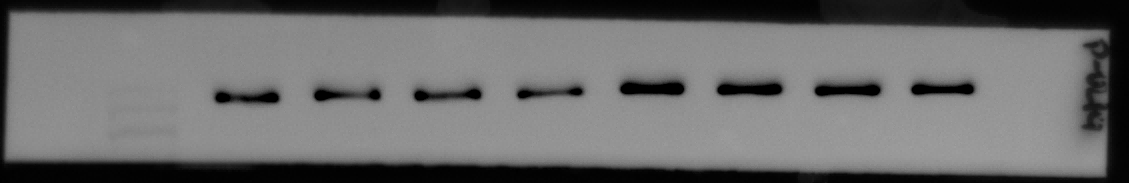

Supplement: Supplementary file 1 [file DataSheet1.zip › Data/Figure.4/Figure/Western Blot/Figure.4 2/p-ULK1 2.png]

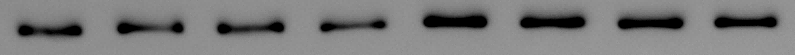

Supplement: Supplementary file 1 [file DataSheet1.zip › Data/Figure.4/Figure/Western Blot/Figure.4 2/p-ULK1 2灰.png]

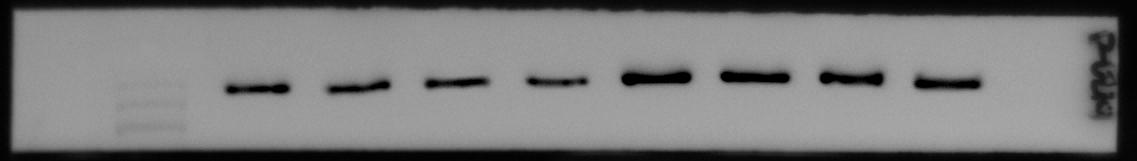

Supplement: Supplementary file 1 [file DataSheet1.zip › Data/Figure.4/Figure/Western Blot/Figure.4 2/p-ULK1 3.png]

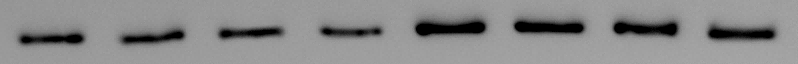

Supplement: Supplementary file 1 [file DataSheet1.zip › Data/Figure.4/Figure/Western Blot/Figure.4 2/p-ULK1 3灰.png]

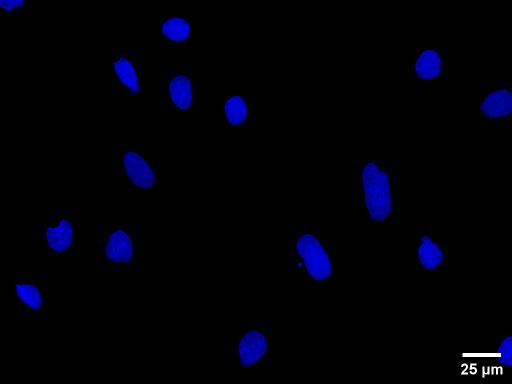

Supplement: Supplementary file 1 [file DataSheet1.zip › Data/Figure.5/Figure/IF/ULK1+NDP52/ULK1+NDP52/1GA 1-400 DAPI.png]

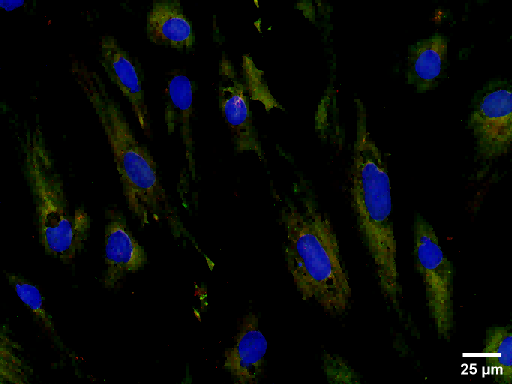

Supplement: Supplementary file 1 [file DataSheet1.zip › Data/Figure.5/Figure/IF/ULK1+NDP52/ULK1+NDP52/1GA 1-400 MERGE.png]

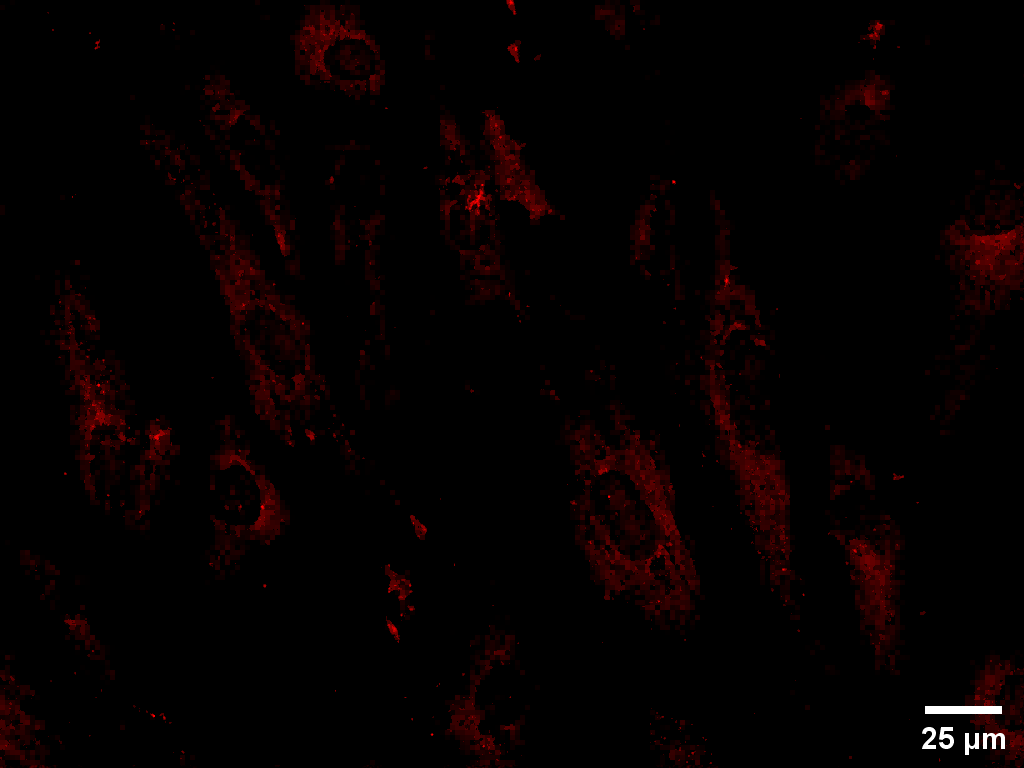

Supplement: Supplementary file 1 [file DataSheet1.zip › Data/Figure.5/Figure/IF/ULK1+NDP52/ULK1+NDP52/1GA 1-400 NDP52.png]

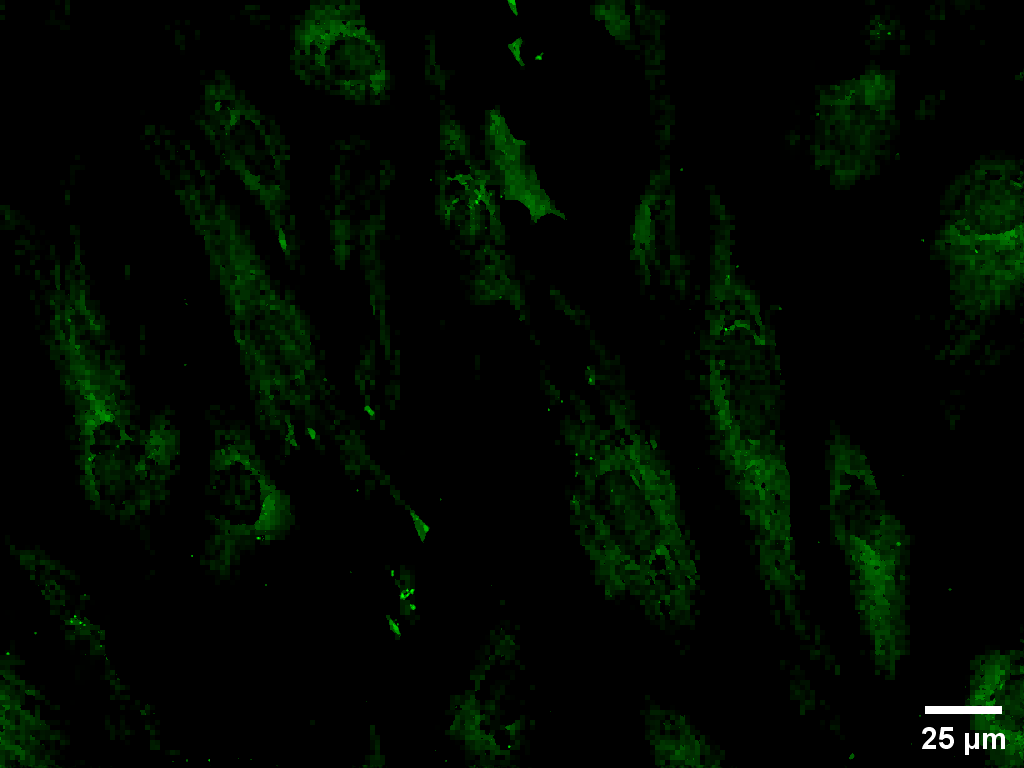

Supplement: Supplementary file 1 [file DataSheet1.zip › Data/Figure.5/Figure/IF/ULK1+NDP52/ULK1+NDP52/1GA 1-400 p-ULK1.png]

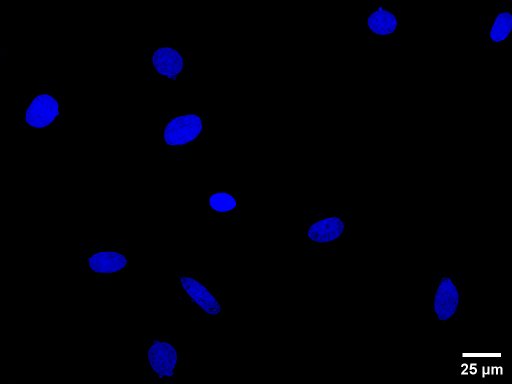

Supplement: Supplementary file 1 [file DataSheet1.zip › Data/Figure.5/Figure/IF/ULK1+NDP52/ULK1+NDP52/1GA 2-400 DAPI.png]

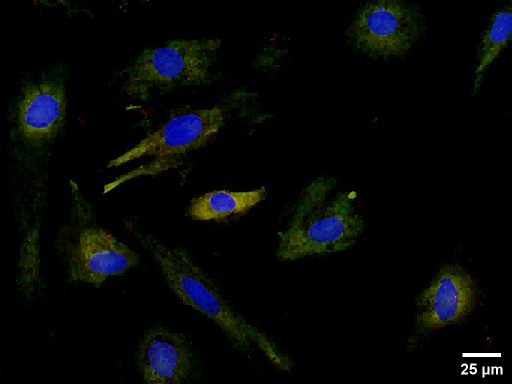

Supplement: Supplementary file 1 [file DataSheet1.zip › Data/Figure.5/Figure/IF/ULK1+NDP52/ULK1+NDP52/1GA 2-400 merge.png]

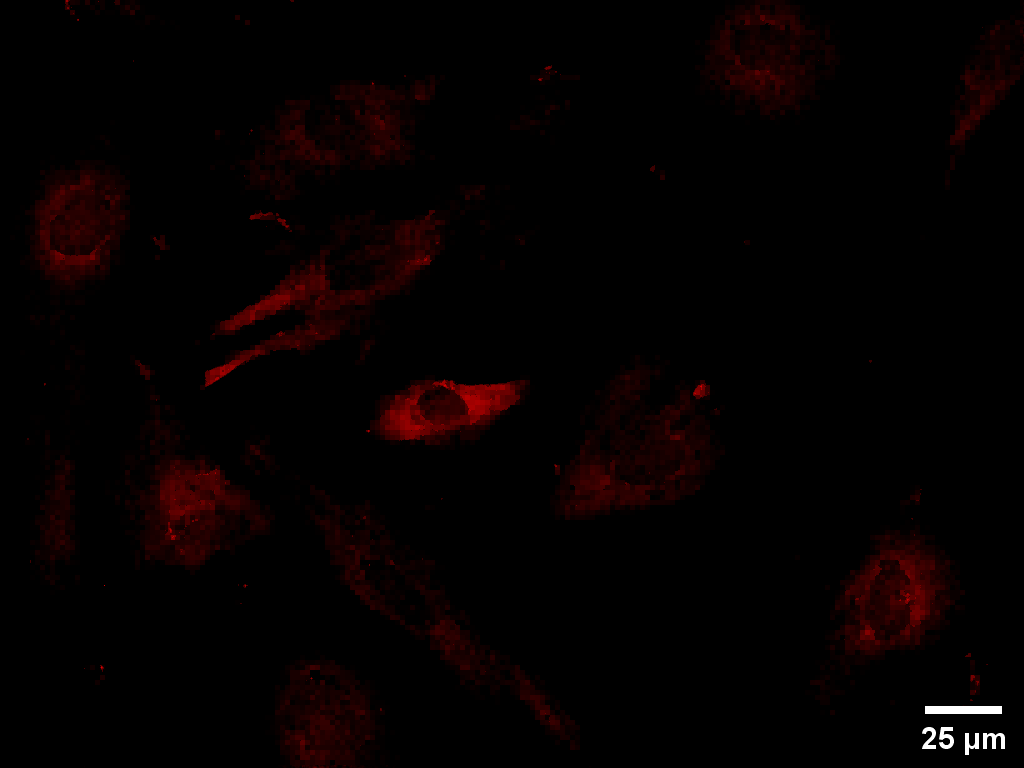

Supplement: Supplementary file 1 [file DataSheet1.zip › Data/Figure.5/Figure/IF/ULK1+NDP52/ULK1+NDP52/1GA 2-400 NDP52.png]

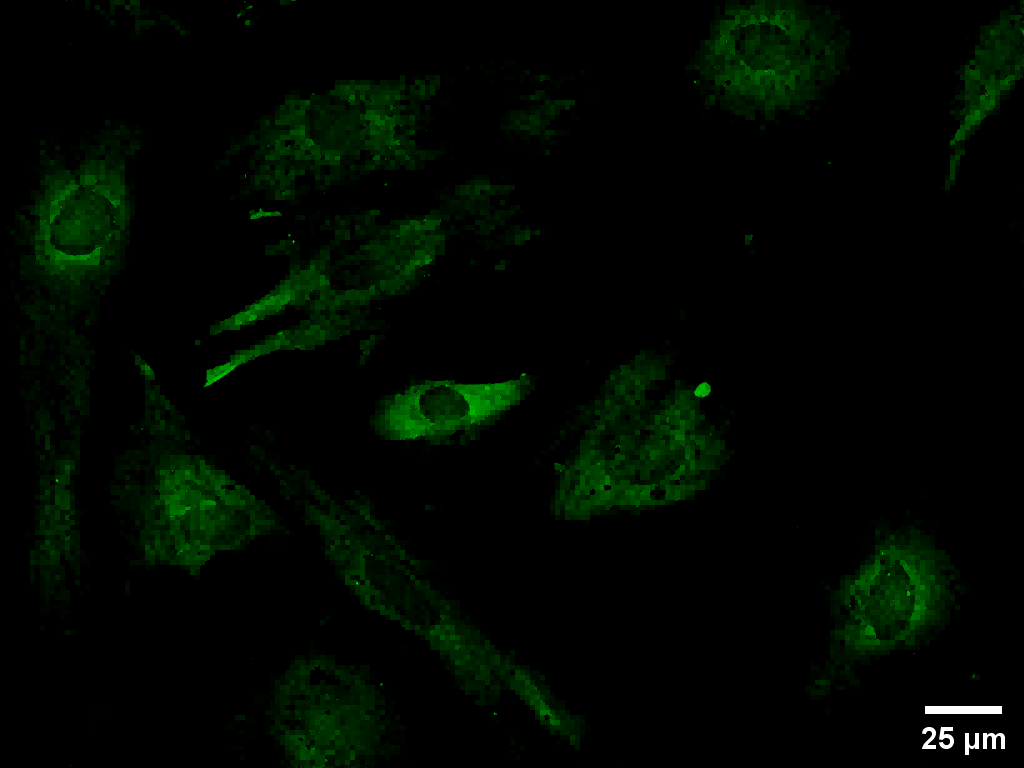

Supplement: Supplementary file 1 [file DataSheet1.zip › Data/Figure.5/Figure/IF/ULK1+NDP52/ULK1+NDP52/1GA 2-400 p-ULK1.png]

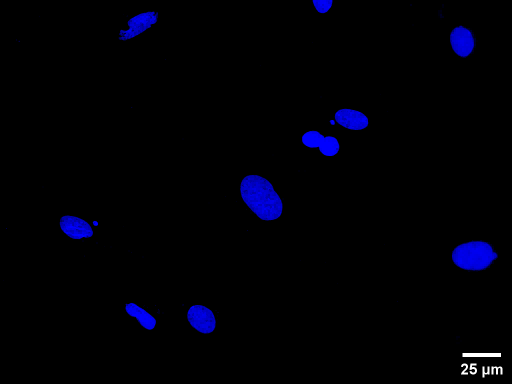

Supplement: Supplementary file 1 [file DataSheet1.zip › Data/Figure.5/Figure/IF/ULK1+NDP52/ULK1+NDP52/1GA 3-400 DAPI.png]

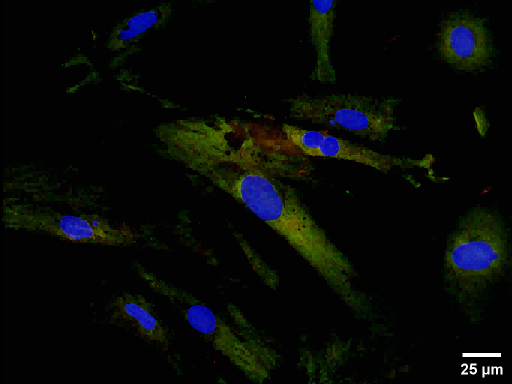

Supplement: Supplementary file 1 [file DataSheet1.zip › Data/Figure.5/Figure/IF/ULK1+NDP52/ULK1+NDP52/1GA 3-400 merge.png]

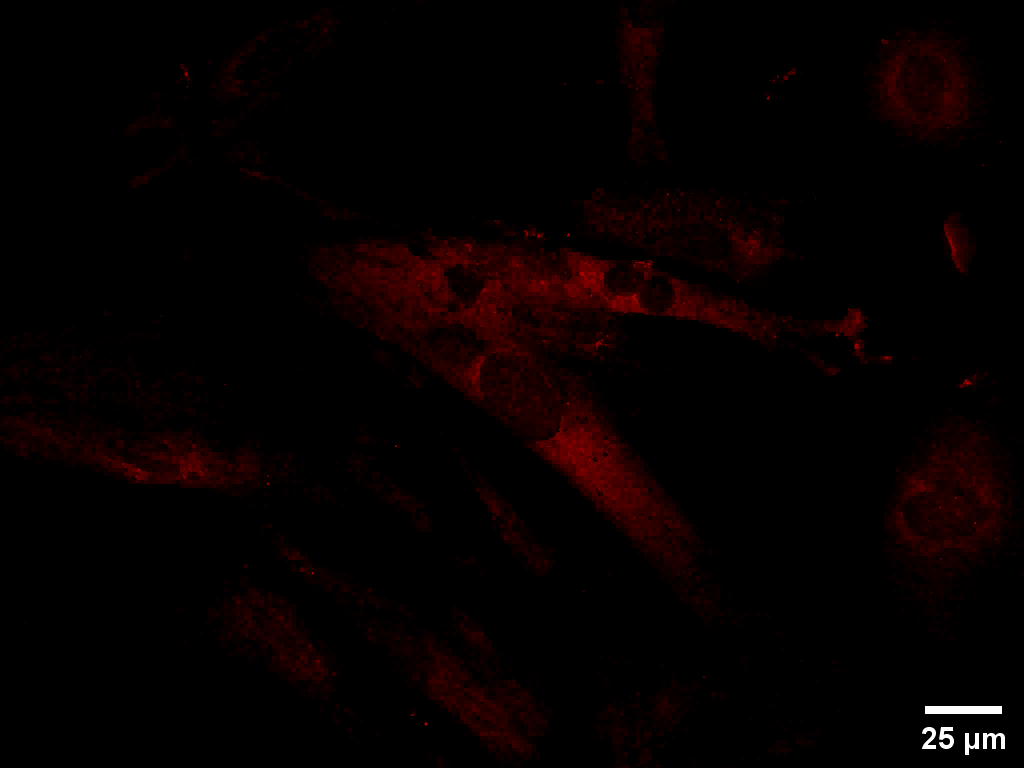

Supplement: Supplementary file 1 [file DataSheet1.zip › Data/Figure.5/Figure/IF/ULK1+NDP52/ULK1+NDP52/1GA 3-400 NDP52.png]

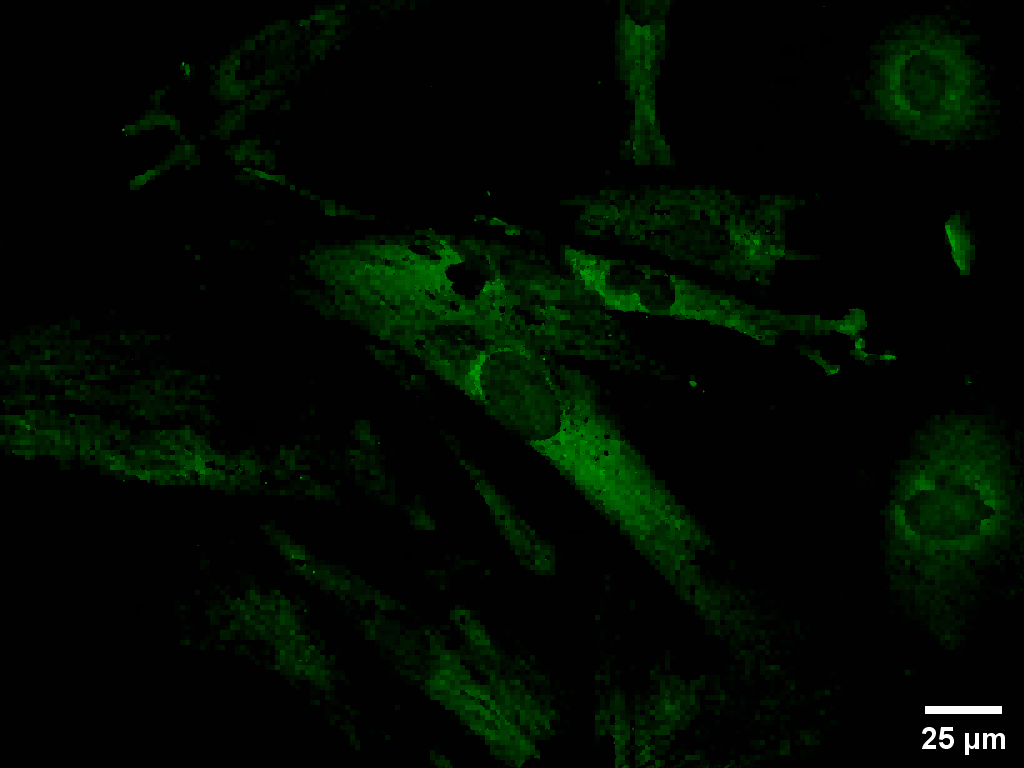

Supplement: Supplementary file 1 [file DataSheet1.zip › Data/Figure.5/Figure/IF/ULK1+NDP52/ULK1+NDP52/1GA 3-400 p-ULK1.png]

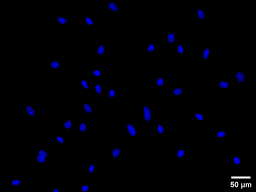

Supplement: Supplementary file 1 [file DataSheet1.zip › Data/Figure.5/Figure/IF/ULK1+NDP52/ULK1+NDP52/1GA-200 DAPI.png]

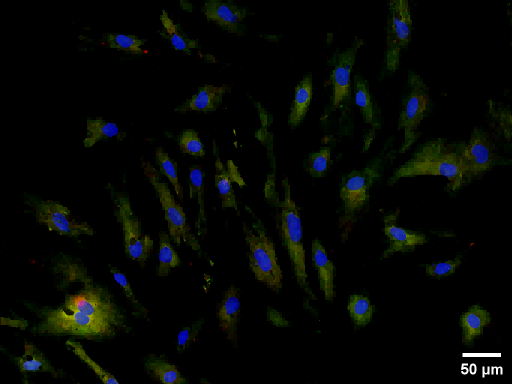

Supplement: Supplementary file 1 [file DataSheet1.zip › Data/Figure.5/Figure/IF/ULK1+NDP52/ULK1+NDP52/1GA-200 merge.png]

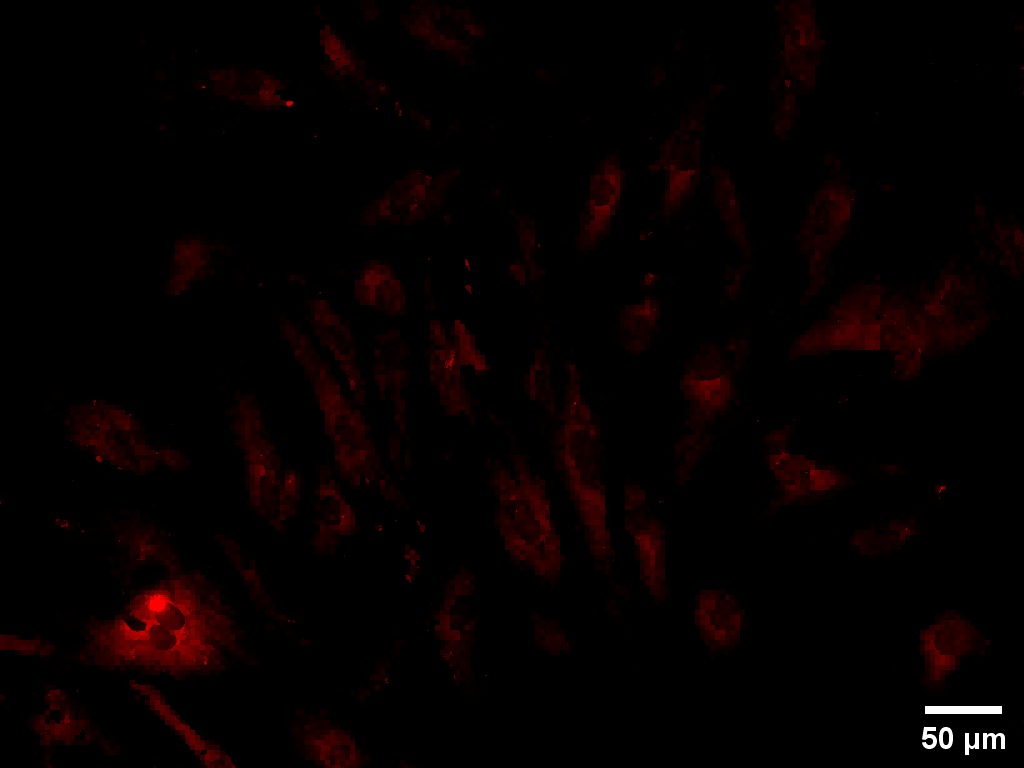

Supplement: Supplementary file 1 [file DataSheet1.zip › Data/Figure.5/Figure/IF/ULK1+NDP52/ULK1+NDP52/1GA-200 NDP52.png]

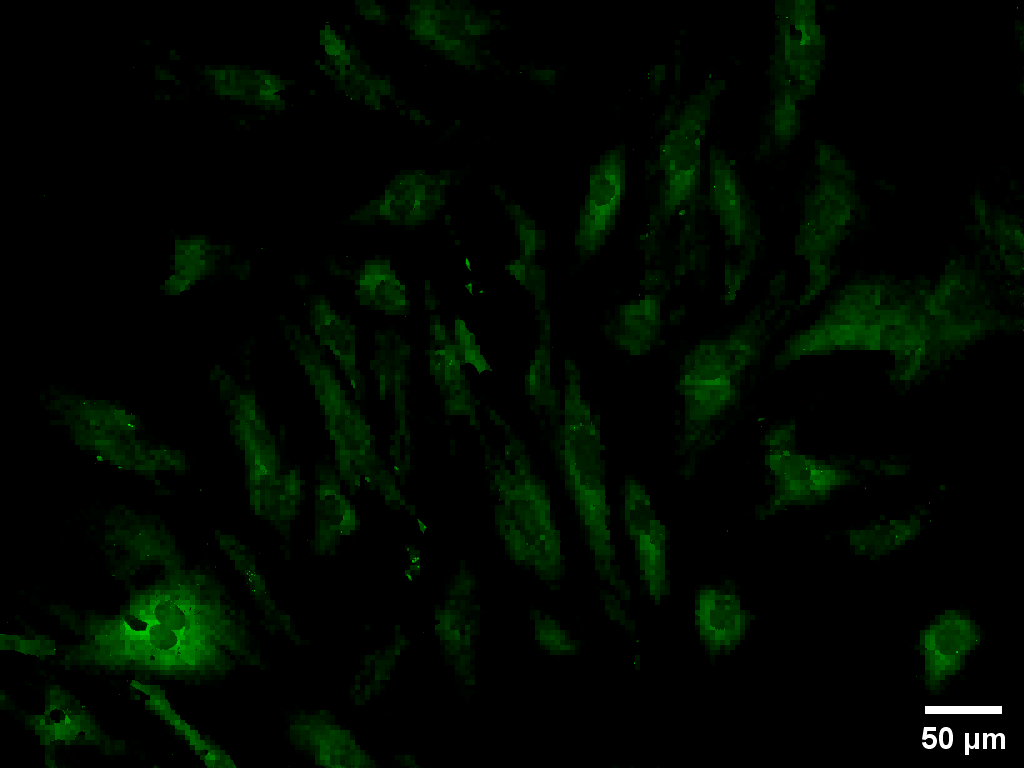

Supplement: Supplementary file 1 [file DataSheet1.zip › Data/Figure.5/Figure/IF/ULK1+NDP52/ULK1+NDP52/1GA-200 p-ULK1.png]

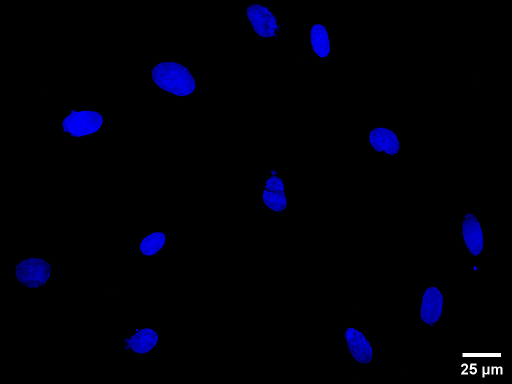

Supplement: Supplementary file 1 [file DataSheet1.zip › Data/Figure.5/Figure/IF/ULK1+NDP52/ULK1+NDP52/2GA-NDP52 1-400 DAPI.png]

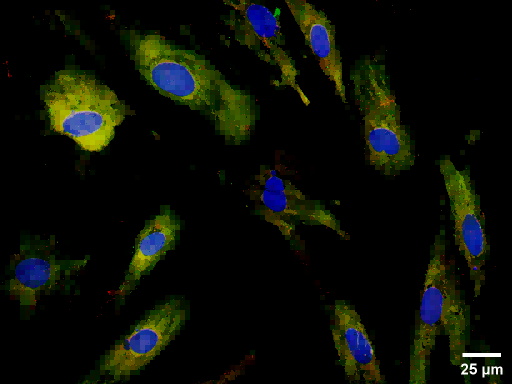

Supplement: Supplementary file 1 [file DataSheet1.zip › Data/Figure.5/Figure/IF/ULK1+NDP52/ULK1+NDP52/2GA-NDP52 1-400 merge.png]

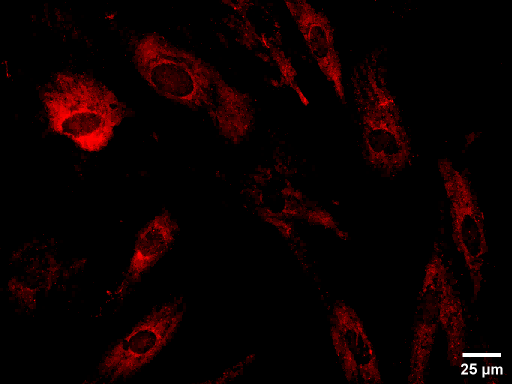

Supplement: Supplementary file 1 [file DataSheet1.zip › Data/Figure.5/Figure/IF/ULK1+NDP52/ULK1+NDP52/2GA-NDP52 1-400 NDP52.png]

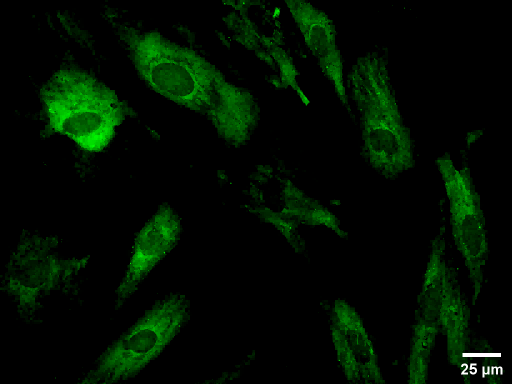

Supplement: Supplementary file 1 [file DataSheet1.zip › Data/Figure.5/Figure/IF/ULK1+NDP52/ULK1+NDP52/2GA-NDP52 1-400 p-ULK1.png]

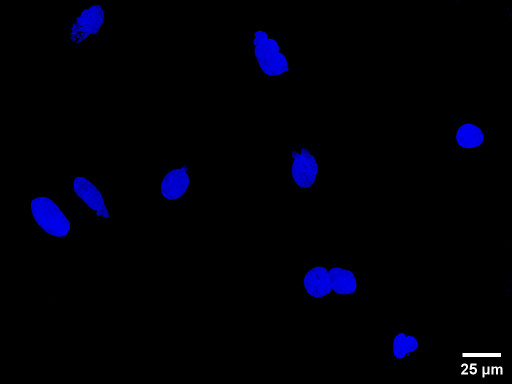

Supplement: Supplementary file 1 [file DataSheet1.zip › Data/Figure.5/Figure/IF/ULK1+NDP52/ULK1+NDP52/2GA-NDP52 2-400 DAPI.png]

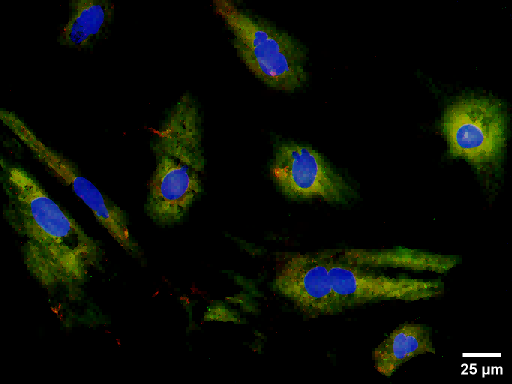

Supplement: Supplementary file 1 [file DataSheet1.zip › Data/Figure.5/Figure/IF/ULK1+NDP52/ULK1+NDP52/2GA-NDP52 2-400 merge.png]

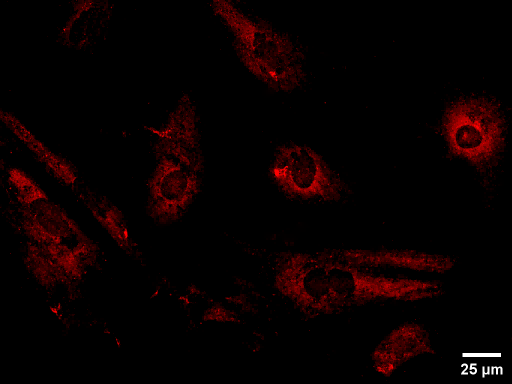

Supplement: Supplementary file 1 [file DataSheet1.zip › Data/Figure.5/Figure/IF/ULK1+NDP52/ULK1+NDP52/2GA-NDP52 2-400 NDP52.png]

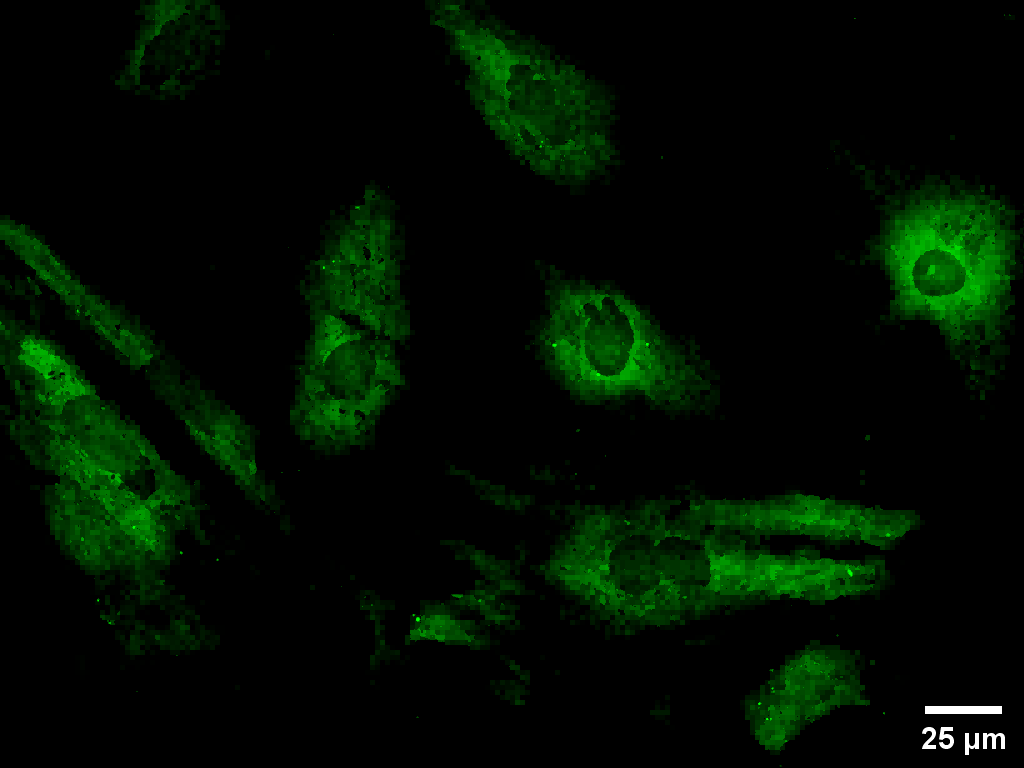

Supplement: Supplementary file 1 [file DataSheet1.zip › Data/Figure.5/Figure/IF/ULK1+NDP52/ULK1+NDP52/2GA-NDP52 2-400 p-ULK1.png]

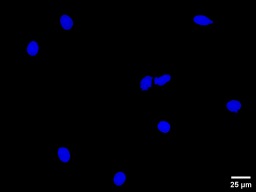

Supplement: Supplementary file 1 [file DataSheet1.zip › Data/Figure.5/Figure/IF/ULK1+NDP52/ULK1+NDP52/2GA-NDP52 3-400 DAPI.png]

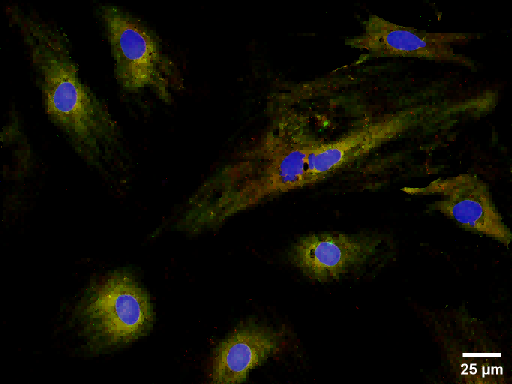

Supplement: Supplementary file 1 [file DataSheet1.zip › Data/Figure.5/Figure/IF/ULK1+NDP52/ULK1+NDP52/2GA-NDP52 3-400 merge.png]

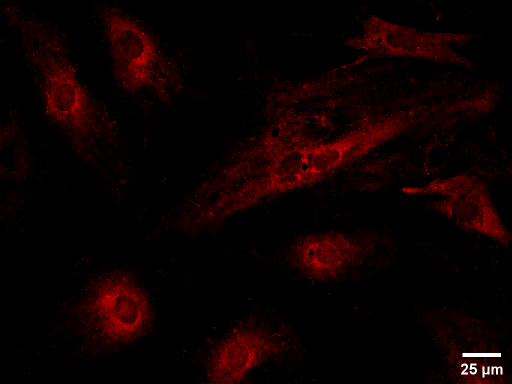

Supplement: Supplementary file 1 [file DataSheet1.zip › Data/Figure.5/Figure/IF/ULK1+NDP52/ULK1+NDP52/2GA-NDP52 3-400 NDP52.png]

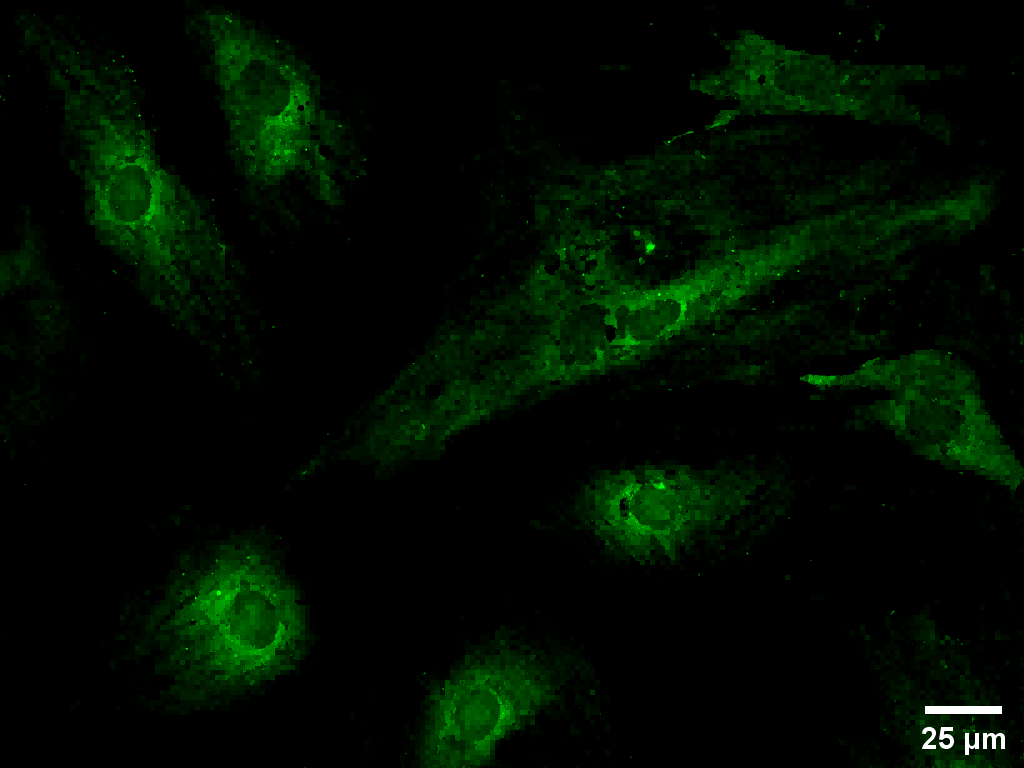

Supplement: Supplementary file 1 [file DataSheet1.zip › Data/Figure.5/Figure/IF/ULK1+NDP52/ULK1+NDP52/2GA-NDP52 3-400 p-ULK1.png]

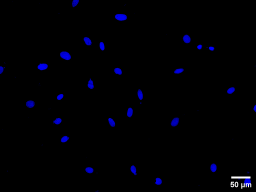

Supplement: Supplementary file 1 [file DataSheet1.zip › Data/Figure.5/Figure/IF/ULK1+NDP52/ULK1+NDP52/2GA-NDP52-200 DAPI.png]

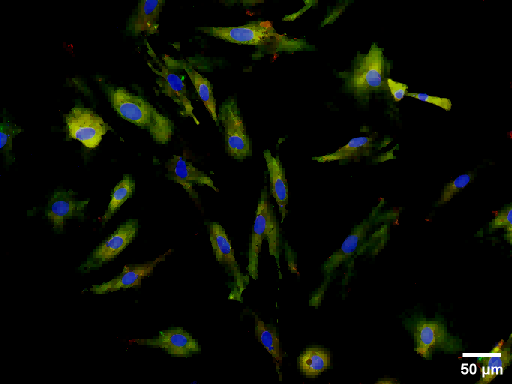

Supplement: Supplementary file 1 [file DataSheet1.zip › Data/Figure.5/Figure/IF/ULK1+NDP52/ULK1+NDP52/2GA-NDP52-200 merge.png]

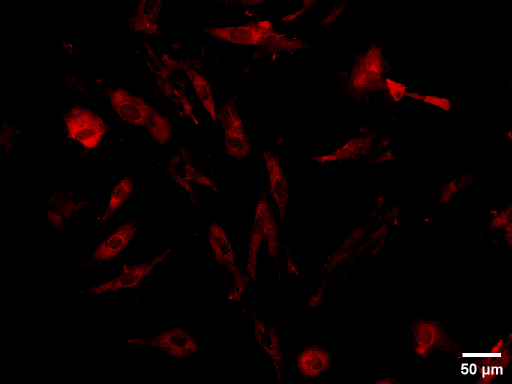

Supplement: Supplementary file 1 [file DataSheet1.zip › Data/Figure.5/Figure/IF/ULK1+NDP52/ULK1+NDP52/2GA-NDP52-200 NDP52.png]

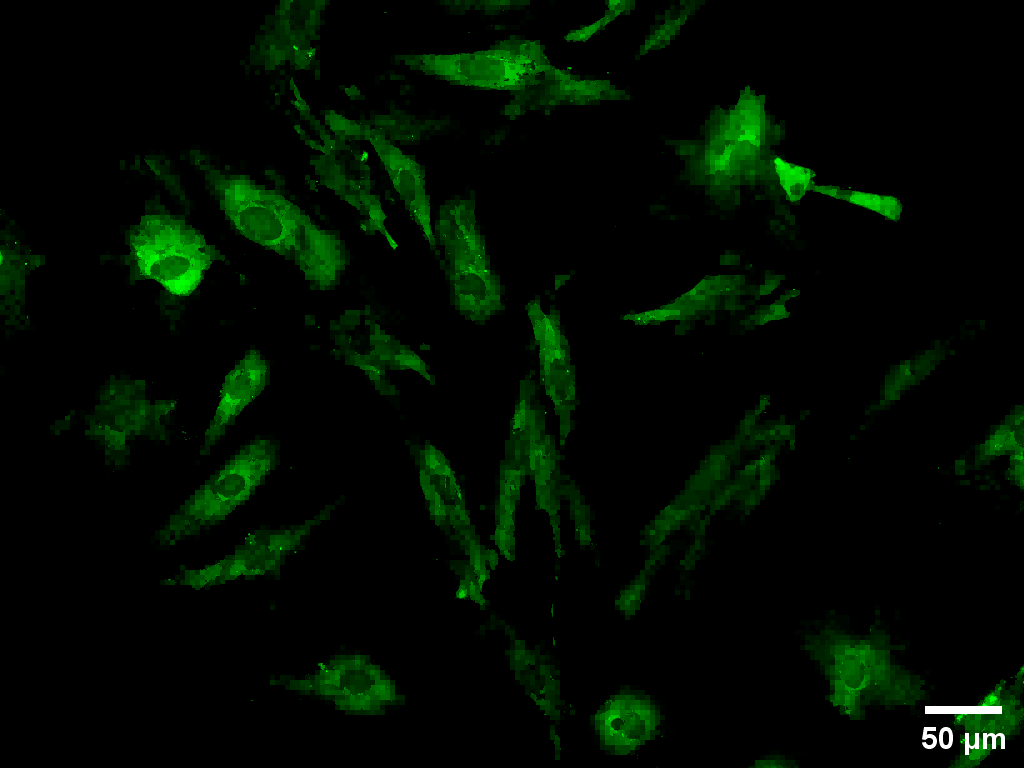

Supplement: Supplementary file 1 [file DataSheet1.zip › Data/Figure.5/Figure/IF/ULK1+NDP52/ULK1+NDP52/2GA-NDP52-200 p-ULK1.png]

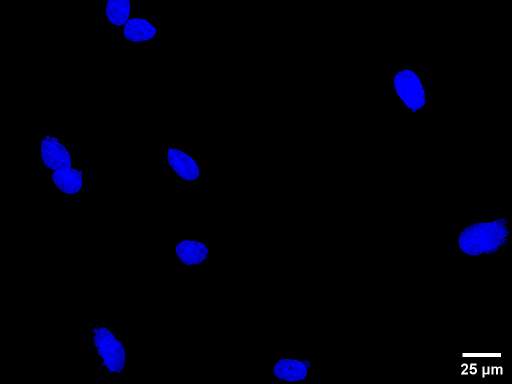

Supplement: Supplementary file 1 [file DataSheet1.zip › Data/Figure.5/Figure/IF/ULK1+NDP52/ULK1+NDP52/3GA+Naringin 1-400 DAPI.png]

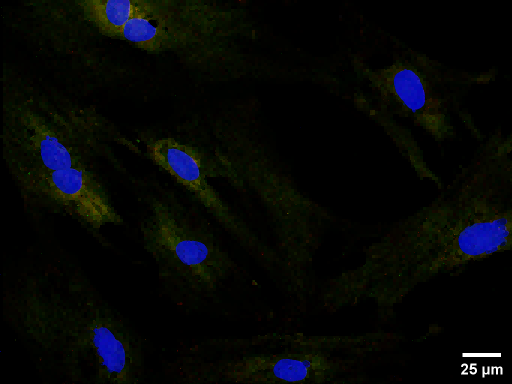

Supplement: Supplementary file 1 [file DataSheet1.zip › Data/Figure.5/Figure/IF/ULK1+NDP52/ULK1+NDP52/3GA+Naringin 1-400 merge.png]

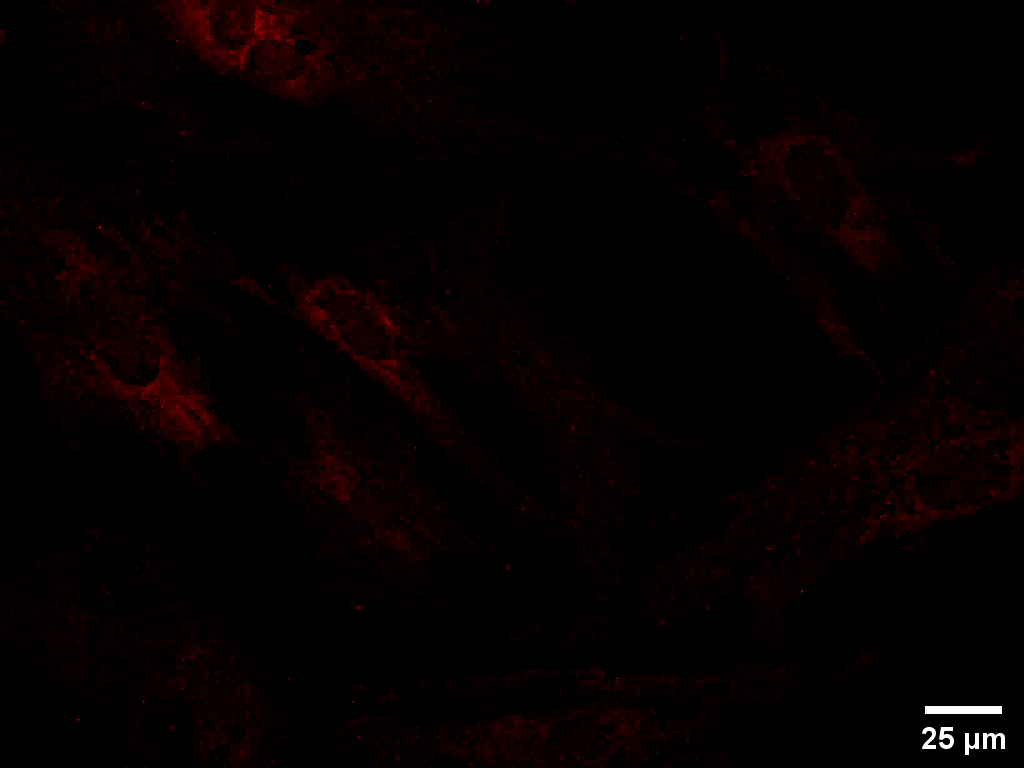

Supplement: Supplementary file 1 [file DataSheet1.zip › Data/Figure.5/Figure/IF/ULK1+NDP52/ULK1+NDP52/3GA+Naringin 1-400 NDP52.png]

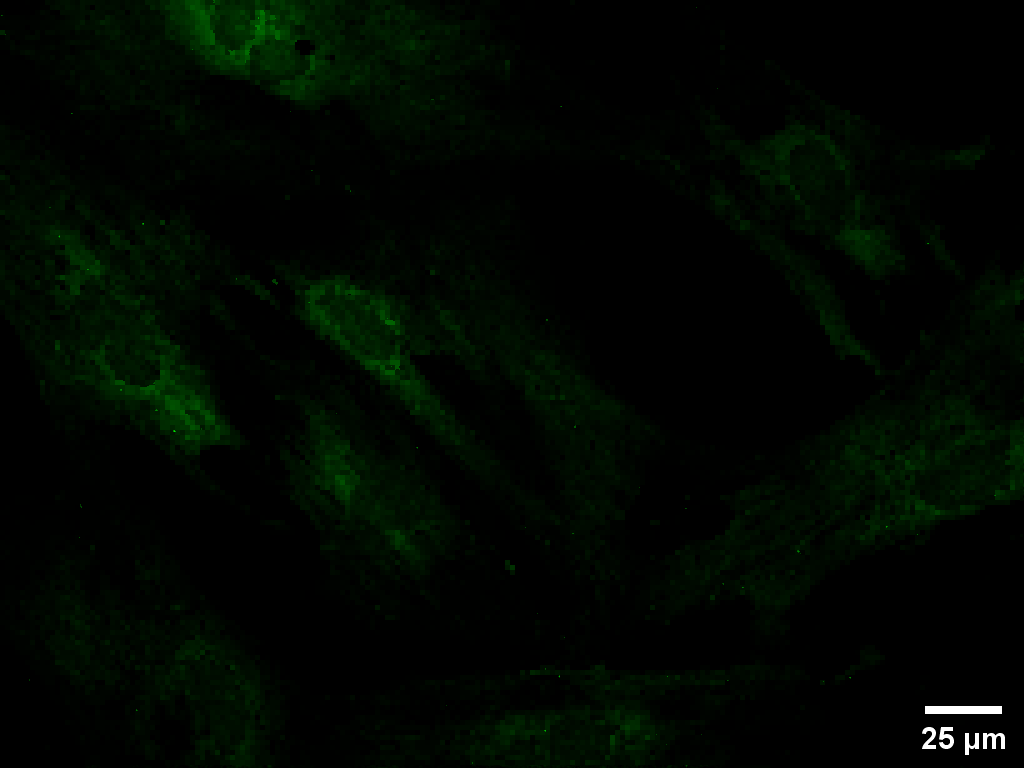

Supplement: Supplementary file 1 [file DataSheet1.zip › Data/Figure.5/Figure/IF/ULK1+NDP52/ULK1+NDP52/3GA+Naringin 1-400 p-ULK1.png]

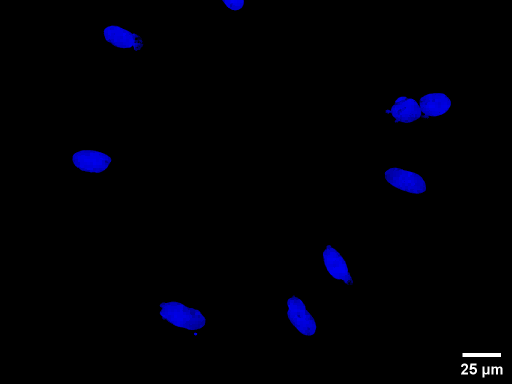

Supplement: Supplementary file 1 [file DataSheet1.zip › Data/Figure.5/Figure/IF/ULK1+NDP52/ULK1+NDP52/3GA+Naringin 2-400 DAPI.png]

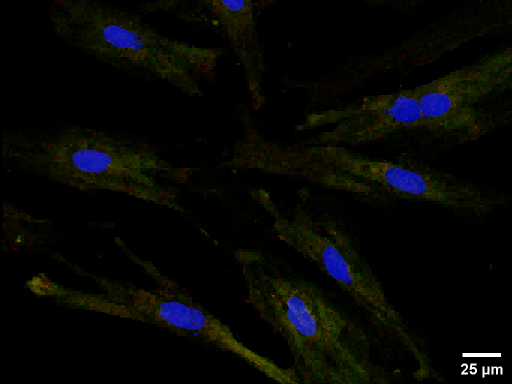

Supplement: Supplementary file 1 [file DataSheet1.zip › Data/Figure.5/Figure/IF/ULK1+NDP52/ULK1+NDP52/3GA+Naringin 2-400 merge.png]

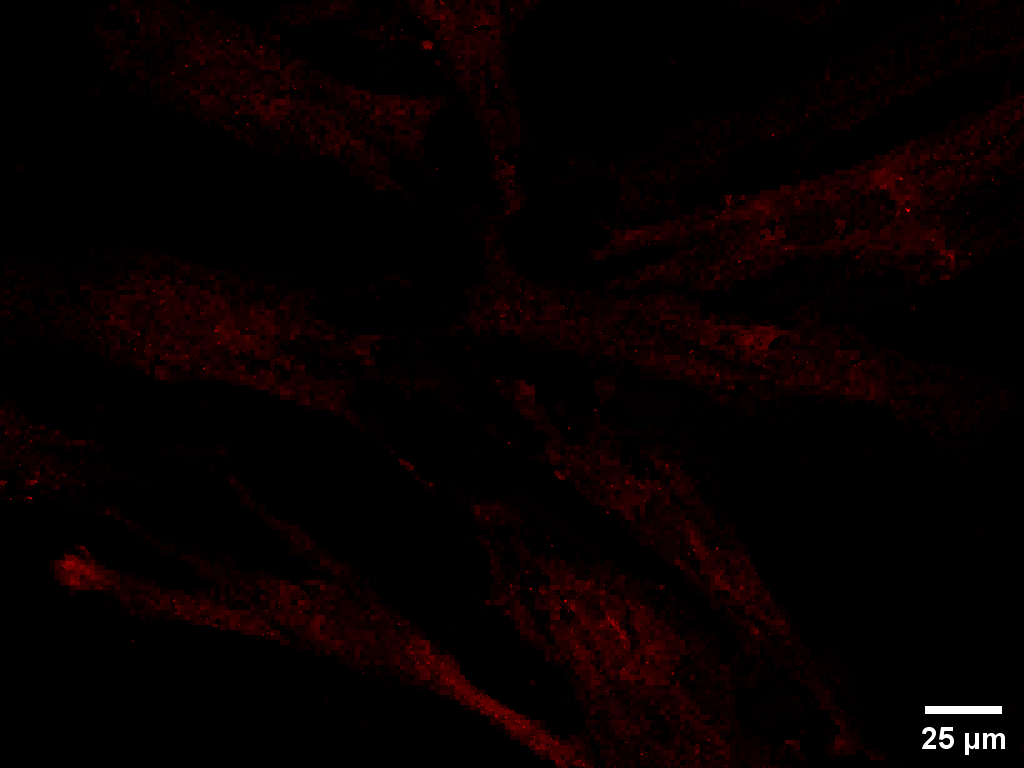

Supplement: Supplementary file 1 [file DataSheet1.zip › Data/Figure.5/Figure/IF/ULK1+NDP52/ULK1+NDP52/3GA+Naringin 2-400 NDP52.png]

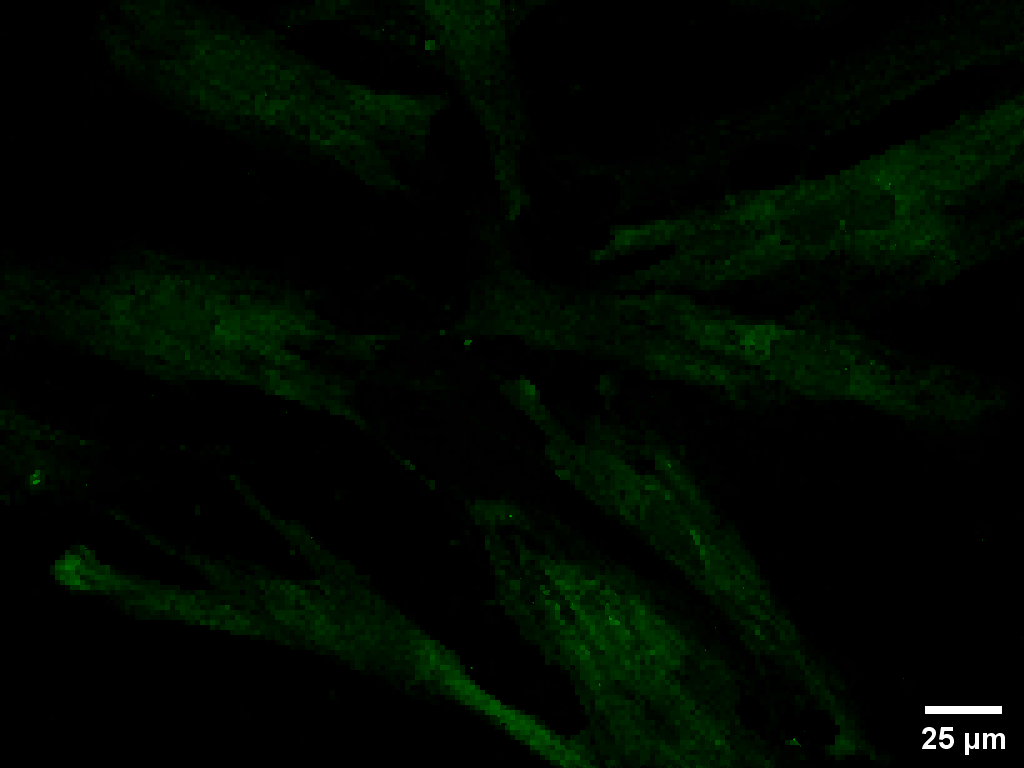

Supplement: Supplementary file 1 [file DataSheet1.zip › Data/Figure.5/Figure/IF/ULK1+NDP52/ULK1+NDP52/3GA+Naringin 2-400 p-ULK1.png]

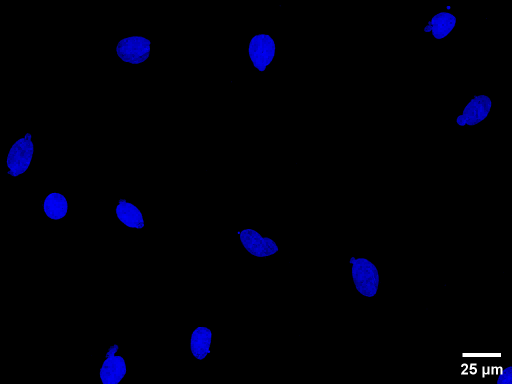

Supplement: Supplementary file 1 [file DataSheet1.zip › Data/Figure.5/Figure/IF/ULK1+NDP52/ULK1+NDP52/3GA+Naringin 3-400 DAPI.png]

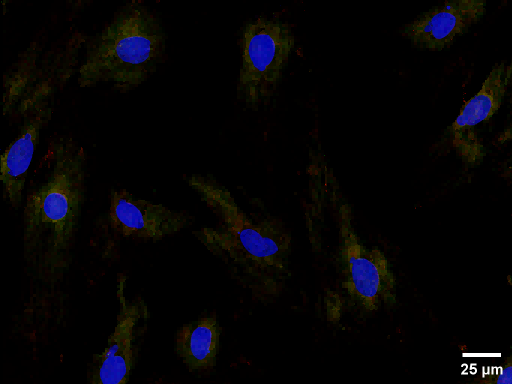

Supplement: Supplementary file 1 [file DataSheet1.zip › Data/Figure.5/Figure/IF/ULK1+NDP52/ULK1+NDP52/3GA+Naringin 3-400 merge.png]

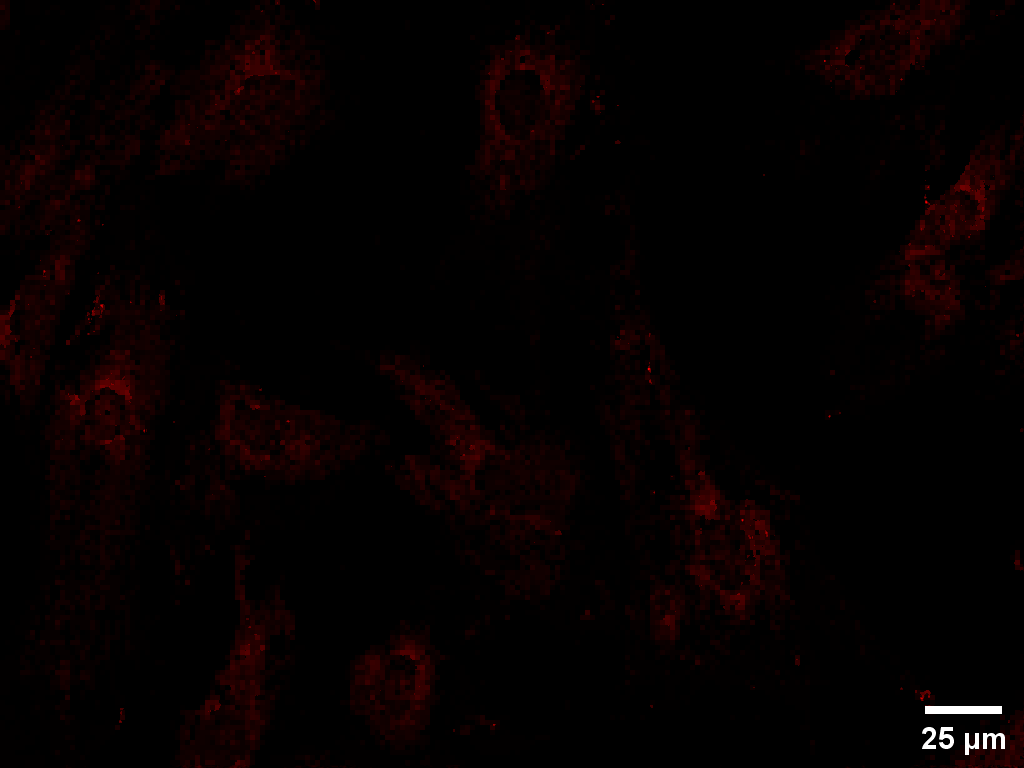

Supplement: Supplementary file 1 [file DataSheet1.zip › Data/Figure.5/Figure/IF/ULK1+NDP52/ULK1+NDP52/3GA+Naringin 3-400 NDP52.png]

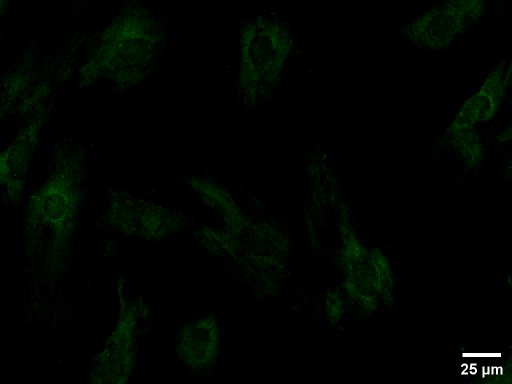

Supplement: Supplementary file 1 [file DataSheet1.zip › Data/Figure.5/Figure/IF/ULK1+NDP52/ULK1+NDP52/3GA+Naringin 3-400 p-ULK1.png]

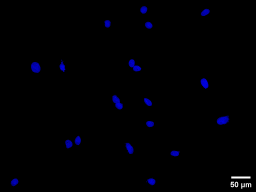

Supplement: Supplementary file 1 [file DataSheet1.zip › Data/Figure.5/Figure/IF/ULK1+NDP52/ULK1+NDP52/3GA+Naringin-200 DAPI.png]

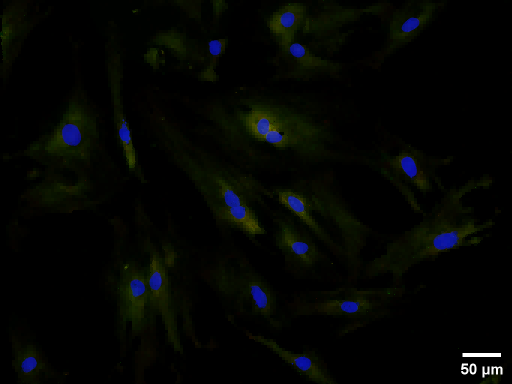

Supplement: Supplementary file 1 [file DataSheet1.zip › Data/Figure.5/Figure/IF/ULK1+NDP52/ULK1+NDP52/3GA+Naringin-200 merge.png]

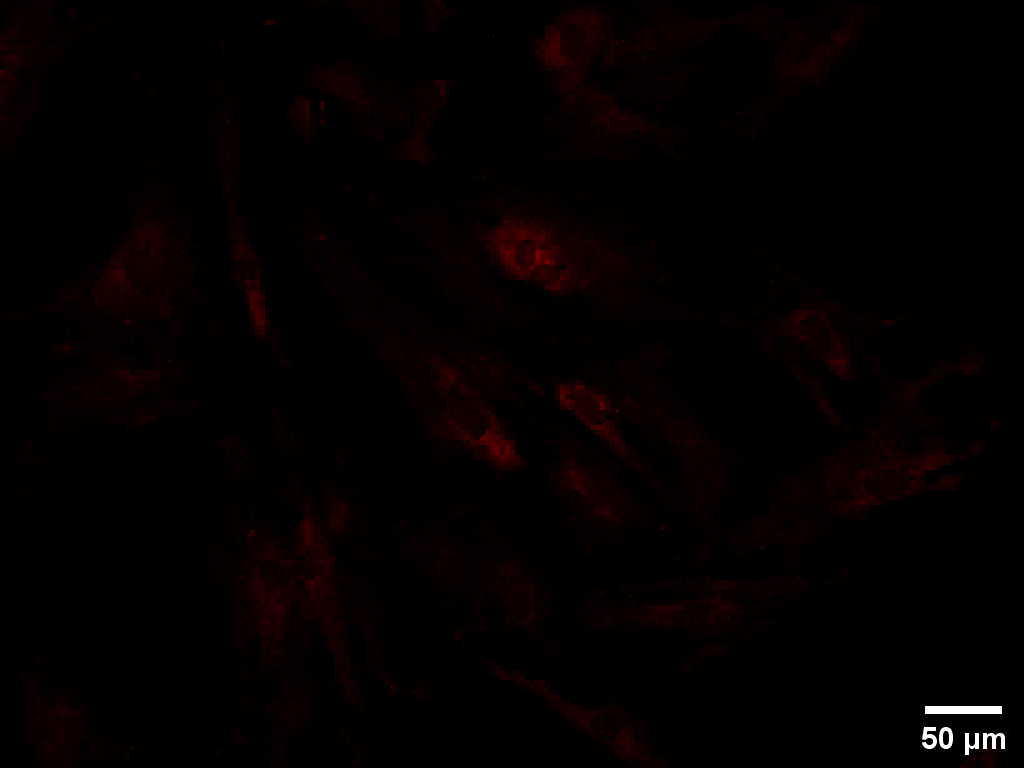

Supplement: Supplementary file 1 [file DataSheet1.zip › Data/Figure.5/Figure/IF/ULK1+NDP52/ULK1+NDP52/3GA+Naringin-200 NDP52.png]

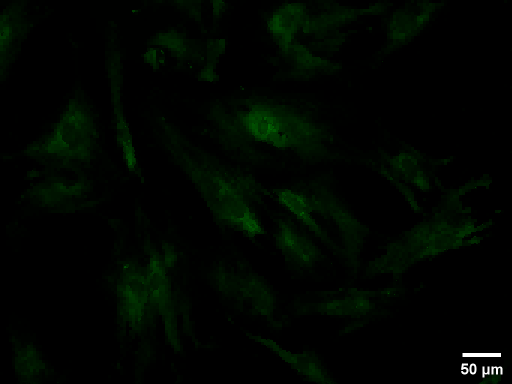

Supplement: Supplementary file 1 [file DataSheet1.zip › Data/Figure.5/Figure/IF/ULK1+NDP52/ULK1+NDP52/3GA+Naringin-200 p-ULK1.png]

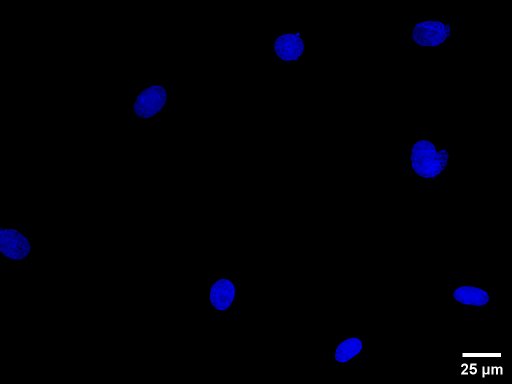

Supplement: Supplementary file 1 [file DataSheet1.zip › Data/Figure.5/Figure/IF/ULK1+NDP52/ULK1+NDP52/4GA+Naringin-NDP52 1-400 DAPI.png]

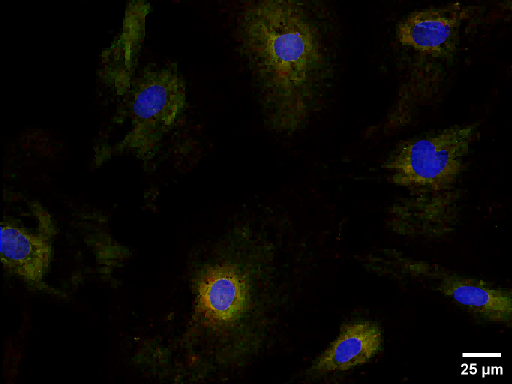

Supplement: Supplementary file 1 [file DataSheet1.zip › Data/Figure.5/Figure/IF/ULK1+NDP52/ULK1+NDP52/4GA+Naringin-NDP52 1-400 merge.png]

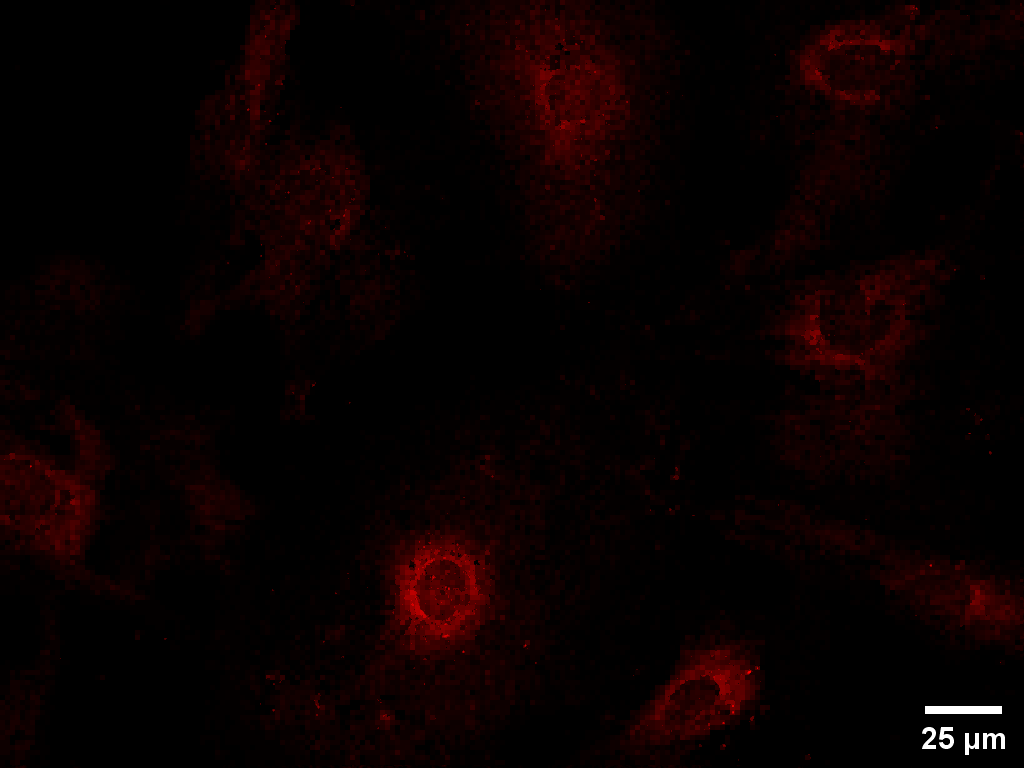

Supplement: Supplementary file 1 [file DataSheet1.zip › Data/Figure.5/Figure/IF/ULK1+NDP52/ULK1+NDP52/4GA+Naringin-NDP52 1-400 NDP52.png]

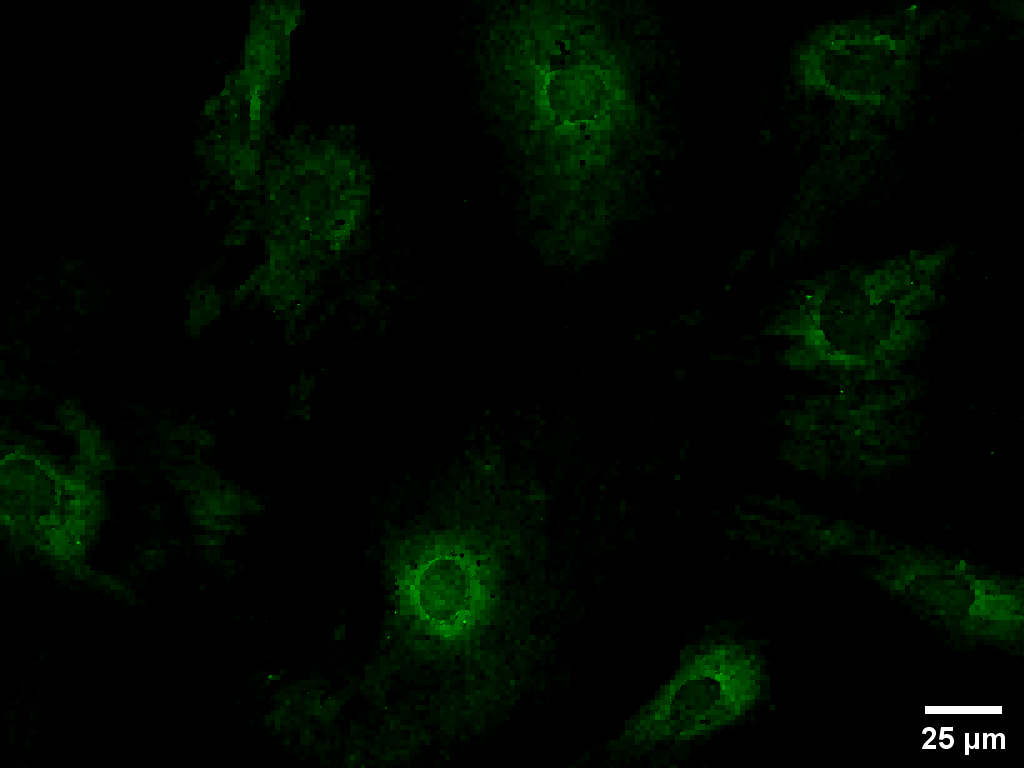

Supplement: Supplementary file 1 [file DataSheet1.zip › Data/Figure.5/Figure/IF/ULK1+NDP52/ULK1+NDP52/4GA+Naringin-NDP52 1-400 p-ULK1.png]

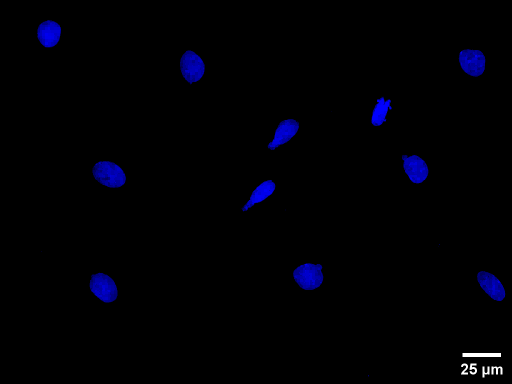

Supplement: Supplementary file 1 [file DataSheet1.zip › Data/Figure.5/Figure/IF/ULK1+NDP52/ULK1+NDP52/4GA+Naringin-NDP52 2-400 DAPI.png]

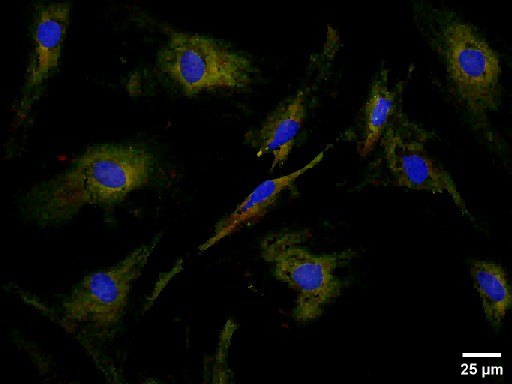

Supplement: Supplementary file 1 [file DataSheet1.zip › Data/Figure.5/Figure/IF/ULK1+NDP52/ULK1+NDP52/4GA+Naringin-NDP52 2-400 merge.png]

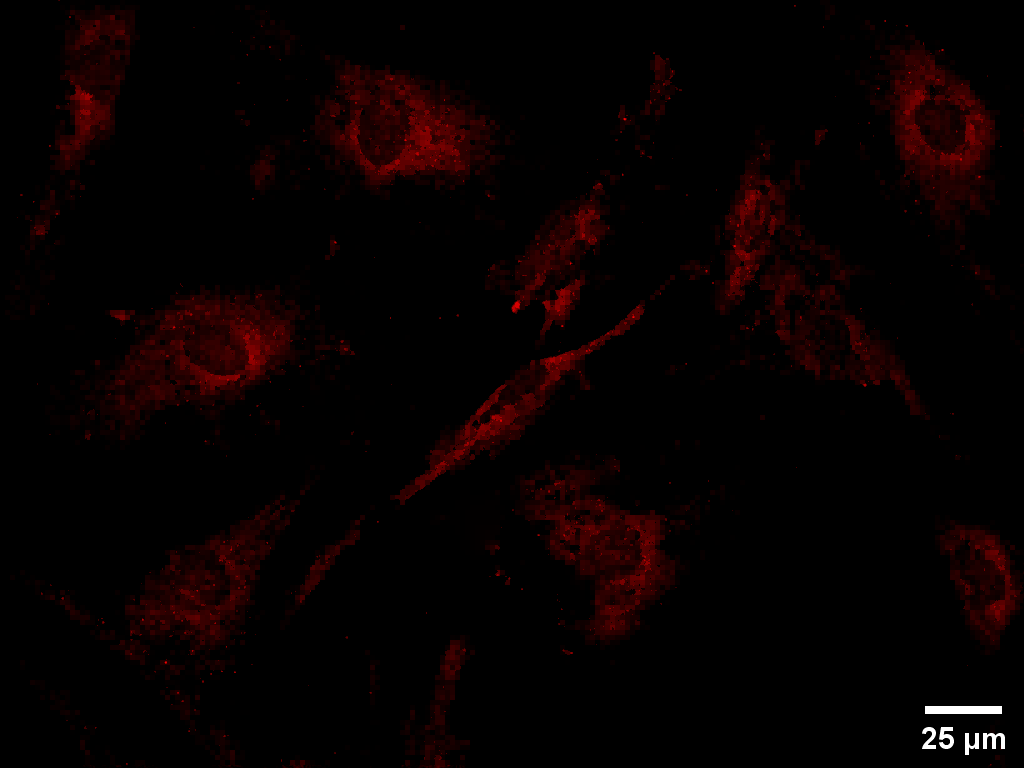

Supplement: Supplementary file 1 [file DataSheet1.zip › Data/Figure.5/Figure/IF/ULK1+NDP52/ULK1+NDP52/4GA+Naringin-NDP52 2-400 NDP52.png]

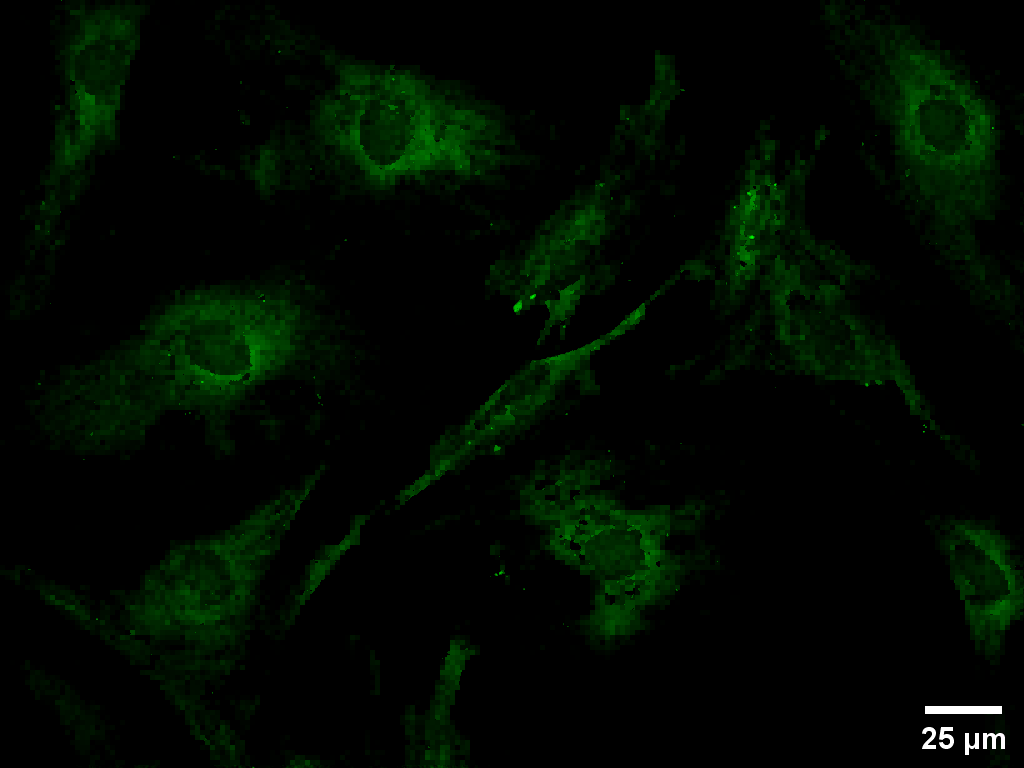

Supplement: Supplementary file 1 [file DataSheet1.zip › Data/Figure.5/Figure/IF/ULK1+NDP52/ULK1+NDP52/4GA+Naringin-NDP52 2-400 p-ULK1.png]

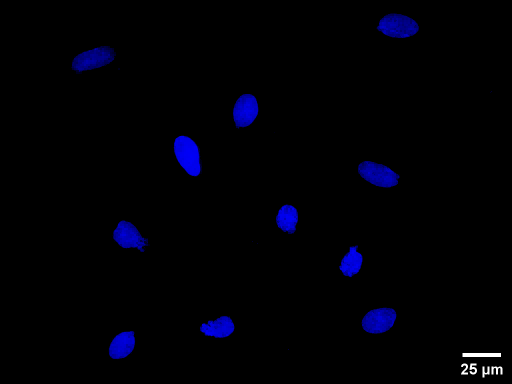

Supplement: Supplementary file 1 [file DataSheet1.zip › Data/Figure.5/Figure/IF/ULK1+NDP52/ULK1+NDP52/4GA+Naringin-NDP52 3-400 DAPI.png]

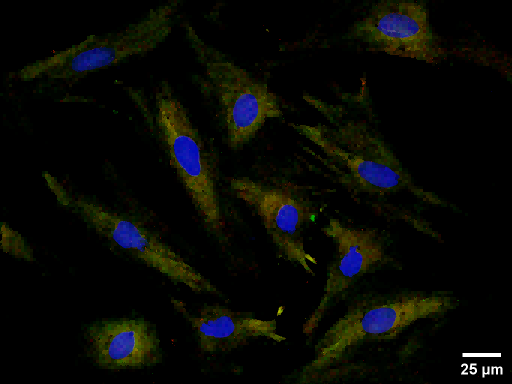

Supplement: Supplementary file 1 [file DataSheet1.zip › Data/Figure.5/Figure/IF/ULK1+NDP52/ULK1+NDP52/4GA+Naringin-NDP52 3-400 merge.png]

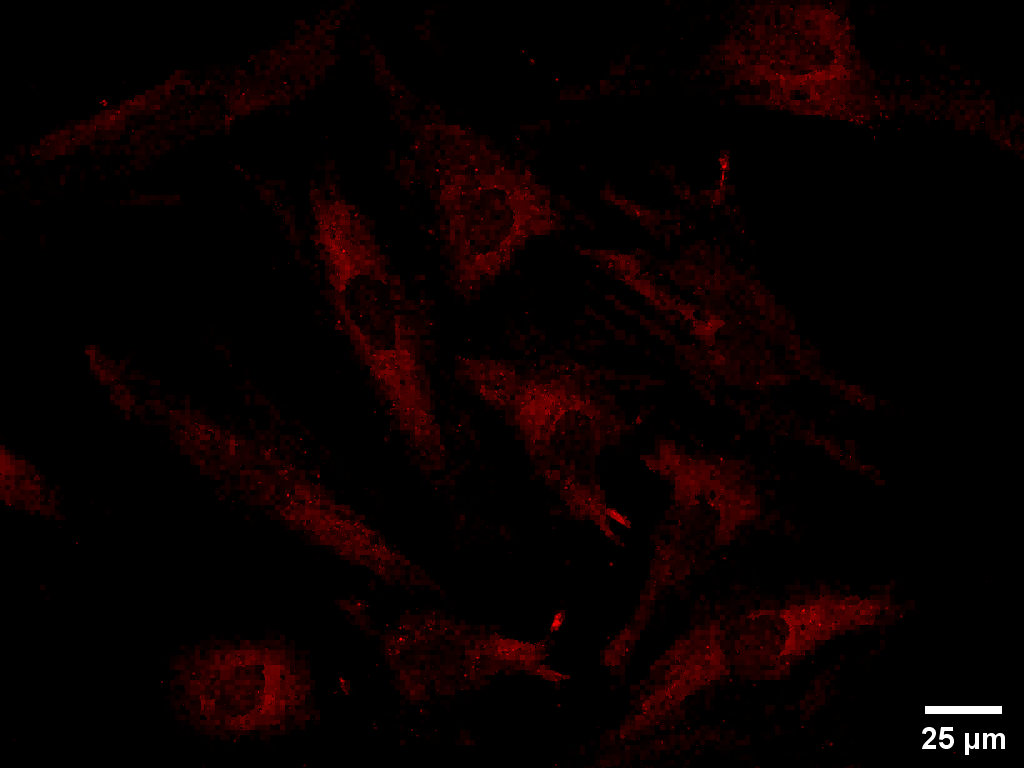

Supplement: Supplementary file 1 [file DataSheet1.zip › Data/Figure.5/Figure/IF/ULK1+NDP52/ULK1+NDP52/4GA+Naringin-NDP52 3-400 NDP52.png]

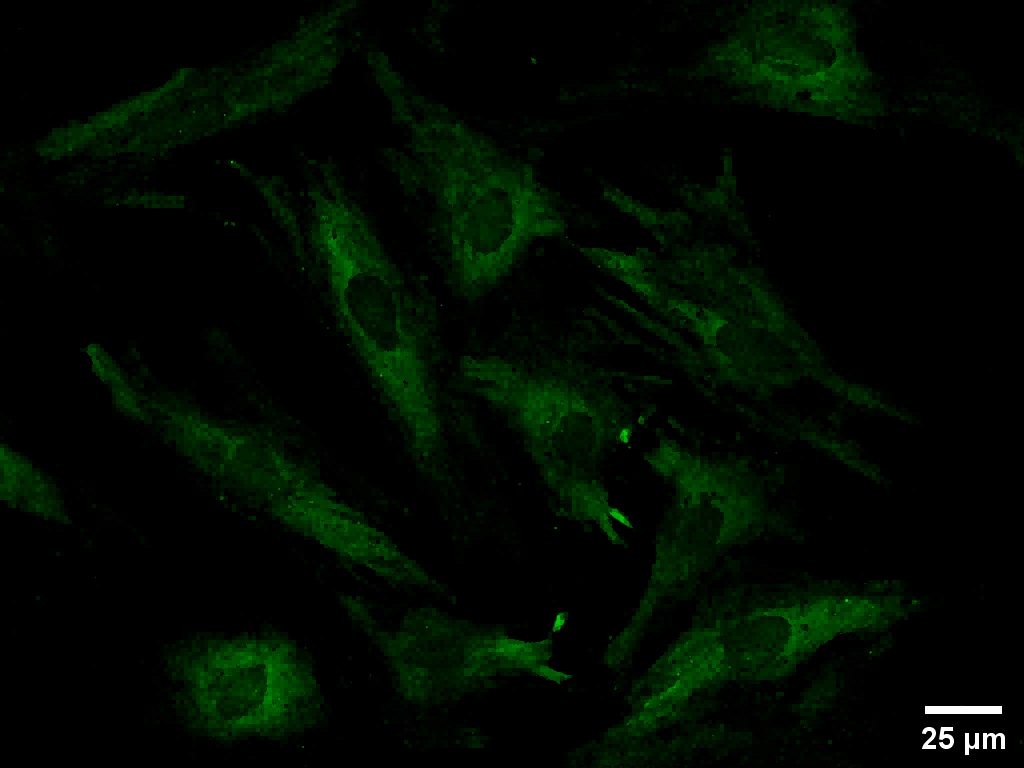

Supplement: Supplementary file 1 [file DataSheet1.zip › Data/Figure.5/Figure/IF/ULK1+NDP52/ULK1+NDP52/4GA+Naringin-NDP52 3-400 p-ULK1.png]
