# Supplementary figures and images for: Naringenin attenuates slow-transit constipation by regulating the AMPK/mTOR/ULK1 signalling pathway: in vivo and in vitro studies (part 1 of 2)
Source: Front Pharmacol. 2025 Jun 17;16:1550458. doi: 10.3389/fphar.2025.1550458 (PMC12209368; doi:10.3389/fphar.2025.1550458)

Fig.1C

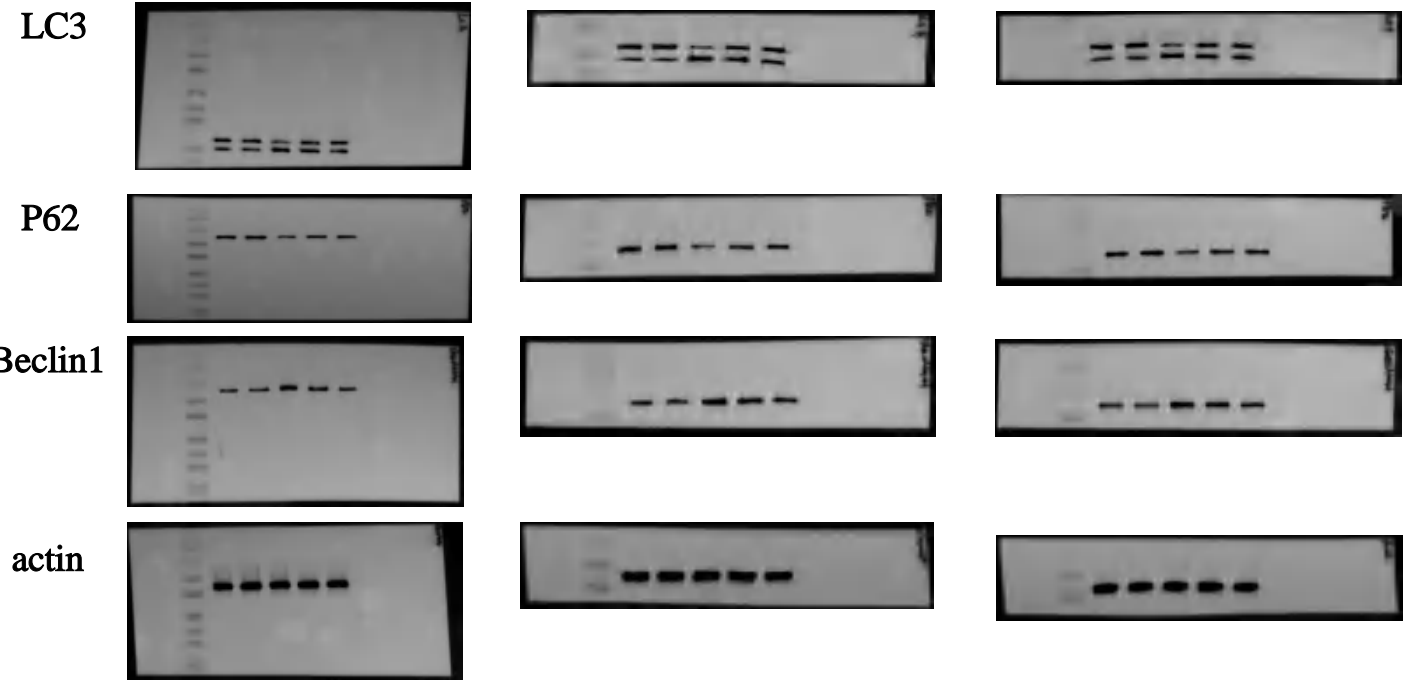

Fig.1E

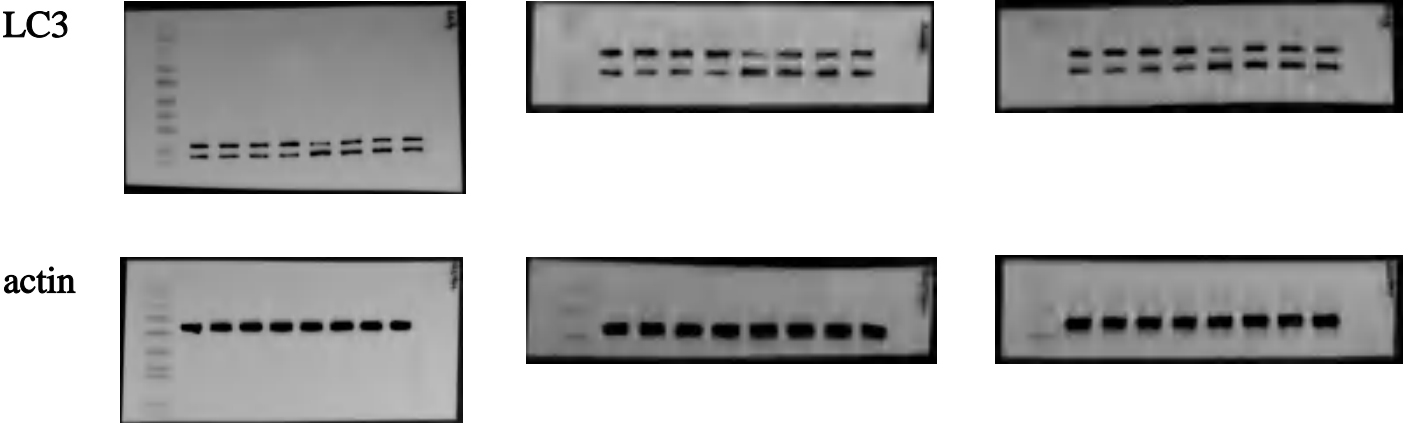

Supplement: Supplementary file 1 [file DataSheet1.zip › Data/Figure.1/Figure/Western Blot.pdf]

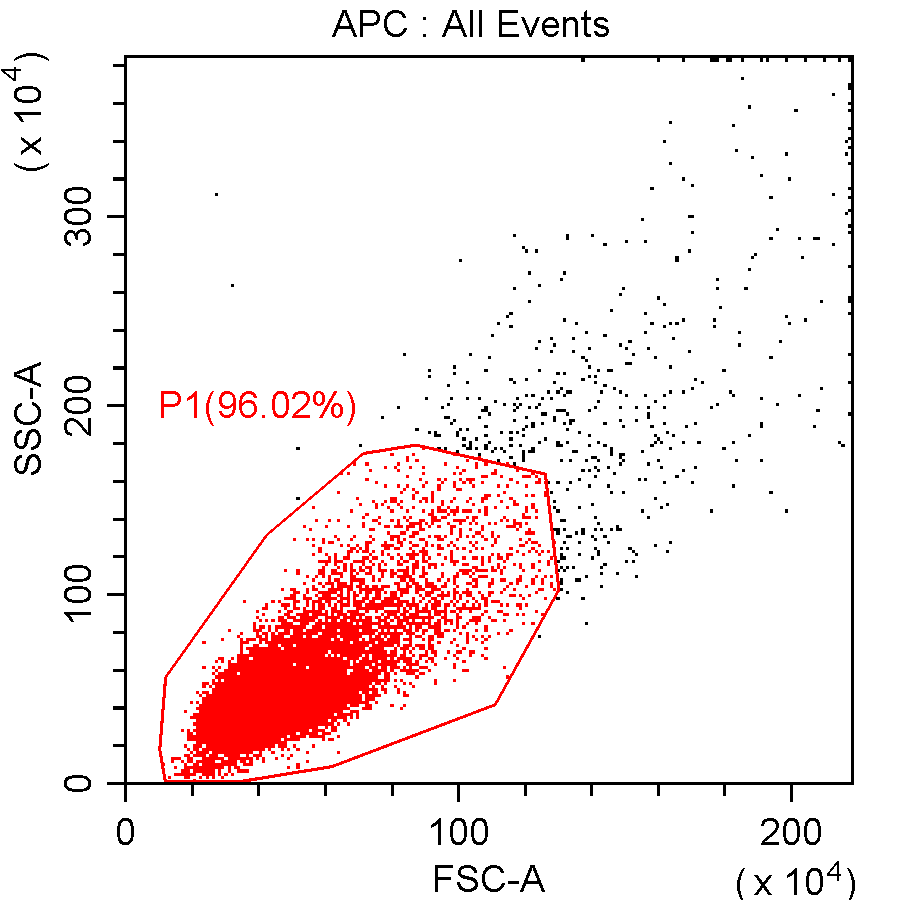

Supplement: Supplementary file 1 [file DataSheet1.zip › Data/Figure.2/Figure/flow cytometry data/APC_Plot1.png]

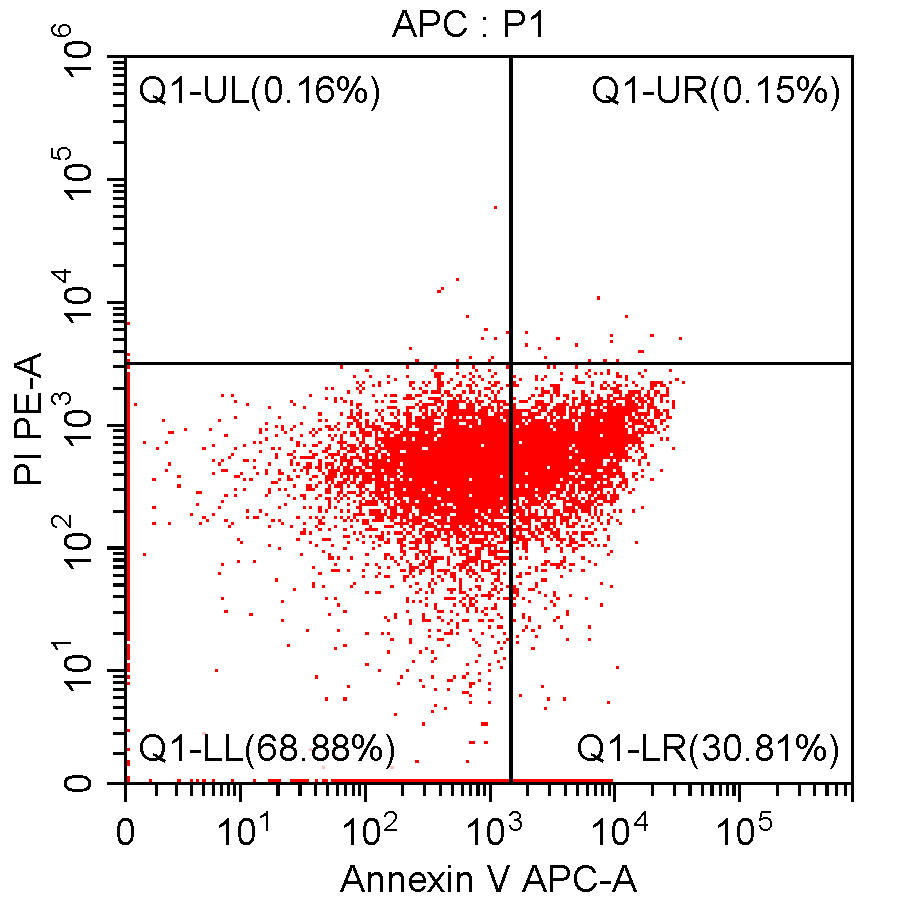

Supplement: Supplementary file 1 [file DataSheet1.zip › Data/Figure.2/Figure/flow cytometry data/APC_Plot2.png]

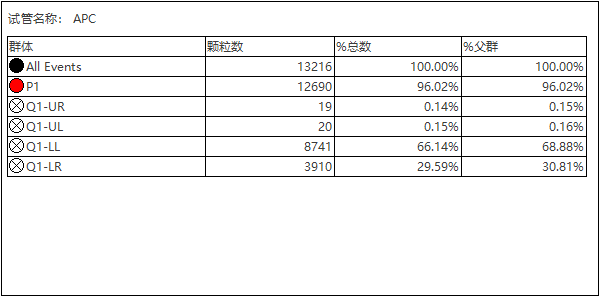

Supplement: Supplementary file 1 [file DataSheet1.zip › Data/Figure.2/Figure/flow cytometry data/APC_Statistics1.png]

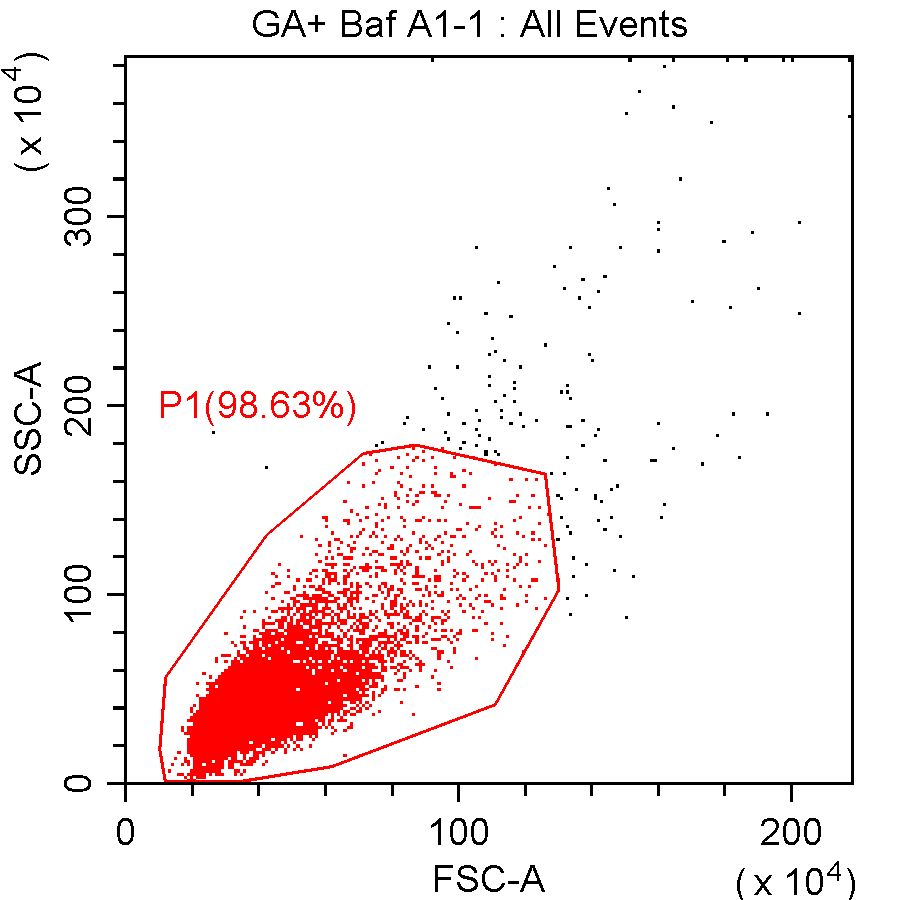

Supplement: Supplementary file 1 [file DataSheet1.zip › Data/Figure.2/Figure/flow cytometry data/GA+ Baf A1-1_Plot1.png]

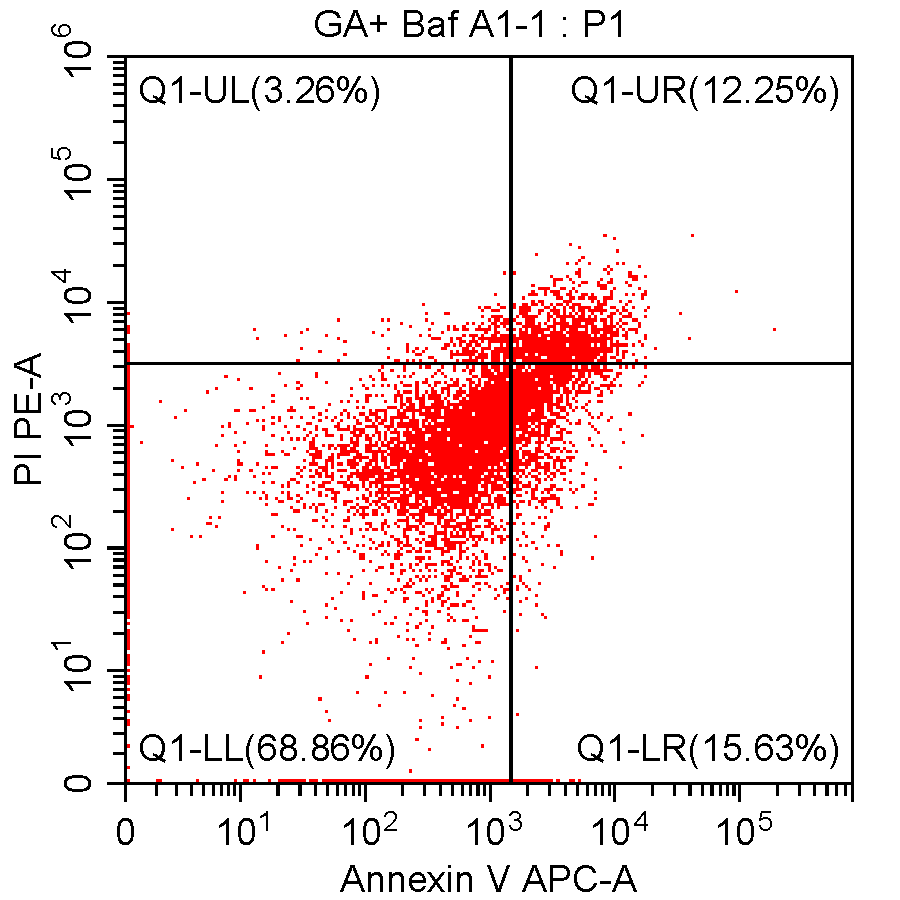

Supplement: Supplementary file 1 [file DataSheet1.zip › Data/Figure.2/Figure/flow cytometry data/GA+ Baf A1-1_Plot2.png]

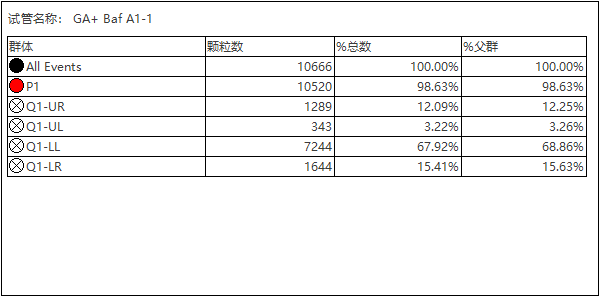

Supplement: Supplementary file 1 [file DataSheet1.zip › Data/Figure.2/Figure/flow cytometry data/GA+ Baf A1-1_Statistics1.png]

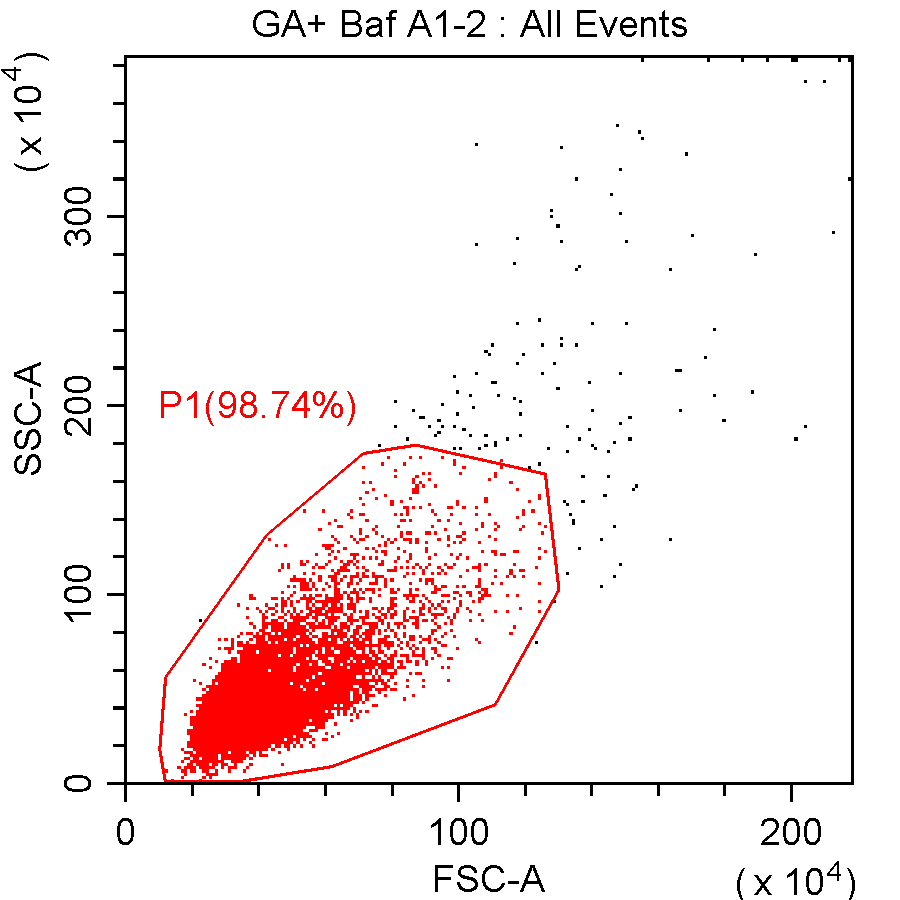

Supplement: Supplementary file 1 [file DataSheet1.zip › Data/Figure.2/Figure/flow cytometry data/GA+ Baf A1-2_Plot1.png]

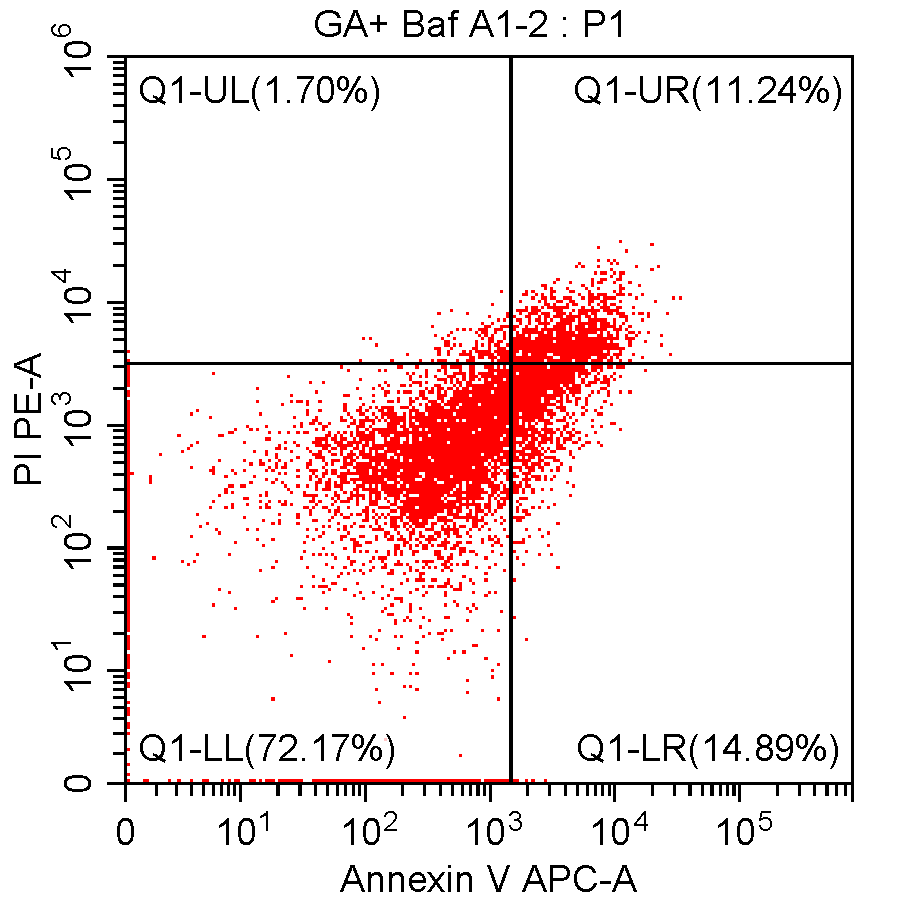

Supplement: Supplementary file 1 [file DataSheet1.zip › Data/Figure.2/Figure/flow cytometry data/GA+ Baf A1-2_Plot2.png]

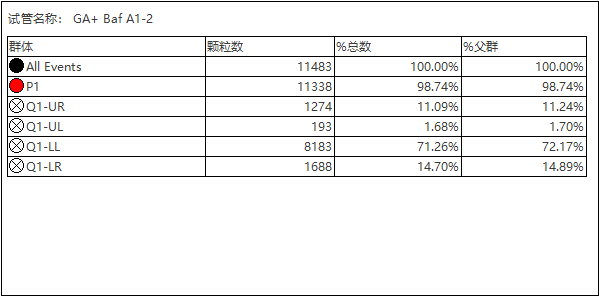

Supplement: Supplementary file 1 [file DataSheet1.zip › Data/Figure.2/Figure/flow cytometry data/GA+ Baf A1-2_Statistics1.png]

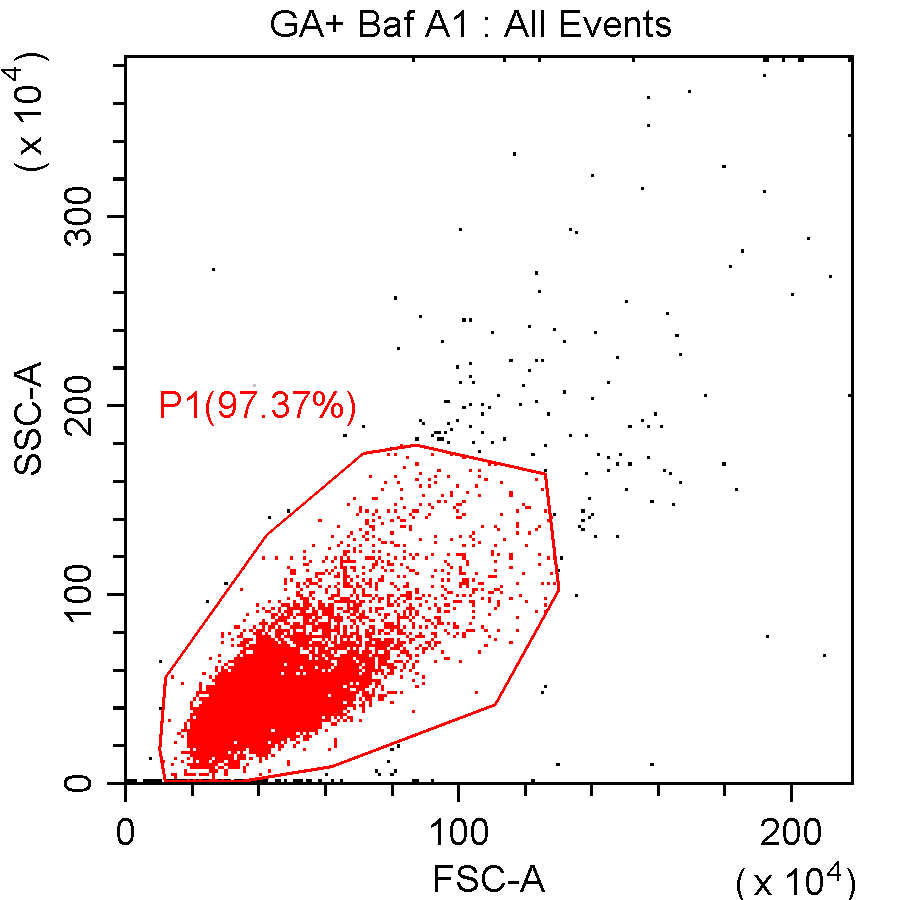

Supplement: Supplementary file 1 [file DataSheet1.zip › Data/Figure.2/Figure/flow cytometry data/GA+ Baf A1_Plot1.png]

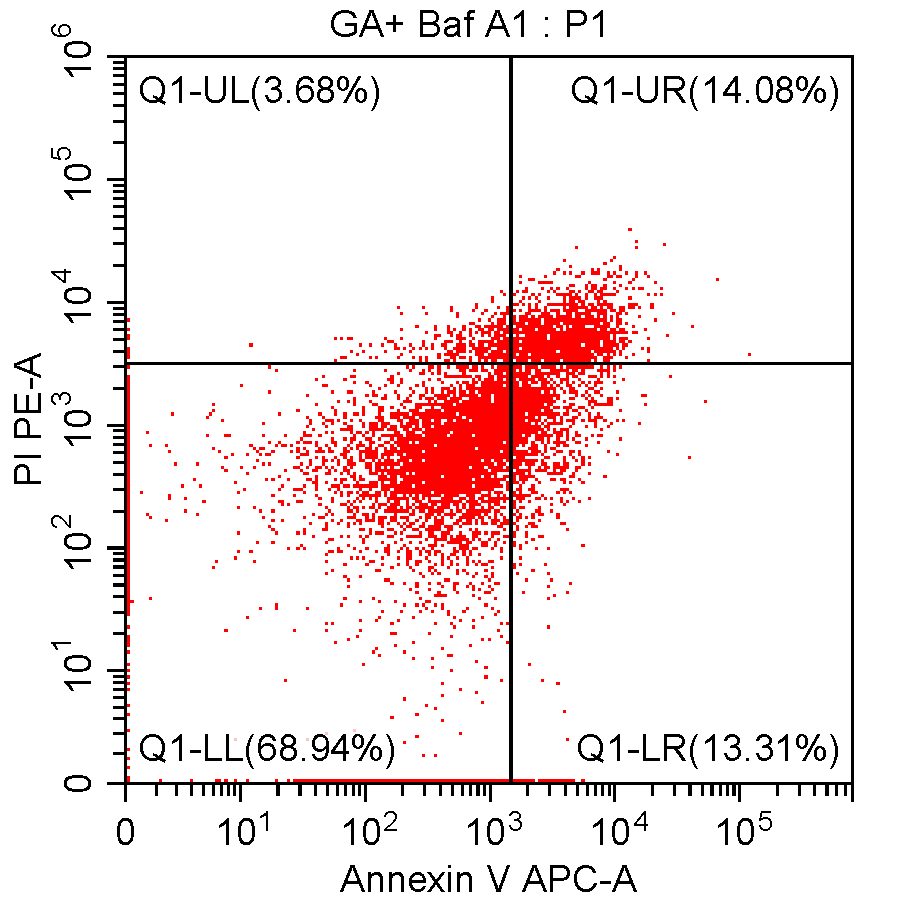

Supplement: Supplementary file 1 [file DataSheet1.zip › Data/Figure.2/Figure/flow cytometry data/GA+ Baf A1_Plot2.png]

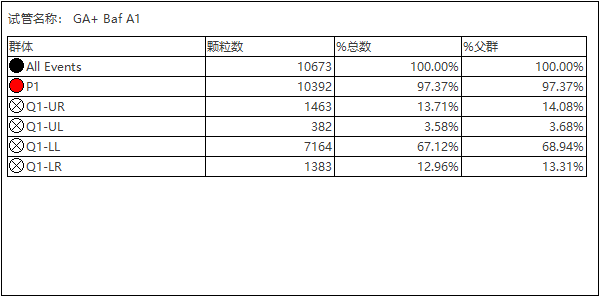

Supplement: Supplementary file 1 [file DataSheet1.zip › Data/Figure.2/Figure/flow cytometry data/GA+ Baf A1_Statistics1.png]

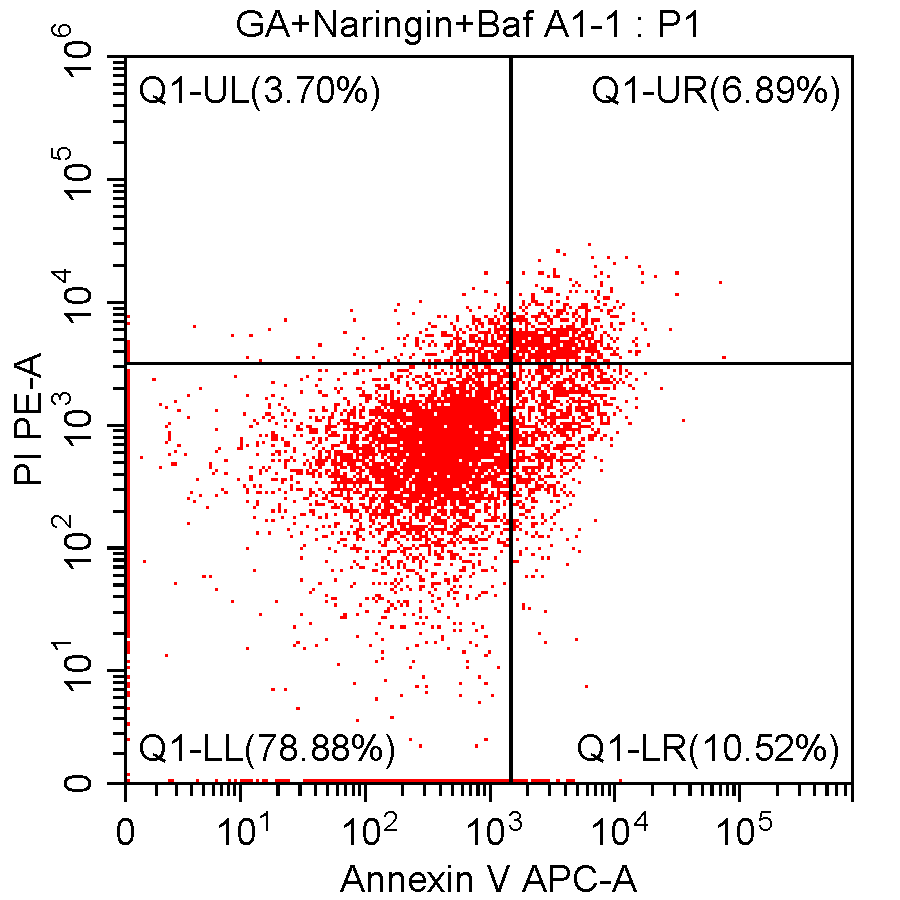

Supplement: Supplementary file 1 [file DataSheet1.zip › Data/Figure.2/Figure/flow cytometry data/GA+Naringin+Baf A1-1_Plot2.png]

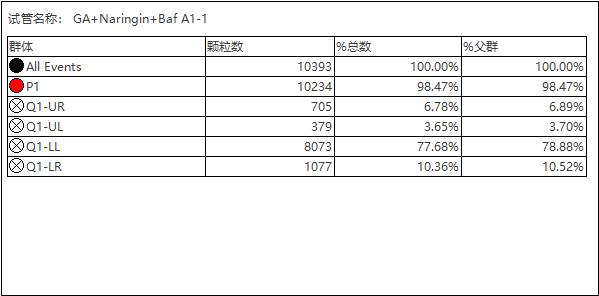

Supplement: Supplementary file 1 [file DataSheet1.zip › Data/Figure.2/Figure/flow cytometry data/GA+Naringin+Baf A1-1_Statistics1.png]

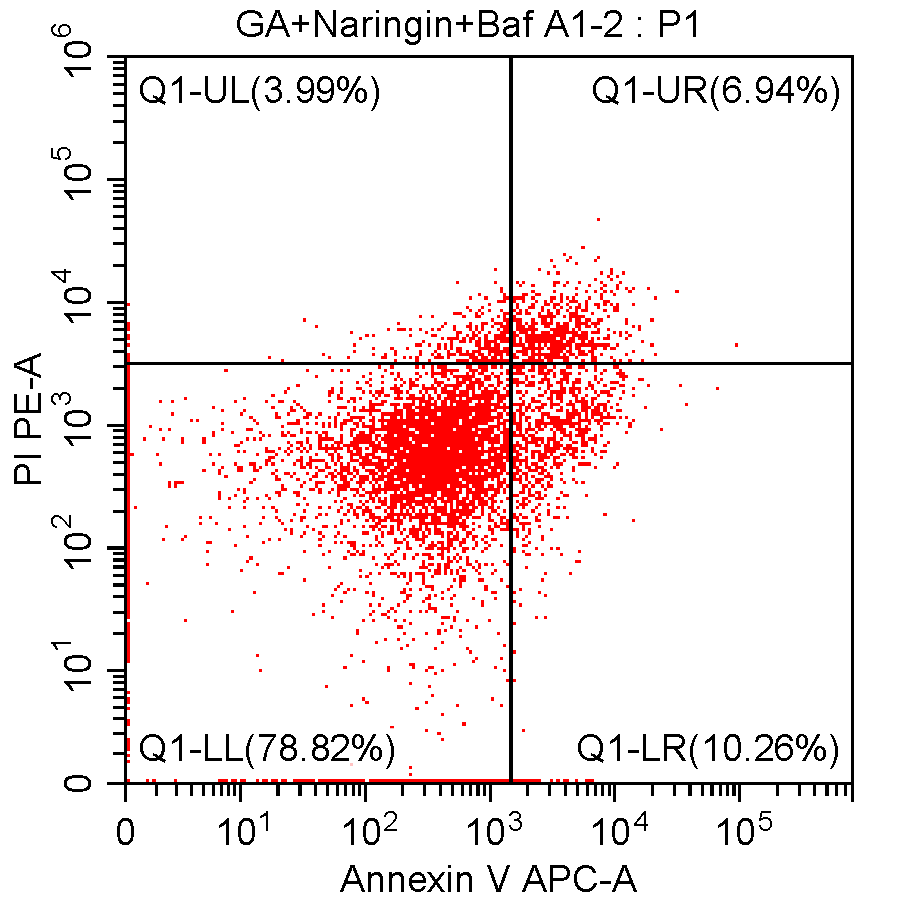

Supplement: Supplementary file 1 [file DataSheet1.zip › Data/Figure.2/Figure/flow cytometry data/GA+Naringin+Baf A1-2_Plot2.png]

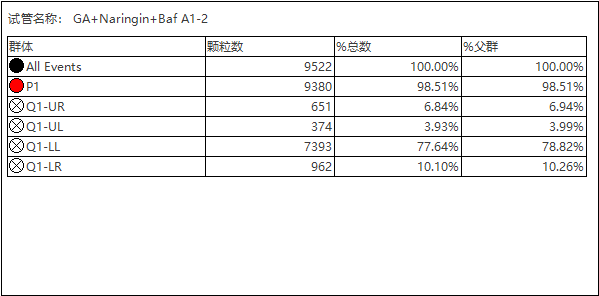

Supplement: Supplementary file 1 [file DataSheet1.zip › Data/Figure.2/Figure/flow cytometry data/GA+Naringin+Baf A1-2_Statistics1.png]

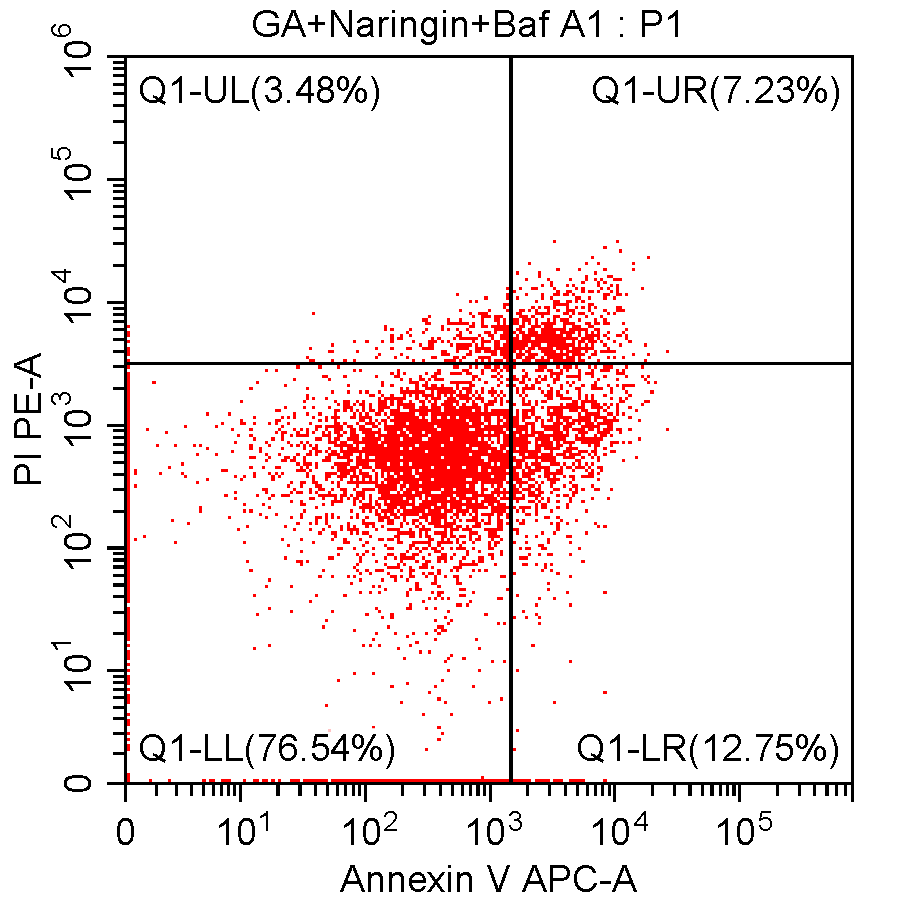

Supplement: Supplementary file 1 [file DataSheet1.zip › Data/Figure.2/Figure/flow cytometry data/GA+Naringin+Baf A1_Plot2.png]

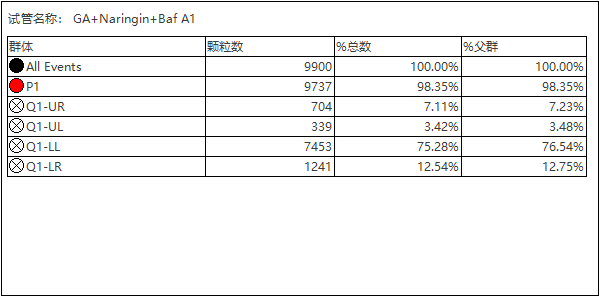

Supplement: Supplementary file 1 [file DataSheet1.zip › Data/Figure.2/Figure/flow cytometry data/GA+Naringin+Baf A1_Statistics1.png]

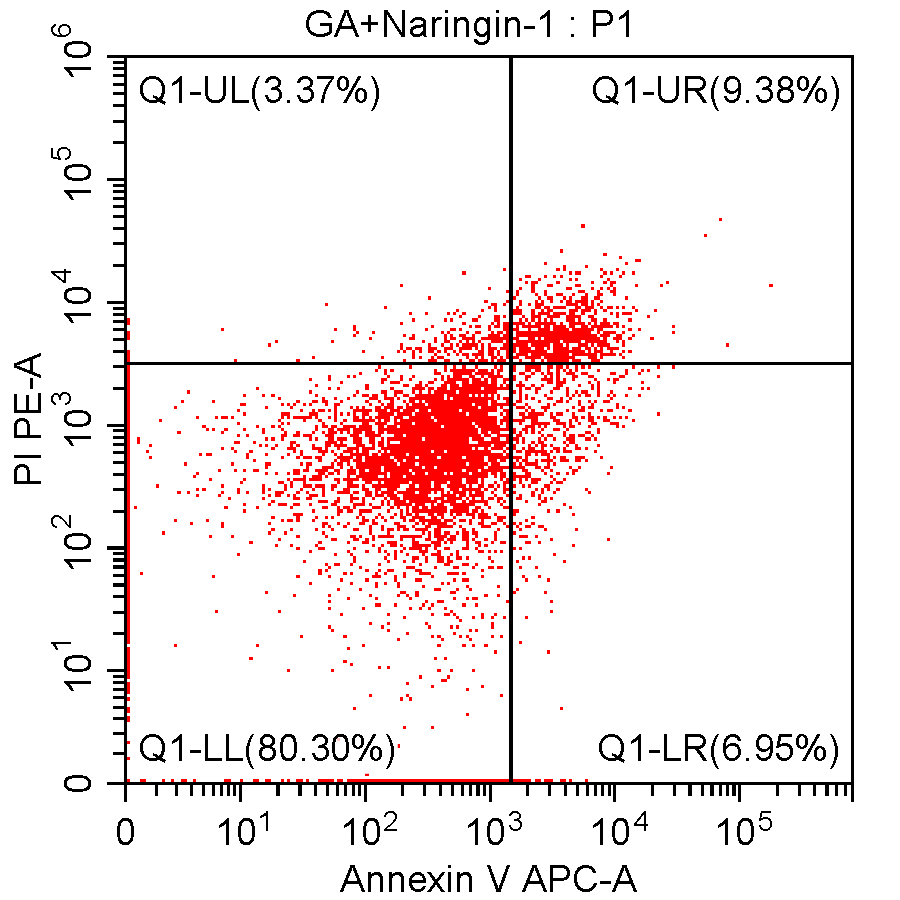

Supplement: Supplementary file 1 [file DataSheet1.zip › Data/Figure.2/Figure/flow cytometry data/GA+Naringin-1_Plot2.png]

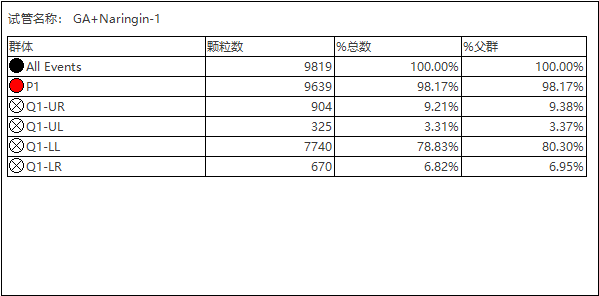

Supplement: Supplementary file 1 [file DataSheet1.zip › Data/Figure.2/Figure/flow cytometry data/GA+Naringin-1_Statistics1.png]

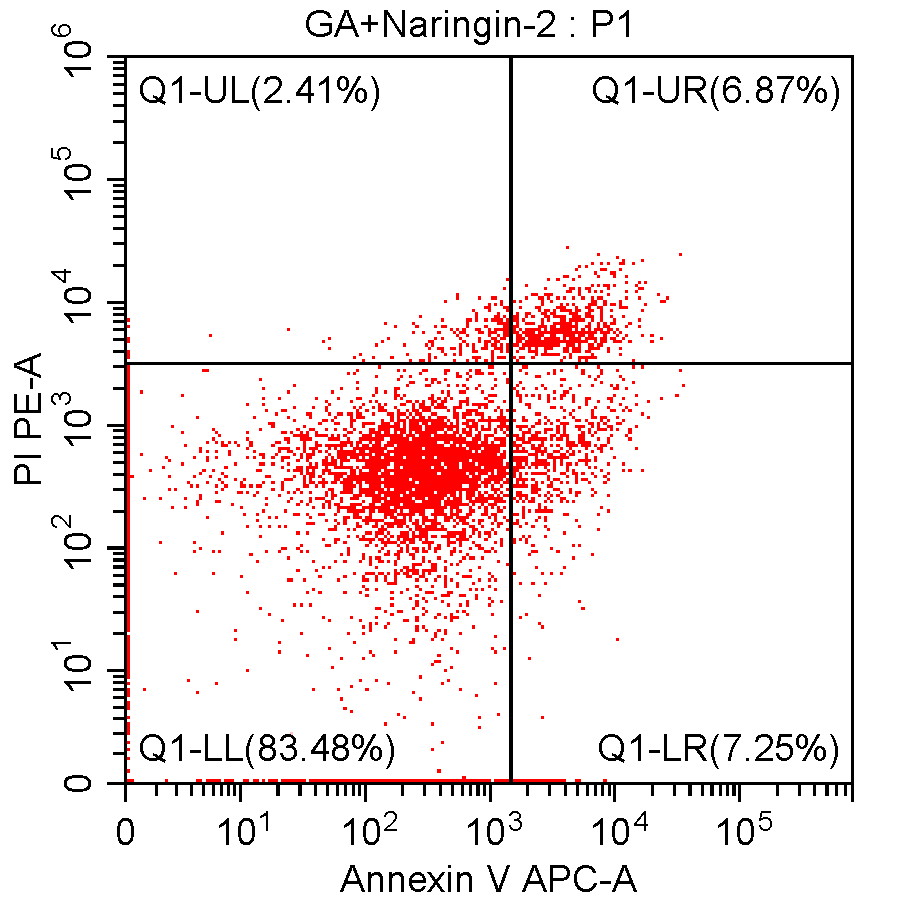

Supplement: Supplementary file 1 [file DataSheet1.zip › Data/Figure.2/Figure/flow cytometry data/GA+Naringin-2_Plot2.png]

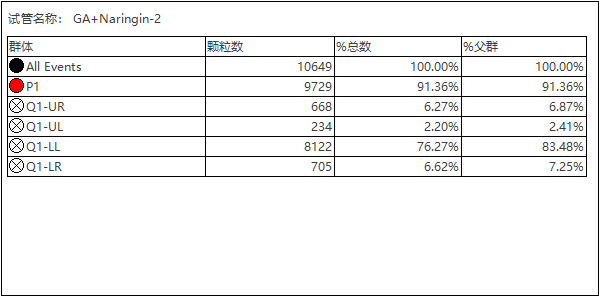

Supplement: Supplementary file 1 [file DataSheet1.zip › Data/Figure.2/Figure/flow cytometry data/GA+Naringin-2_Statistics1.png]

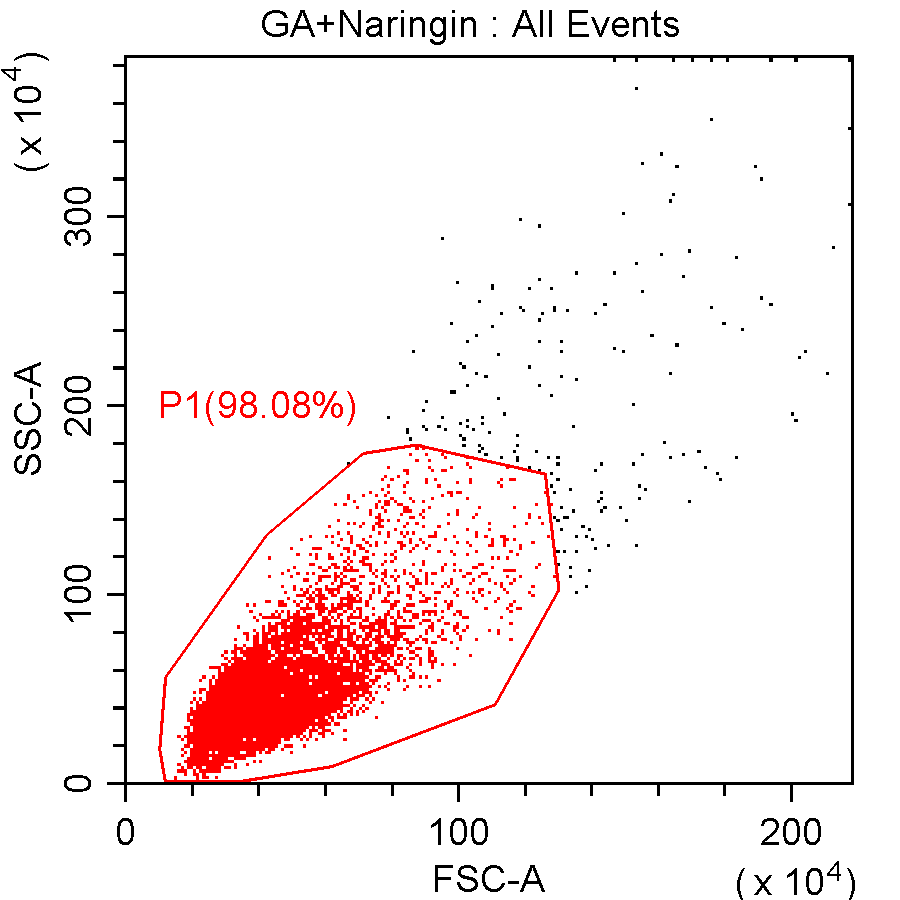

Supplement: Supplementary file 1 [file DataSheet1.zip › Data/Figure.2/Figure/flow cytometry data/GA+Naringin_Plot1.png]

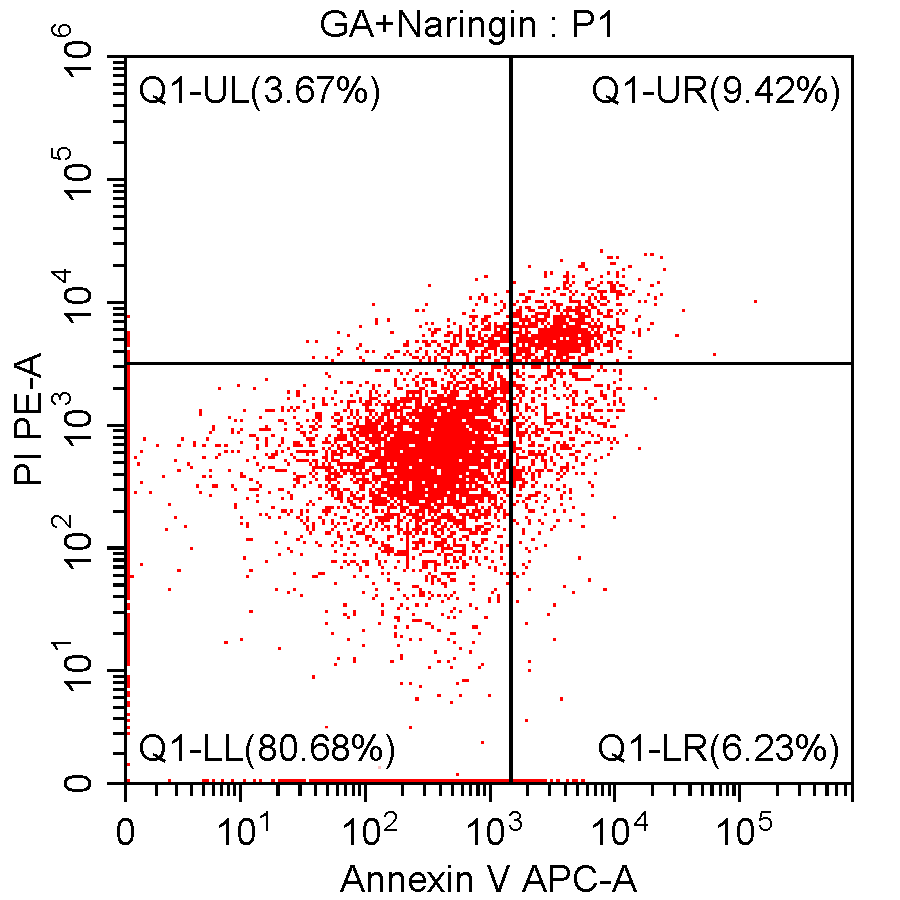

Supplement: Supplementary file 1 [file DataSheet1.zip › Data/Figure.2/Figure/flow cytometry data/GA+Naringin_Plot2.png]

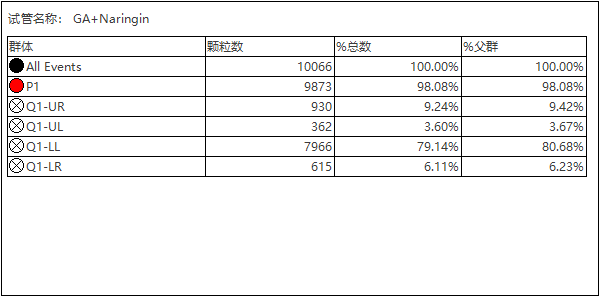

Supplement: Supplementary file 1 [file DataSheet1.zip › Data/Figure.2/Figure/flow cytometry data/GA+Naringin_Statistics1.png]

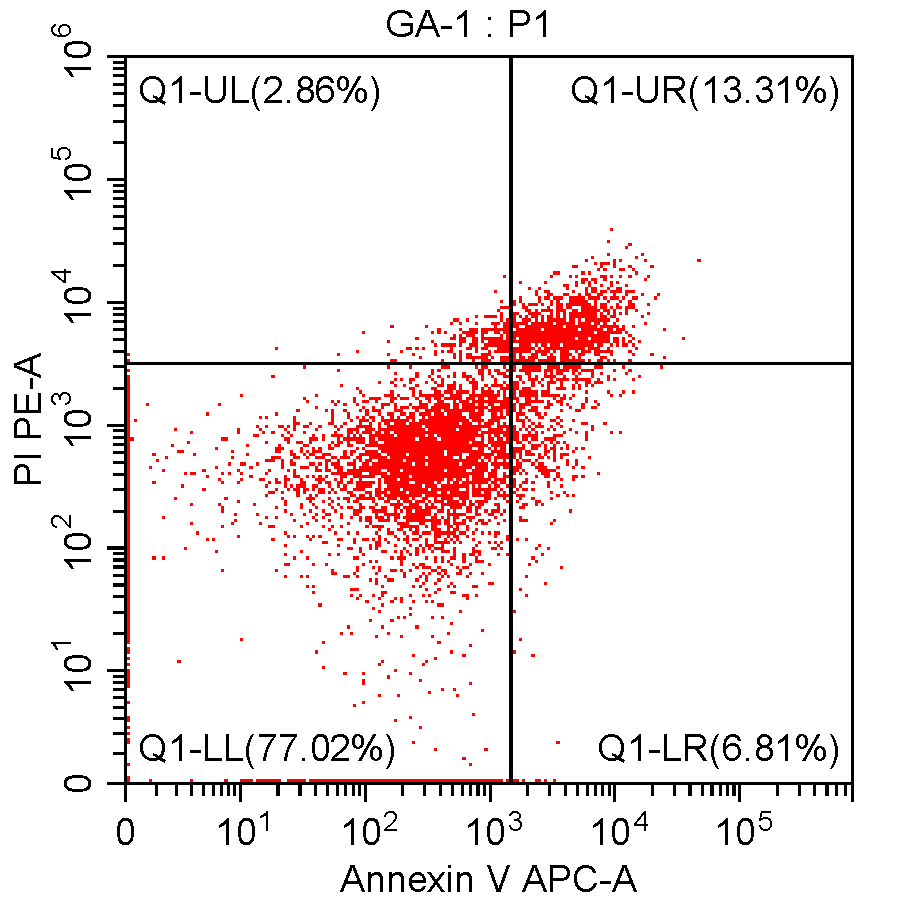

Supplement: Supplementary file 1 [file DataSheet1.zip › Data/Figure.2/Figure/flow cytometry data/GA-1_Plot2.png]

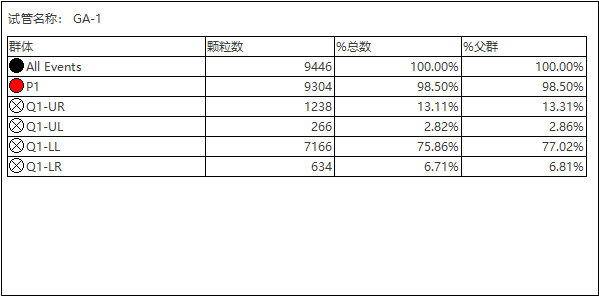

Supplement: Supplementary file 1 [file DataSheet1.zip › Data/Figure.2/Figure/flow cytometry data/GA-1_Statistics1.png]

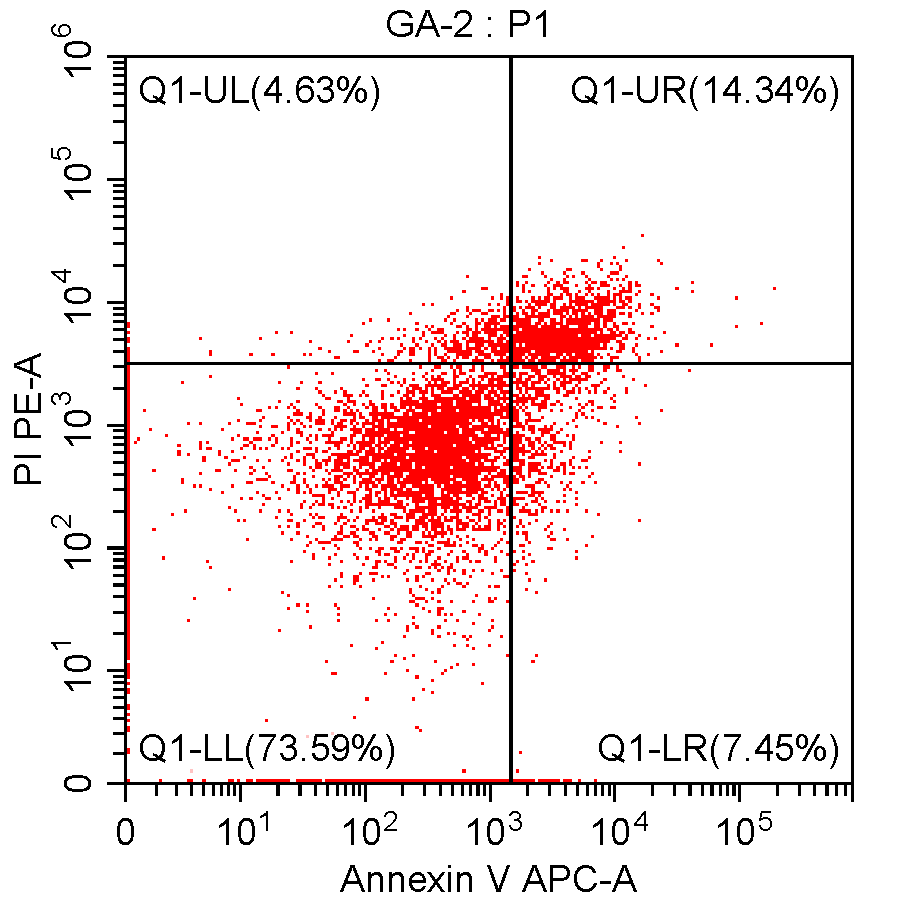

Supplement: Supplementary file 1 [file DataSheet1.zip › Data/Figure.2/Figure/flow cytometry data/GA-2_Plot2.png]

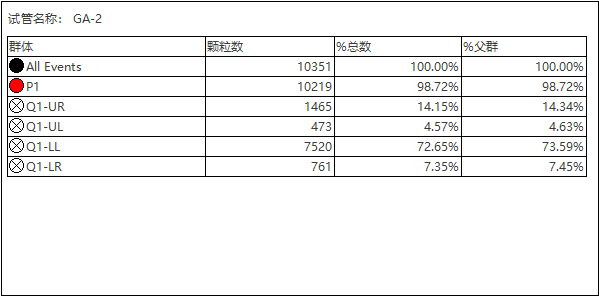

Supplement: Supplementary file 1 [file DataSheet1.zip › Data/Figure.2/Figure/flow cytometry data/GA-2_Statistics1.png]

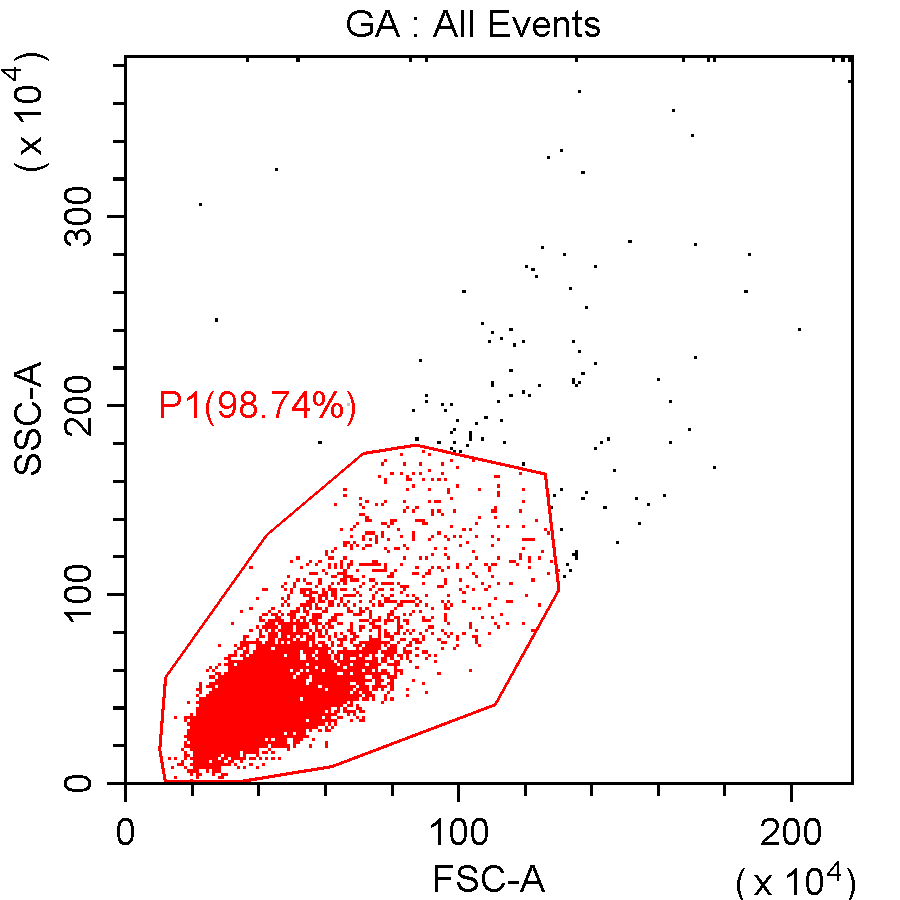

Supplement: Supplementary file 1 [file DataSheet1.zip › Data/Figure.2/Figure/flow cytometry data/GA_Plot1.png]

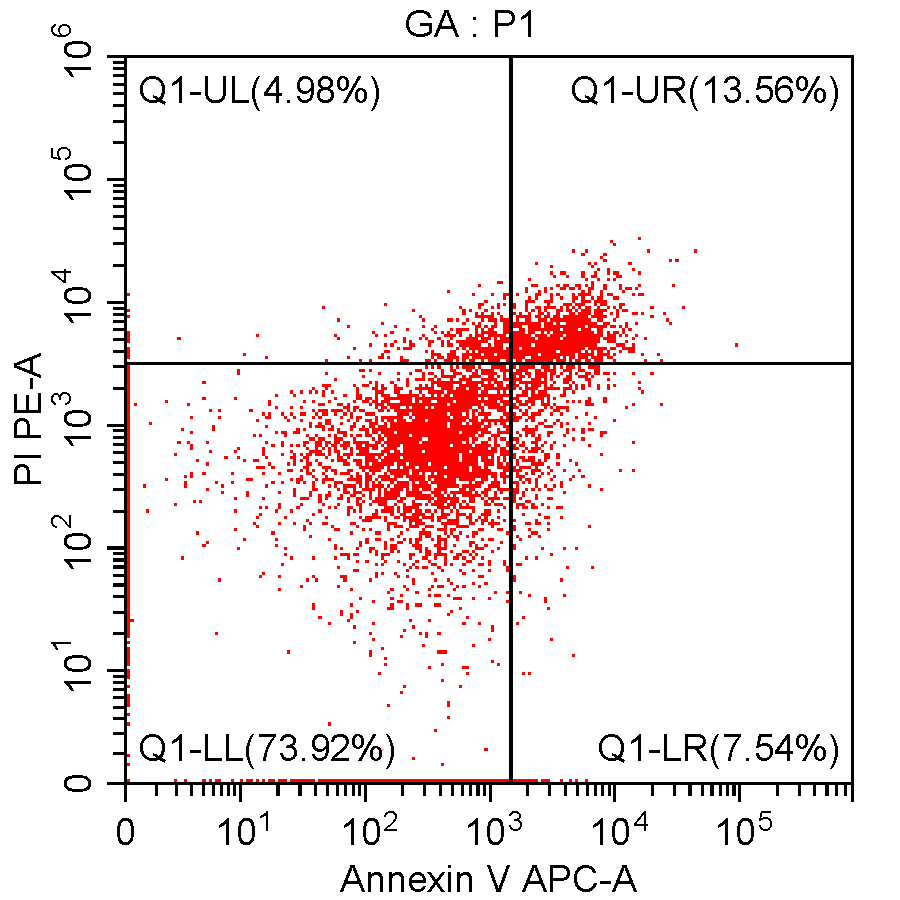

Supplement: Supplementary file 1 [file DataSheet1.zip › Data/Figure.2/Figure/flow cytometry data/GA_Plot2.png]

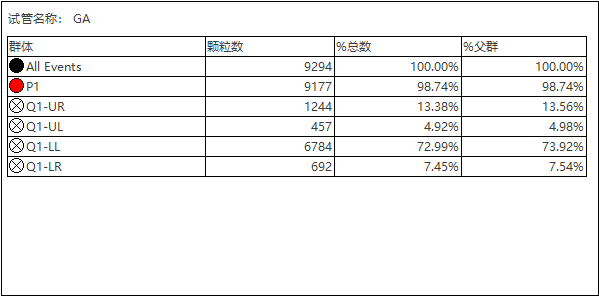

Supplement: Supplementary file 1 [file DataSheet1.zip › Data/Figure.2/Figure/flow cytometry data/GA_Statistics1.png]

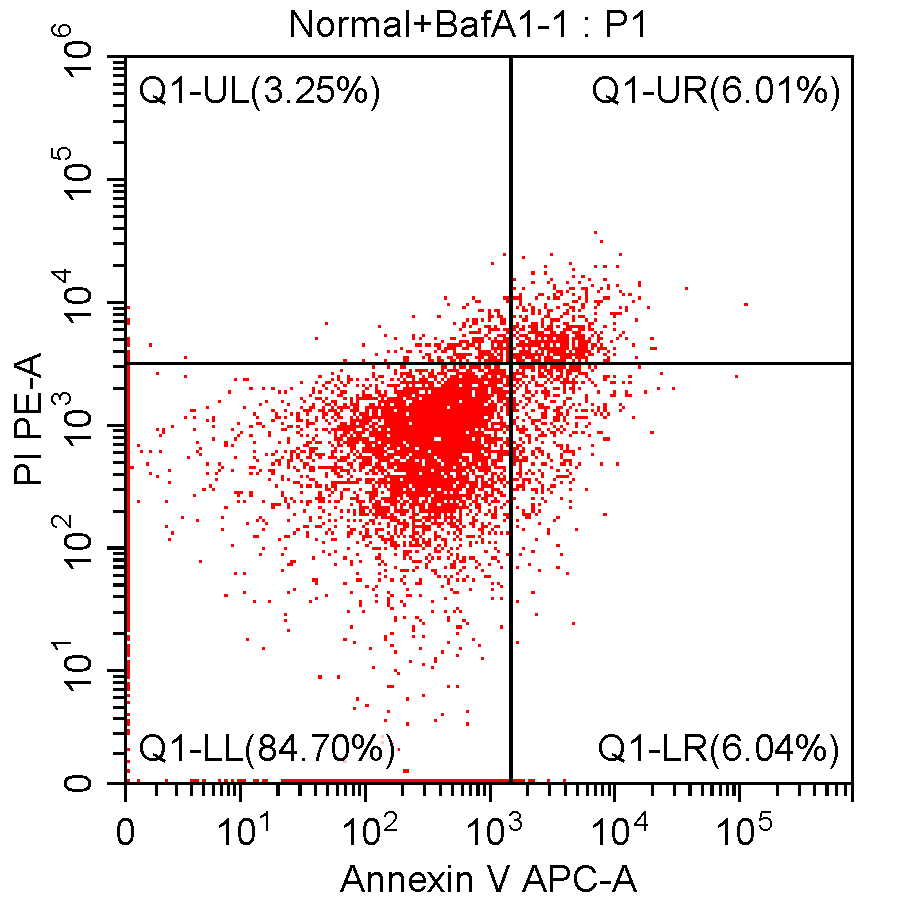

Supplement: Supplementary file 1 [file DataSheet1.zip › Data/Figure.2/Figure/flow cytometry data/Normal+BafA1-1_Plot2.png]

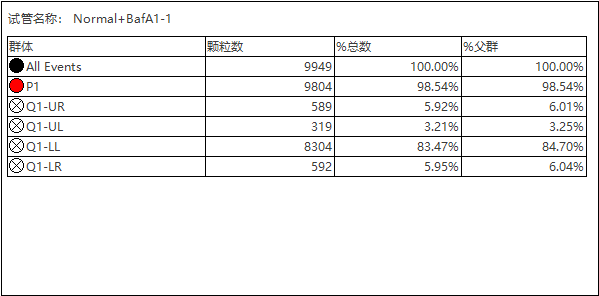

Supplement: Supplementary file 1 [file DataSheet1.zip › Data/Figure.2/Figure/flow cytometry data/Normal+BafA1-1_Statistics1.png]

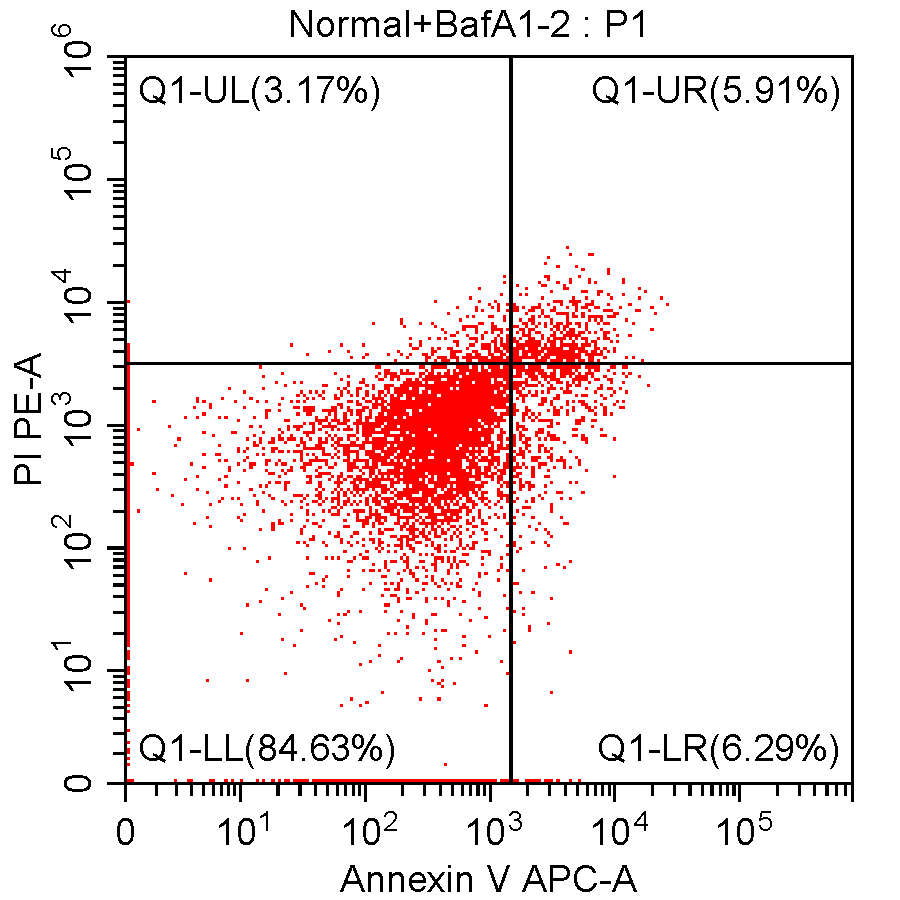

Supplement: Supplementary file 1 [file DataSheet1.zip › Data/Figure.2/Figure/flow cytometry data/Normal+BafA1-2_Plot2.png]

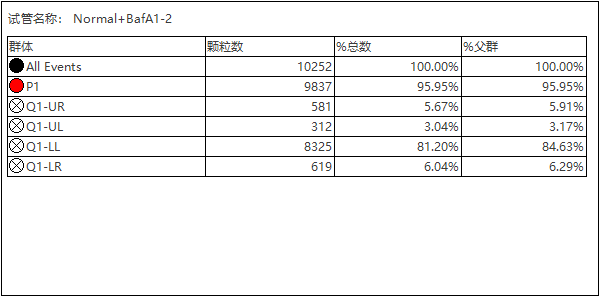

Supplement: Supplementary file 1 [file DataSheet1.zip › Data/Figure.2/Figure/flow cytometry data/Normal+BafA1-2_Statistics1.png]

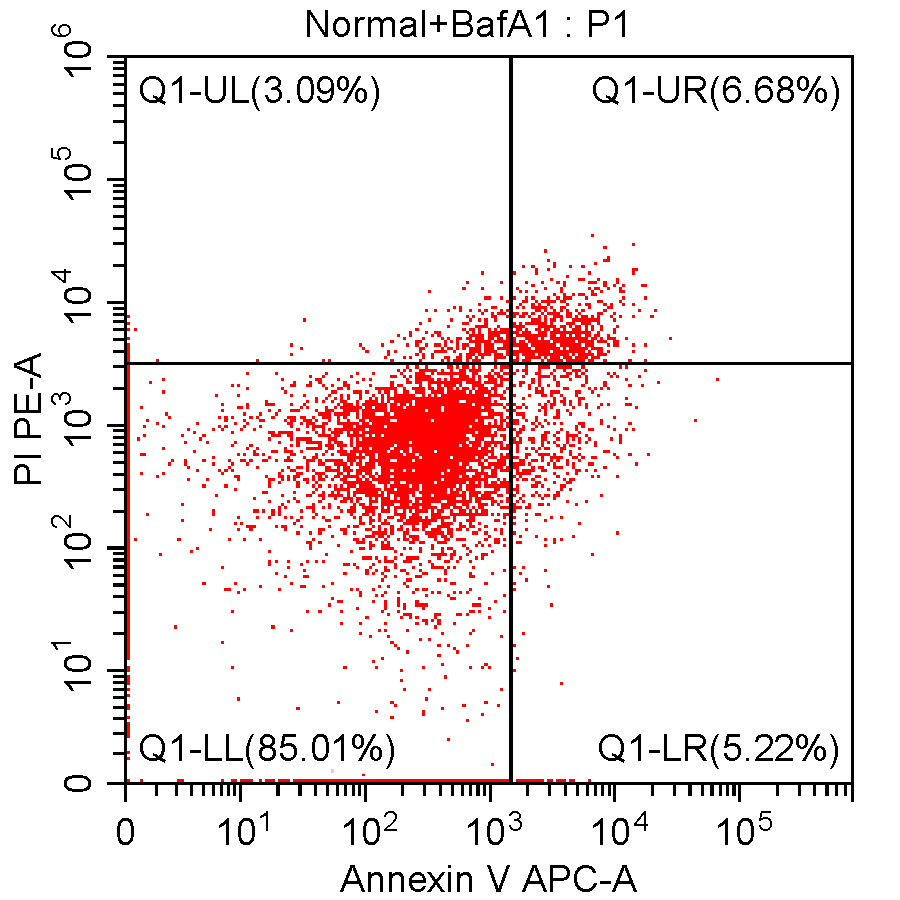

Supplement: Supplementary file 1 [file DataSheet1.zip › Data/Figure.2/Figure/flow cytometry data/Normal+BafA1_Plot2.png]

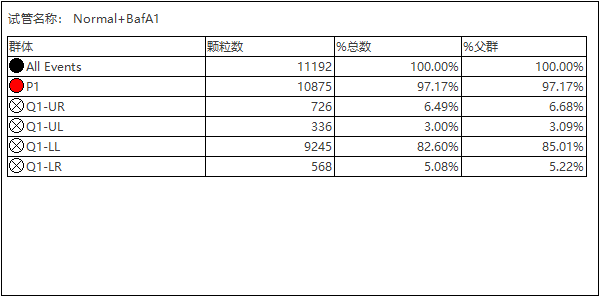

Supplement: Supplementary file 1 [file DataSheet1.zip › Data/Figure.2/Figure/flow cytometry data/Normal+BafA1_Statistics1.png]

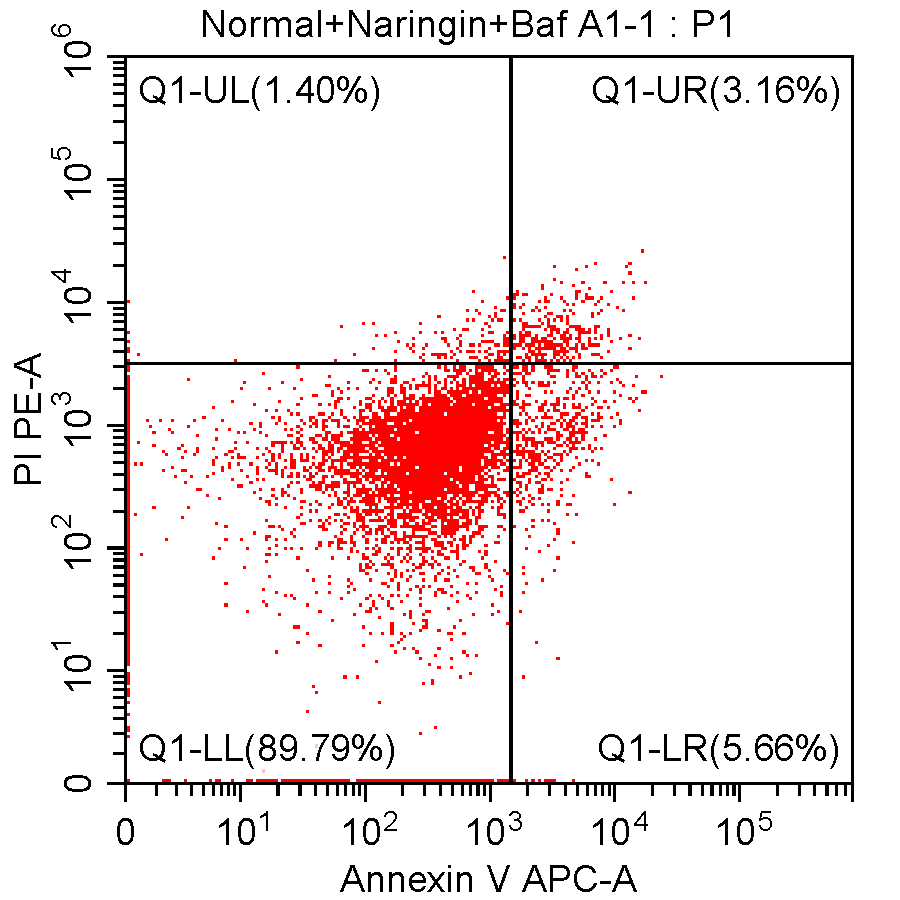

Supplement: Supplementary file 1 [file DataSheet1.zip › Data/Figure.2/Figure/flow cytometry data/Normal+Naringin+Baf A1-1_Plot2.png]

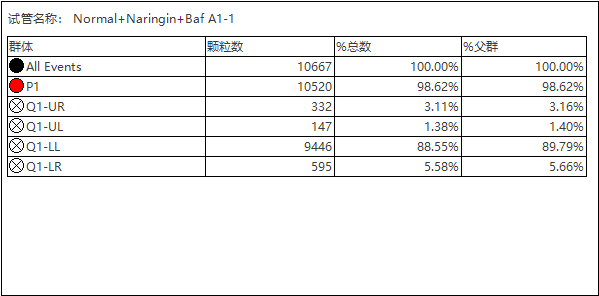

Supplement: Supplementary file 1 [file DataSheet1.zip › Data/Figure.2/Figure/flow cytometry data/Normal+Naringin+Baf A1-1_Statistics1.png]

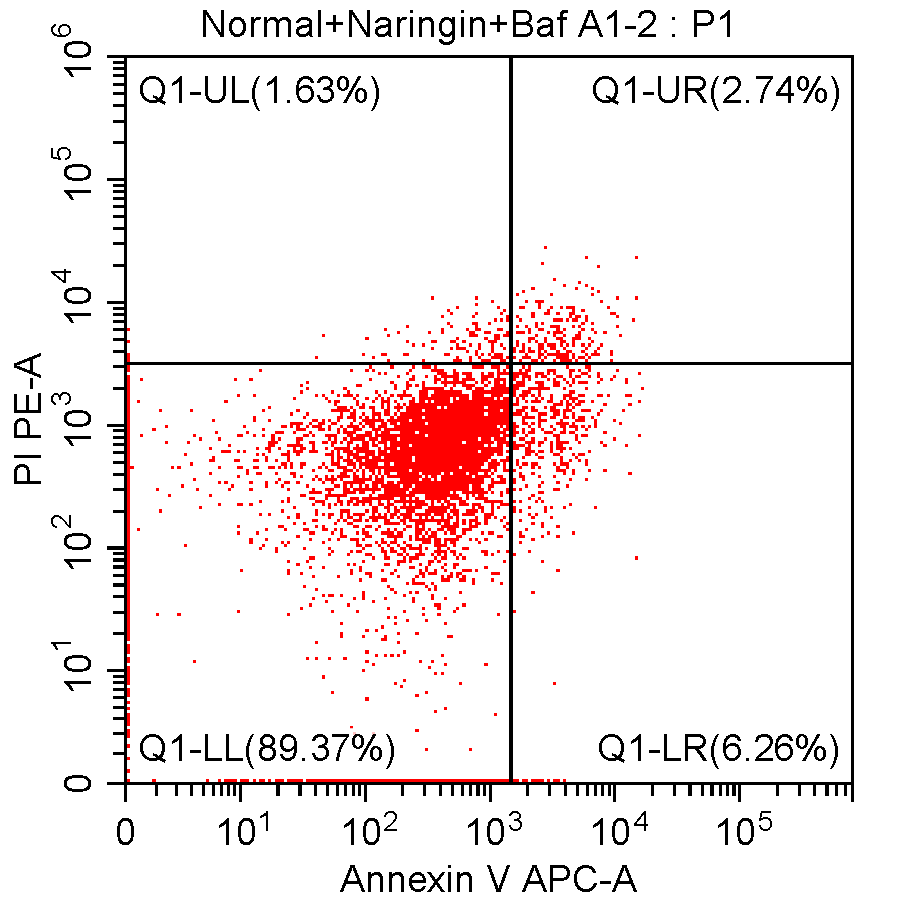

Supplement: Supplementary file 1 [file DataSheet1.zip › Data/Figure.2/Figure/flow cytometry data/Normal+Naringin+Baf A1-2_Plot2.png]

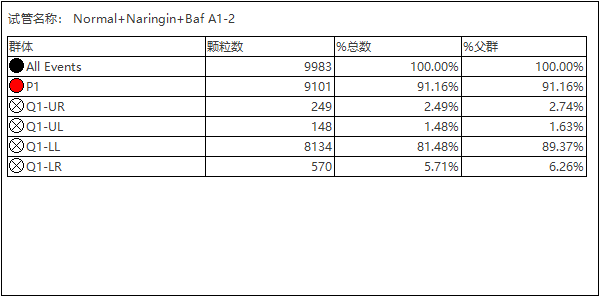

Supplement: Supplementary file 1 [file DataSheet1.zip › Data/Figure.2/Figure/flow cytometry data/Normal+Naringin+Baf A1-2_Statistics1.png]

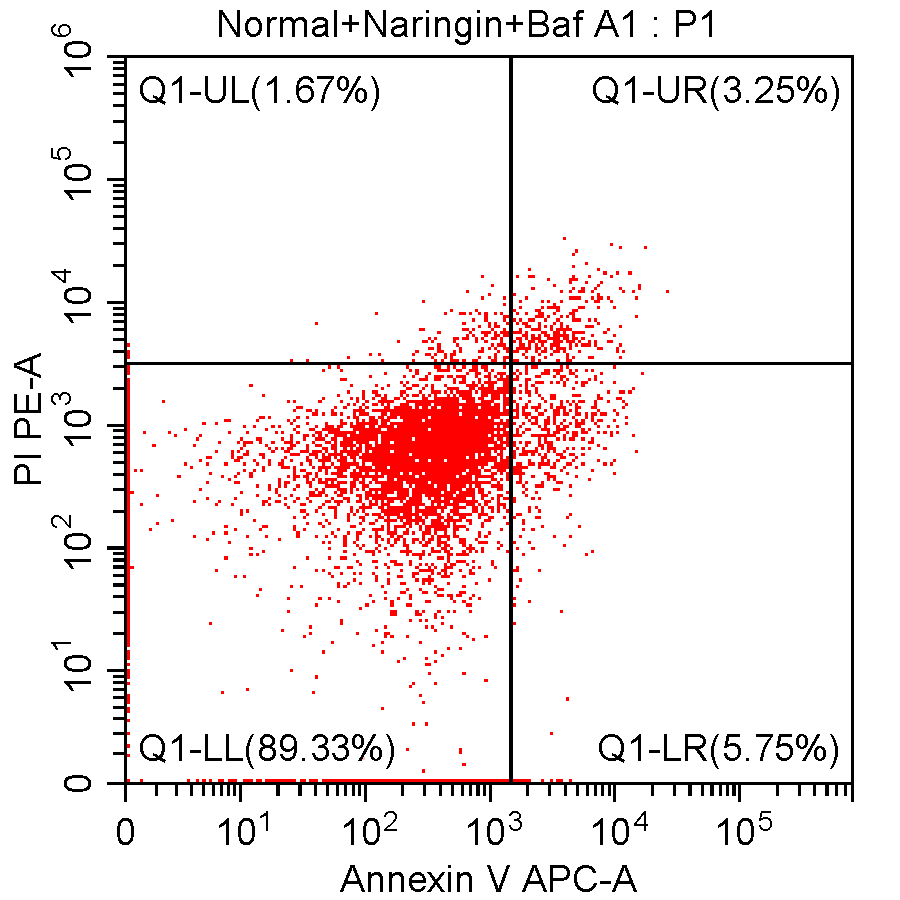

Supplement: Supplementary file 1 [file DataSheet1.zip › Data/Figure.2/Figure/flow cytometry data/Normal+Naringin+Baf A1_Plot2.png]

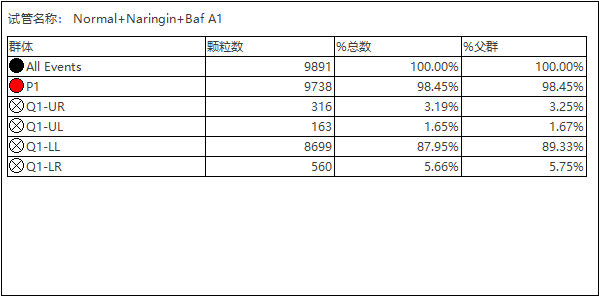

Supplement: Supplementary file 1 [file DataSheet1.zip › Data/Figure.2/Figure/flow cytometry data/Normal+Naringin+Baf A1_Statistics1.png]

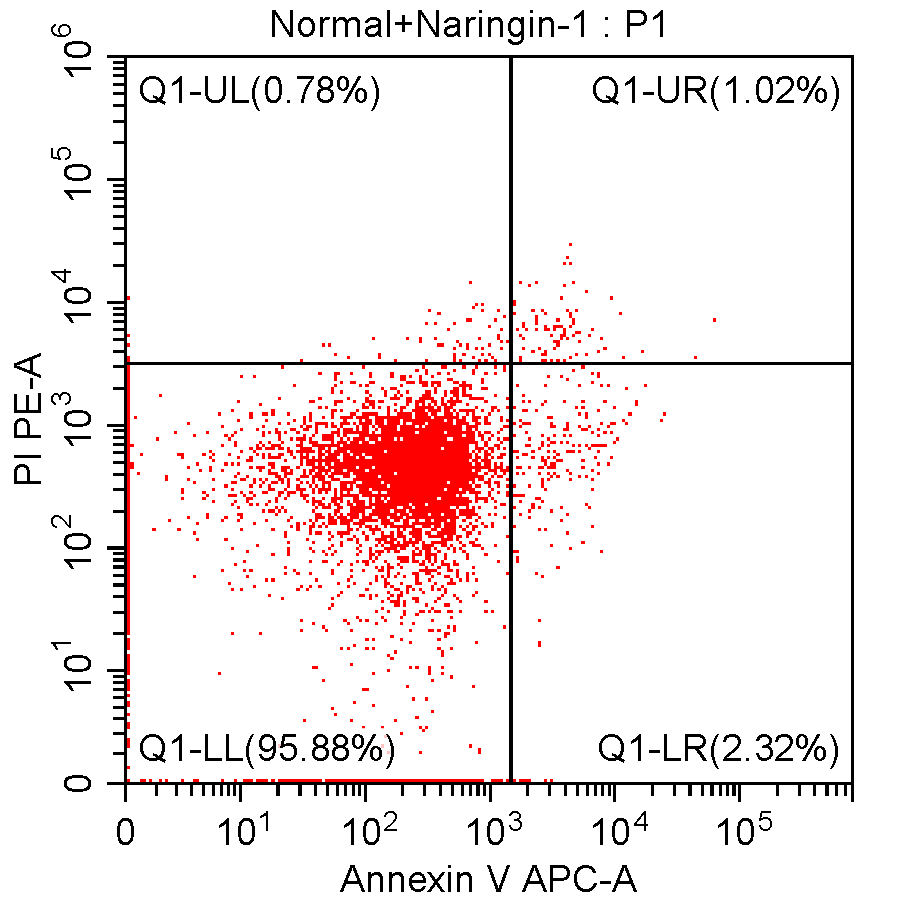

Supplement: Supplementary file 1 [file DataSheet1.zip › Data/Figure.2/Figure/flow cytometry data/Normal+Naringin-1_Plot2.png]

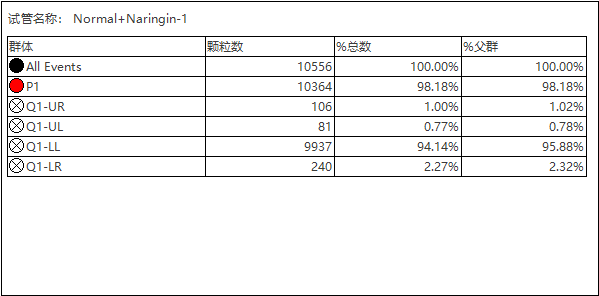

Supplement: Supplementary file 1 [file DataSheet1.zip › Data/Figure.2/Figure/flow cytometry data/Normal+Naringin-1_Statistics1.png]

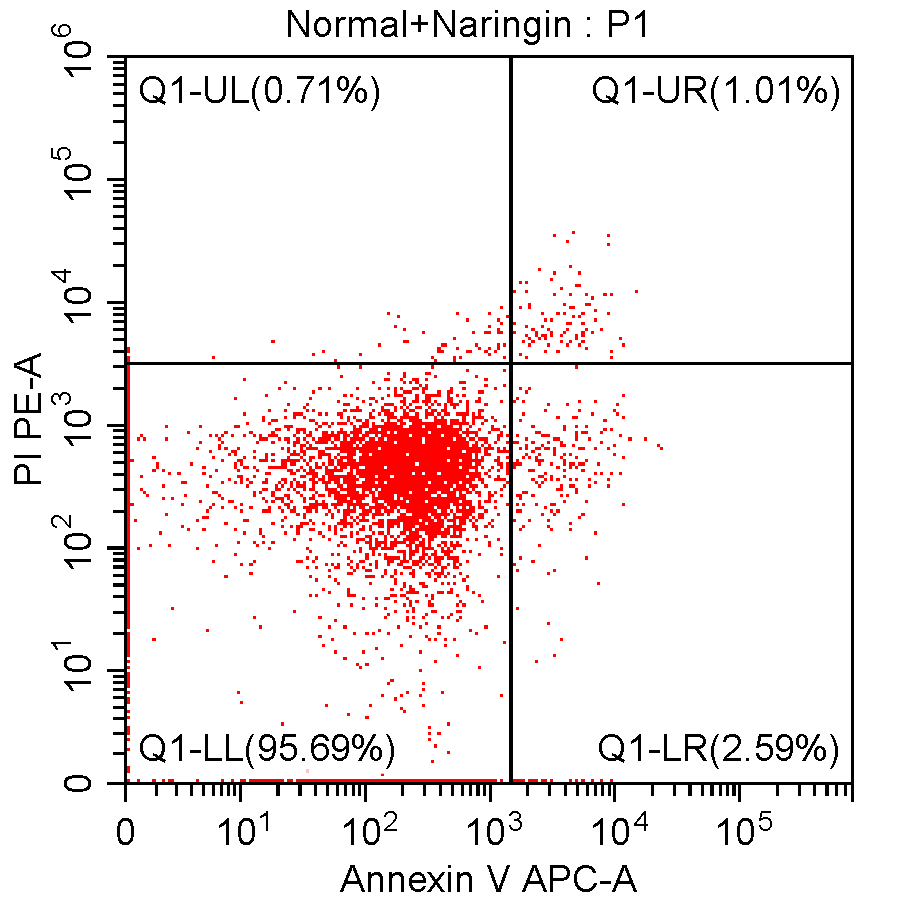

Supplement: Supplementary file 1 [file DataSheet1.zip › Data/Figure.2/Figure/flow cytometry data/Normal+Naringin_Plot2.png]

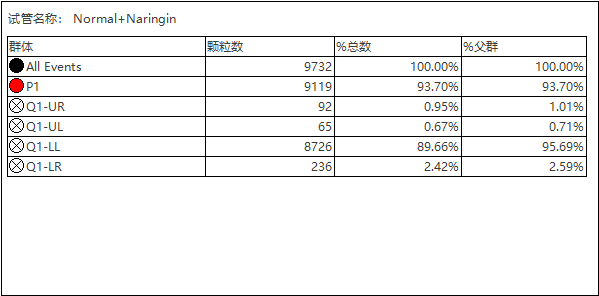

Supplement: Supplementary file 1 [file DataSheet1.zip › Data/Figure.2/Figure/flow cytometry data/Normal+Naringin_Statistics1.png]

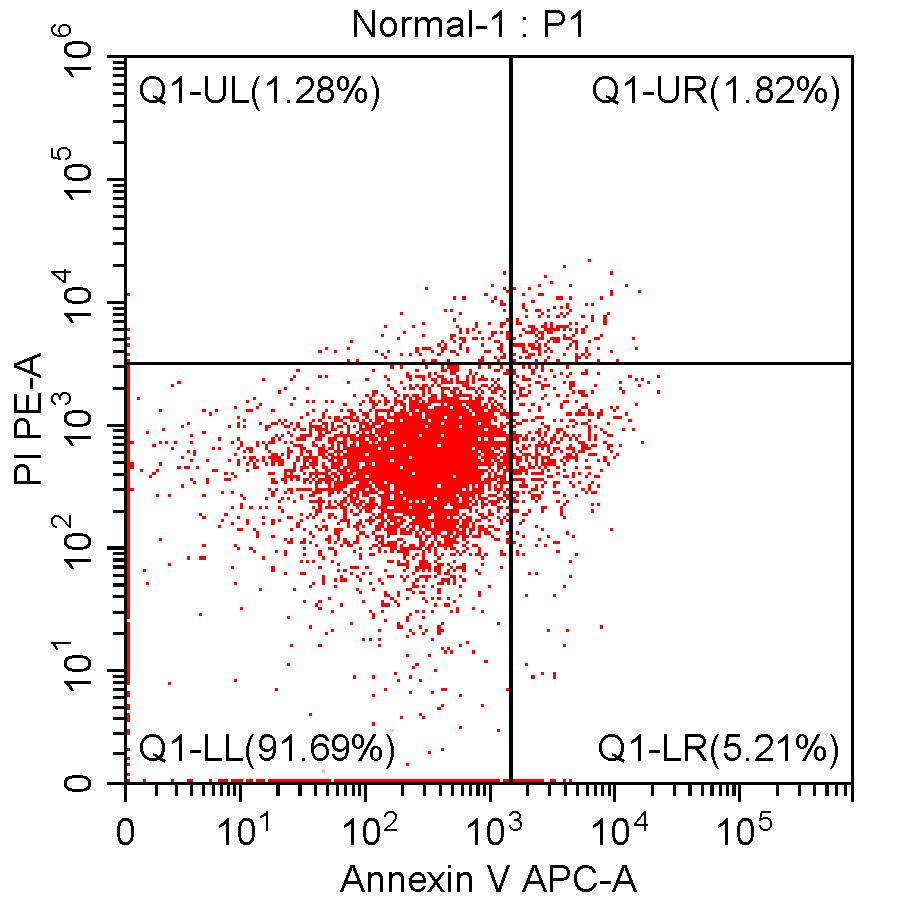

Supplement: Supplementary file 1 [file DataSheet1.zip › Data/Figure.2/Figure/flow cytometry data/Normal-1_Plot2.png]

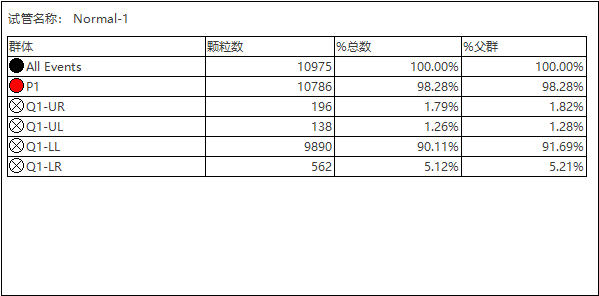

Supplement: Supplementary file 1 [file DataSheet1.zip › Data/Figure.2/Figure/flow cytometry data/Normal-1_Statistics1.png]

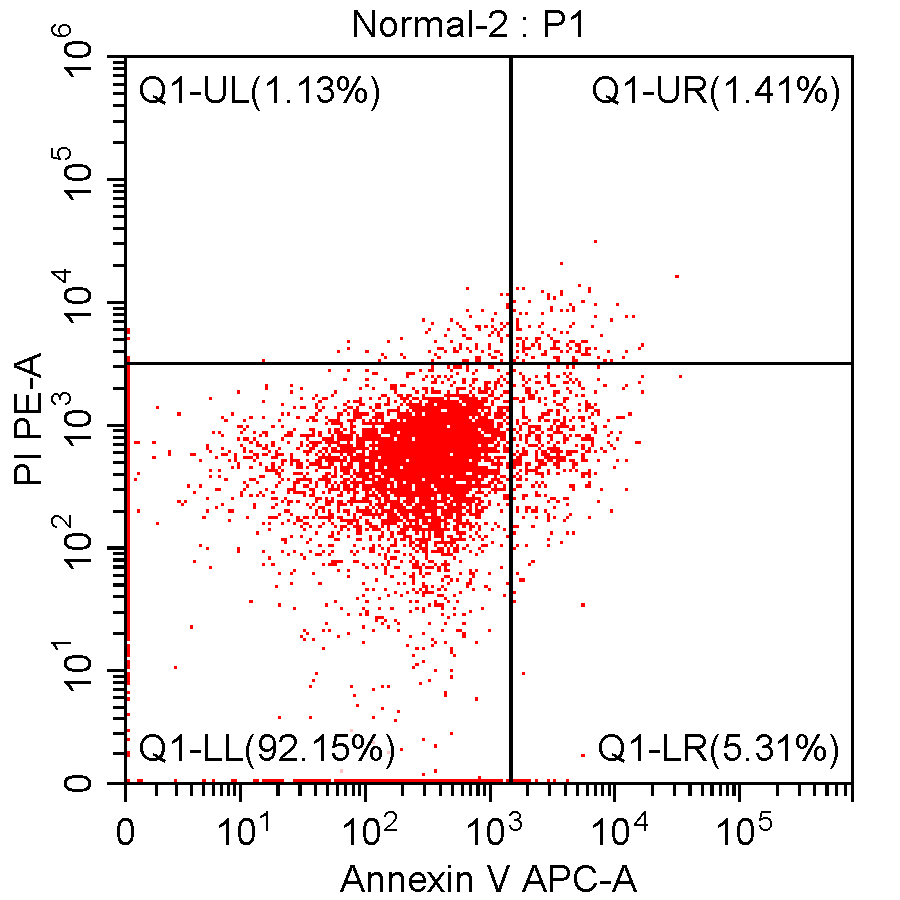

Supplement: Supplementary file 1 [file DataSheet1.zip › Data/Figure.2/Figure/flow cytometry data/Normal-2_Plot2.png]

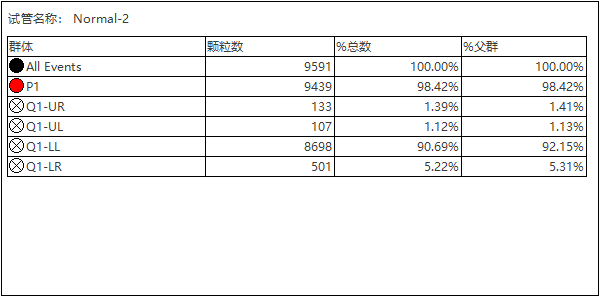

Supplement: Supplementary file 1 [file DataSheet1.zip › Data/Figure.2/Figure/flow cytometry data/Normal-2_Statistics1.png]

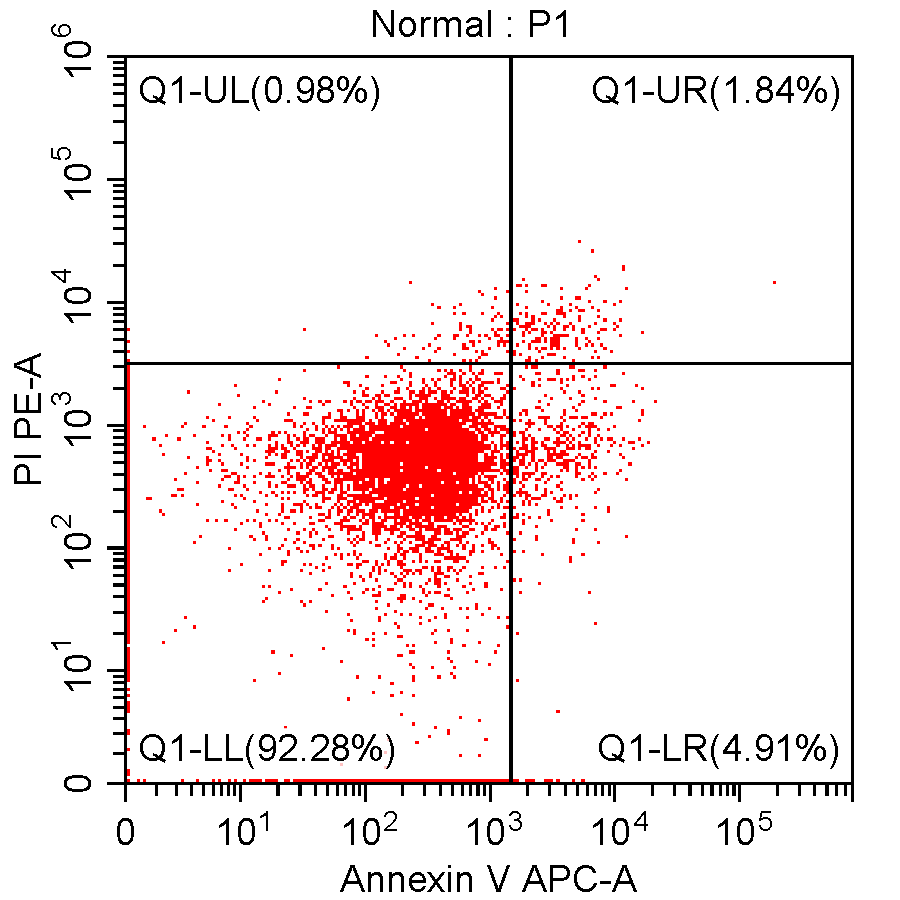

Supplement: Supplementary file 1 [file DataSheet1.zip › Data/Figure.2/Figure/flow cytometry data/Normal_Plot2.png]

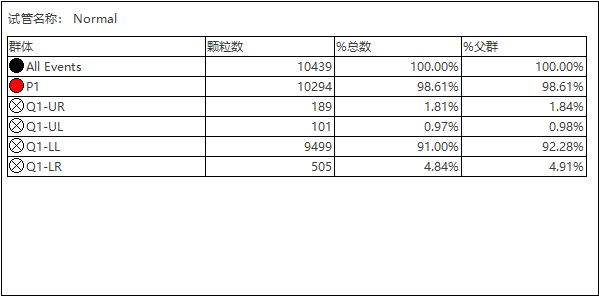

Supplement: Supplementary file 1 [file DataSheet1.zip › Data/Figure.2/Figure/flow cytometry data/Normal_Statistics1.png]

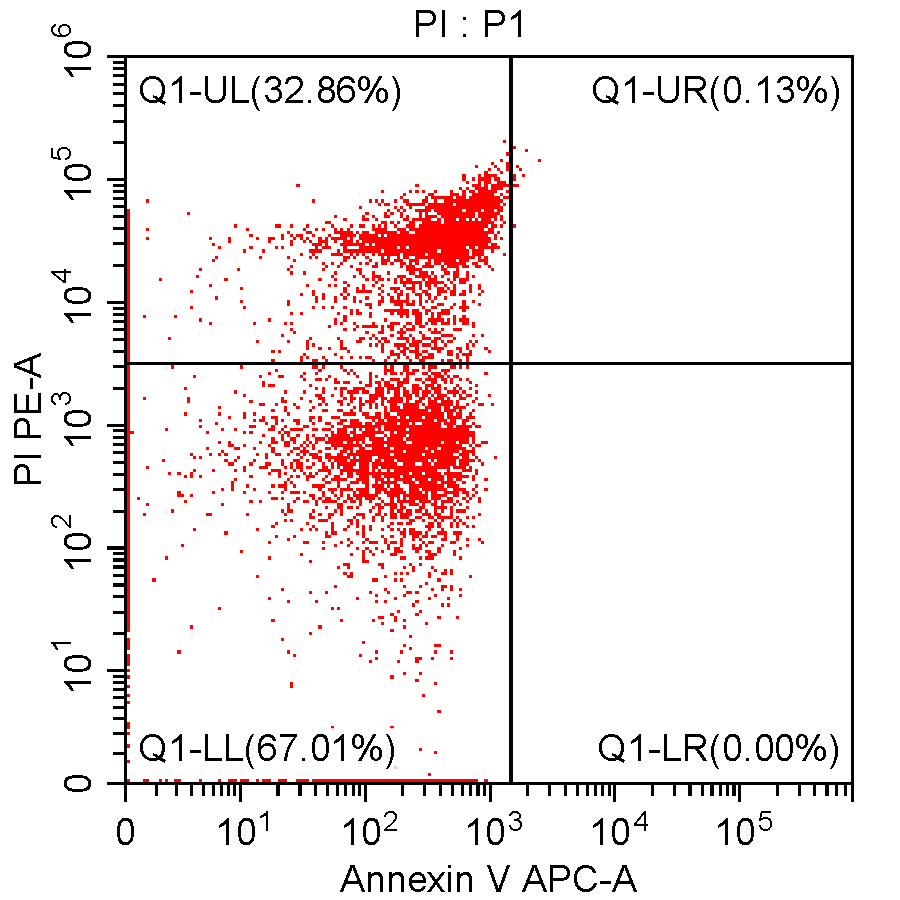

Supplement: Supplementary file 1 [file DataSheet1.zip › Data/Figure.2/Figure/flow cytometry data/PI_Plot2.png]

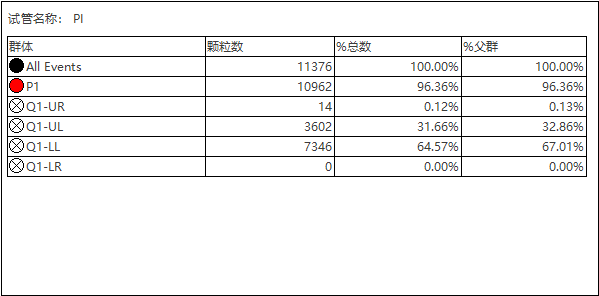

Supplement: Supplementary file 1 [file DataSheet1.zip › Data/Figure.2/Figure/flow cytometry data/PI_Statistics1.png]

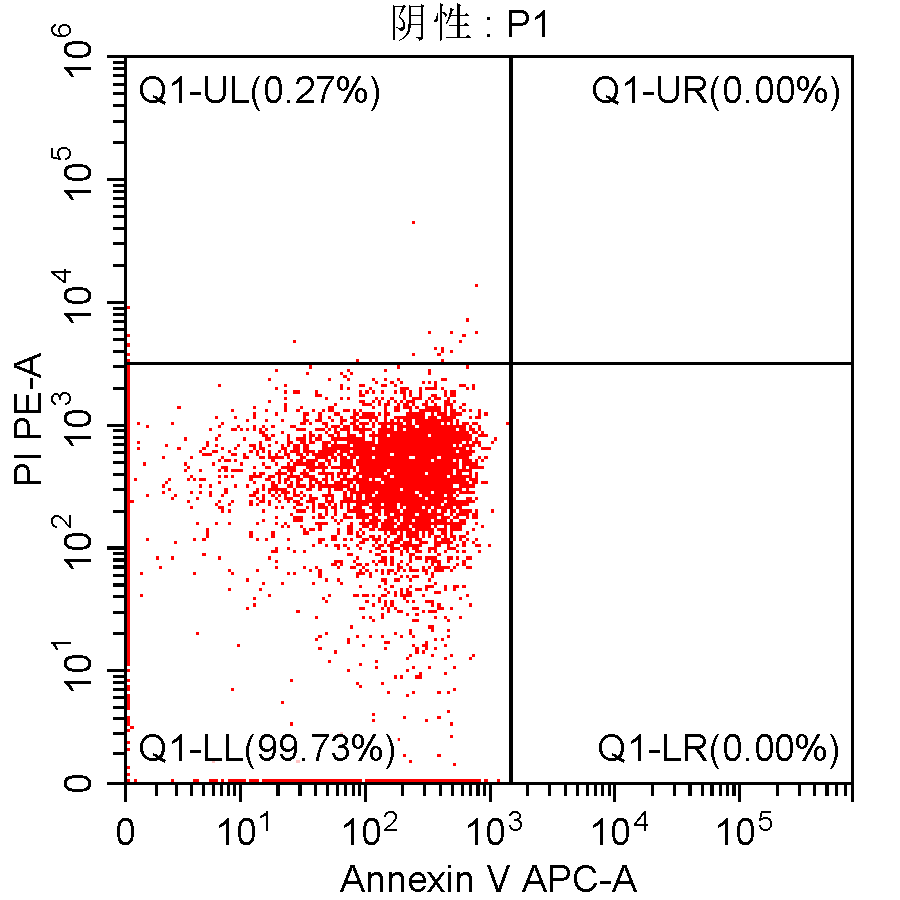

Supplement: Supplementary file 1 [file DataSheet1.zip › Data/Figure.2/Figure/flow cytometry data/阴性_Plot2.png]

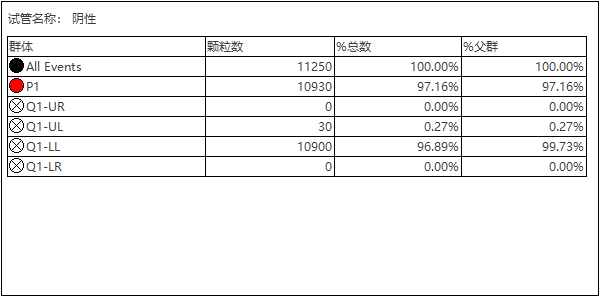

Supplement: Supplementary file 1 [file DataSheet1.zip › Data/Figure.2/Figure/flow cytometry data/阴性_Statistics1.png]

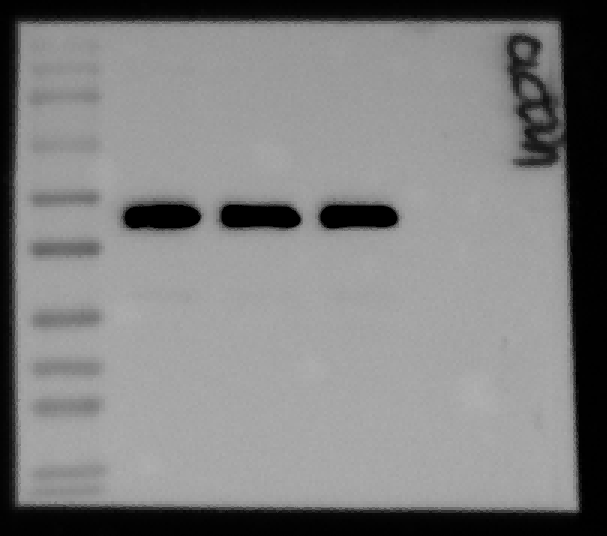

Supplement: Supplementary file 1 [file DataSheet1.zip › Data/Figure.3/Figure/Western Blot/Figure.3 1/actin 1.png]

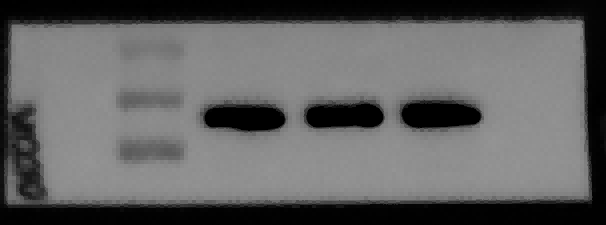

Supplement: Supplementary file 1 [file DataSheet1.zip › Data/Figure.3/Figure/Western Blot/Figure.3 1/actin 2.png]

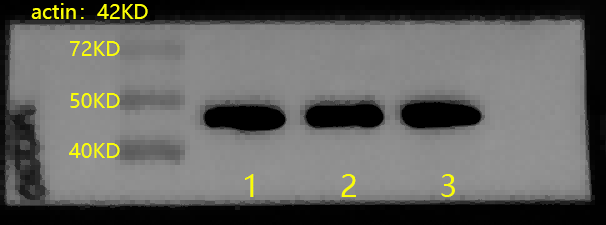

Supplement: Supplementary file 1 [file DataSheet1.zip › Data/Figure.3/Figure/Western Blot/Figure.3 1/actin 2标.png]

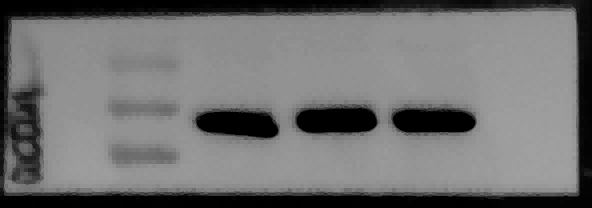

Supplement: Supplementary file 1 [file DataSheet1.zip › Data/Figure.3/Figure/Western Blot/Figure.3 1/actin 3.png]

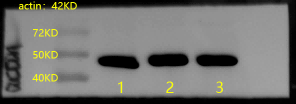

Supplement: Supplementary file 1 [file DataSheet1.zip › Data/Figure.3/Figure/Western Blot/Figure.3 1/actin 3标.png]

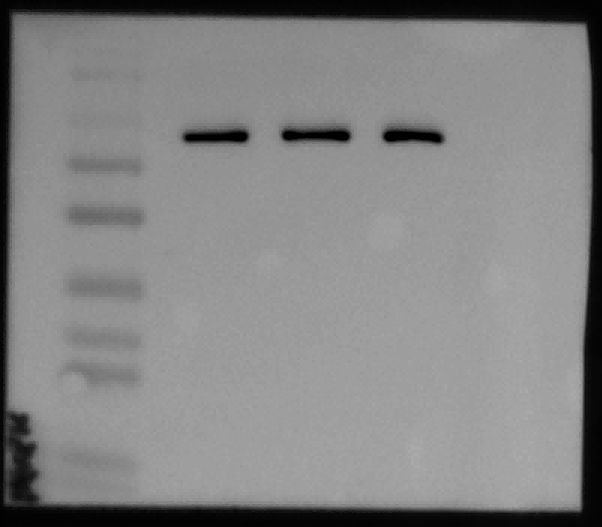

Supplement: Supplementary file 1 [file DataSheet1.zip › Data/Figure.3/Figure/Western Blot/Figure.3 1/AMPK 1.png]

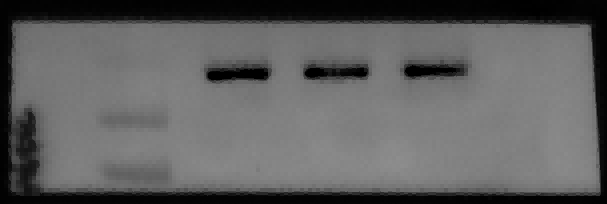

Supplement: Supplementary file 1 [file DataSheet1.zip › Data/Figure.3/Figure/Western Blot/Figure.3 1/AMPK 2.png]

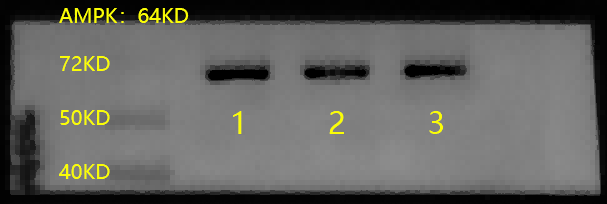

Supplement: Supplementary file 1 [file DataSheet1.zip › Data/Figure.3/Figure/Western Blot/Figure.3 1/AMPK 2标.png]

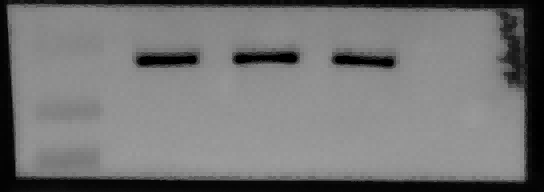

Supplement: Supplementary file 1 [file DataSheet1.zip › Data/Figure.3/Figure/Western Blot/Figure.3 1/AMPK 3.png]

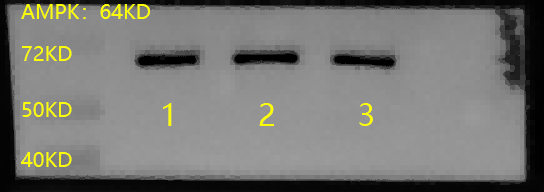

Supplement: Supplementary file 1 [file DataSheet1.zip › Data/Figure.3/Figure/Western Blot/Figure.3 1/AMPK 3标.png]

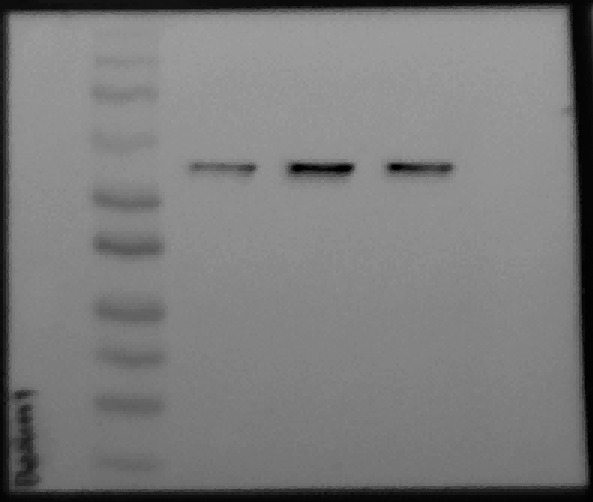

Supplement: Supplementary file 1 [file DataSheet1.zip › Data/Figure.3/Figure/Western Blot/Figure.3 1/beclin1 1.png]

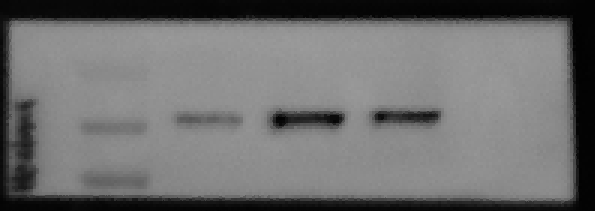

Supplement: Supplementary file 1 [file DataSheet1.zip › Data/Figure.3/Figure/Western Blot/Figure.3 1/beclin1 2.png]

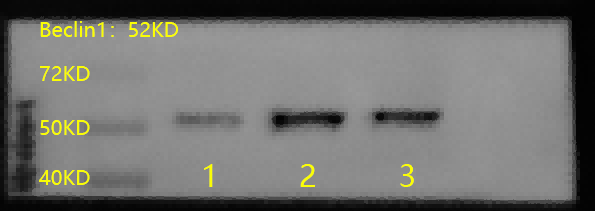

Supplement: Supplementary file 1 [file DataSheet1.zip › Data/Figure.3/Figure/Western Blot/Figure.3 1/beclin1 2标.png]

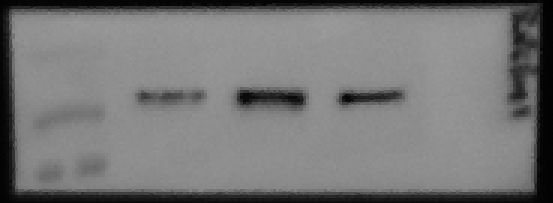

Supplement: Supplementary file 1 [file DataSheet1.zip › Data/Figure.3/Figure/Western Blot/Figure.3 1/beclin1 3.png]

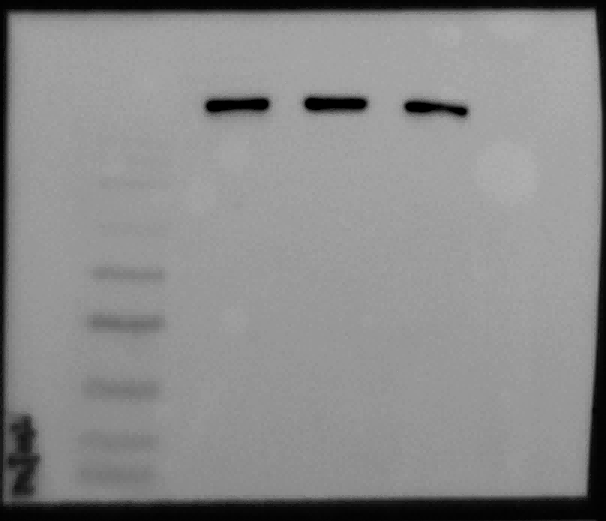

Supplement: Supplementary file 1 [file DataSheet1.zip › Data/Figure.3/Figure/Western Blot/Figure.3 1/MTOR 1.png]

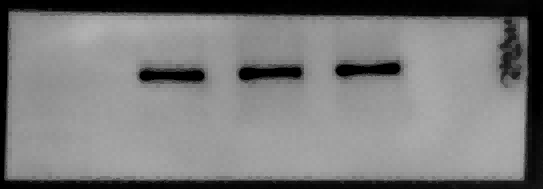

Supplement: Supplementary file 1 [file DataSheet1.zip › Data/Figure.3/Figure/Western Blot/Figure.3 1/MTOR 2.png]

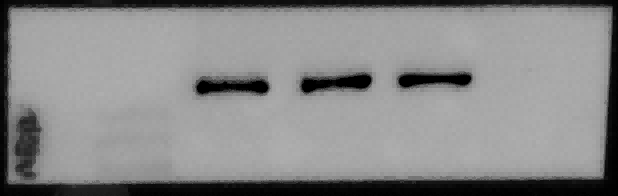

Supplement: Supplementary file 1 [file DataSheet1.zip › Data/Figure.3/Figure/Western Blot/Figure.3 1/MTOR 3.png]

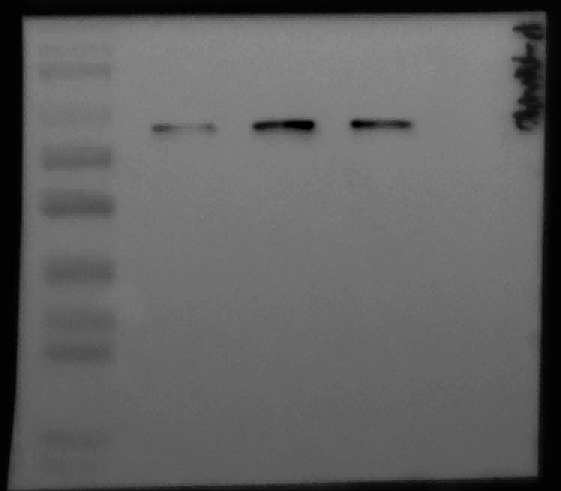

Supplement: Supplementary file 1 [file DataSheet1.zip › Data/Figure.3/Figure/Western Blot/Figure.3 1/P-AMPK 1.png]

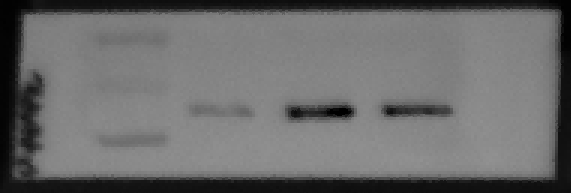

Supplement: Supplementary file 1 [file DataSheet1.zip › Data/Figure.3/Figure/Western Blot/Figure.3 1/P-AMPK 2.png]

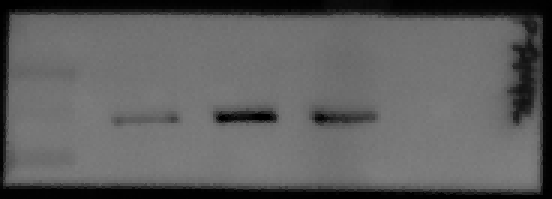

Supplement: Supplementary file 1 [file DataSheet1.zip › Data/Figure.3/Figure/Western Blot/Figure.3 1/P-AMPK 3.png]

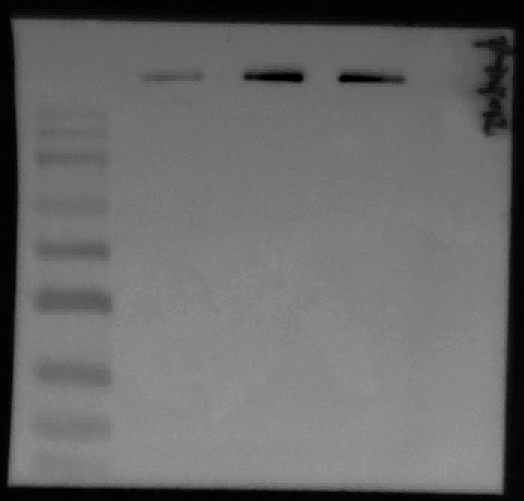

Supplement: Supplementary file 1 [file DataSheet1.zip › Data/Figure.3/Figure/Western Blot/Figure.3 1/P-MTOR 1.png]

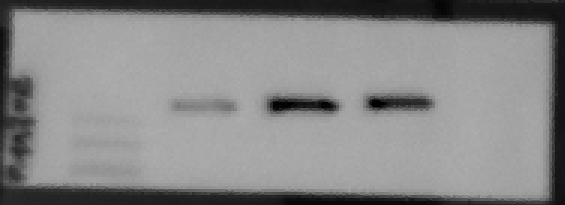

Supplement: Supplementary file 1 [file DataSheet1.zip › Data/Figure.3/Figure/Western Blot/Figure.3 1/P-MTOR 2.png]

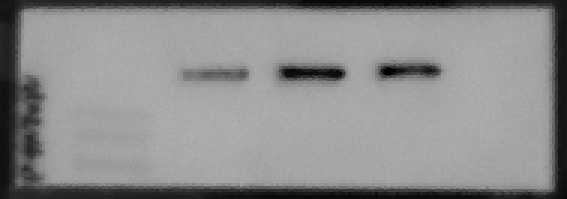

Supplement: Supplementary file 1 [file DataSheet1.zip › Data/Figure.3/Figure/Western Blot/Figure.3 1/P-MTOR 3.png]

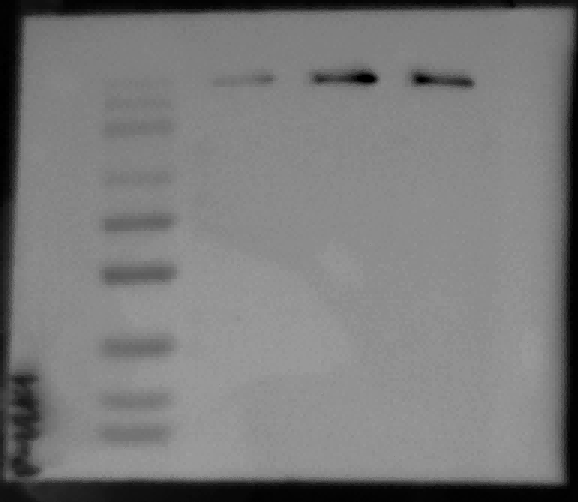

Supplement: Supplementary file 1 [file DataSheet1.zip › Data/Figure.3/Figure/Western Blot/Figure.3 1/P-ULK1 1.png]

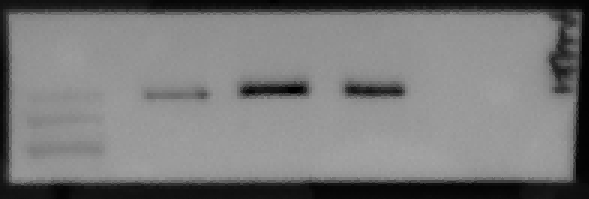

Supplement: Supplementary file 1 [file DataSheet1.zip › Data/Figure.3/Figure/Western Blot/Figure.3 1/P-ULK1 2.png]

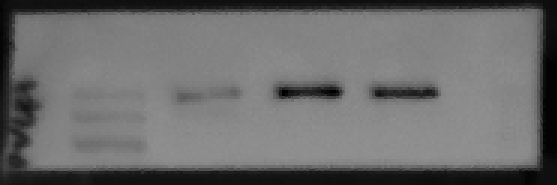

Supplement: Supplementary file 1 [file DataSheet1.zip › Data/Figure.3/Figure/Western Blot/Figure.3 1/P-ULK1 3.png]

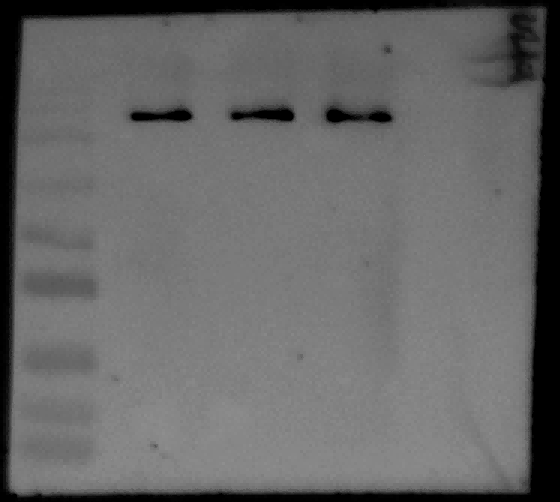

Supplement: Supplementary file 1 [file DataSheet1.zip › Data/Figure.3/Figure/Western Blot/Figure.3 1/ULK1 1.png]

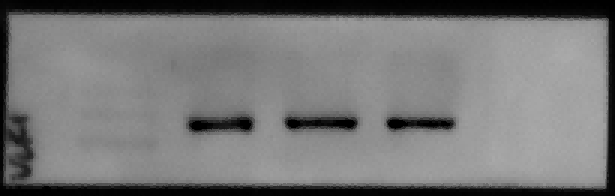

Supplement: Supplementary file 1 [file DataSheet1.zip › Data/Figure.3/Figure/Western Blot/Figure.3 1/ULK1 2.png]

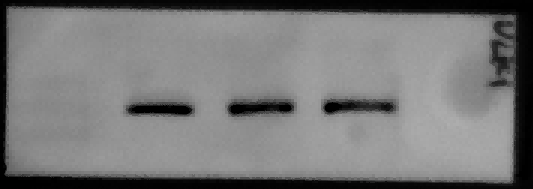

Supplement: Supplementary file 1 [file DataSheet1.zip › Data/Figure.3/Figure/Western Blot/Figure.3 1/ULK1 3.png]

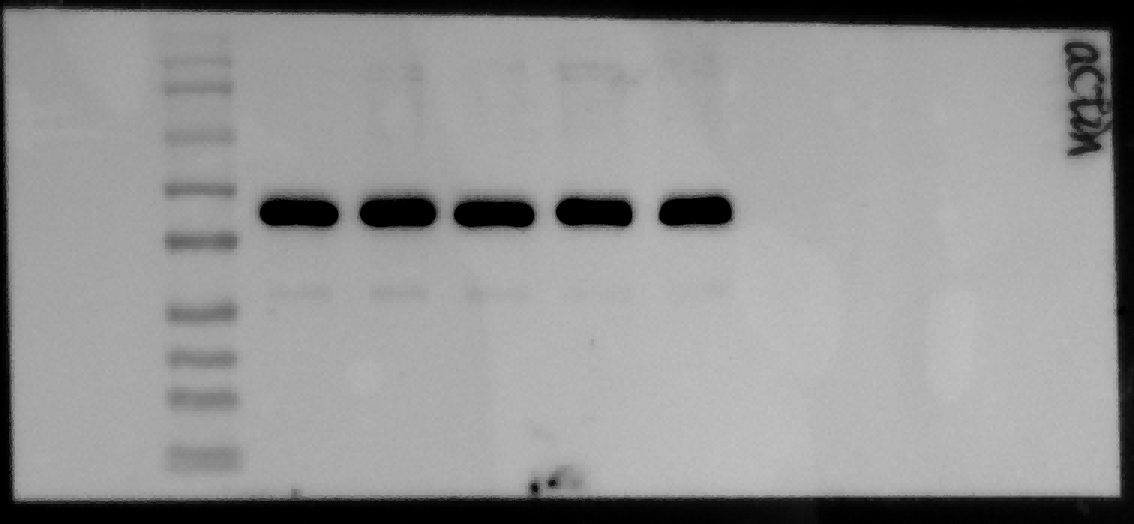

Supplement: Supplementary file 1 [file DataSheet1.zip › Data/Figure.3/Figure/Western Blot/Figure.3 2/actin 1.png]

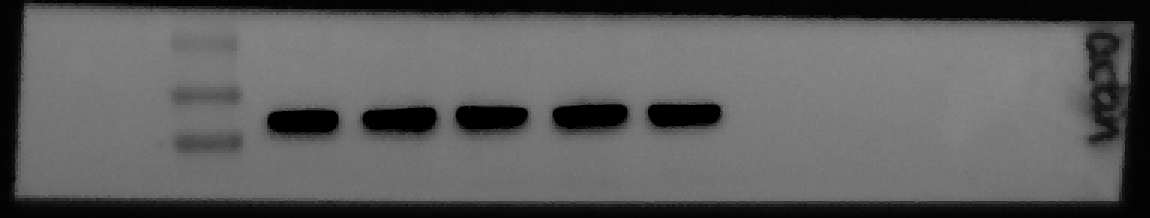

Supplement: Supplementary file 1 [file DataSheet1.zip › Data/Figure.3/Figure/Western Blot/Figure.3 2/actin 2.png]

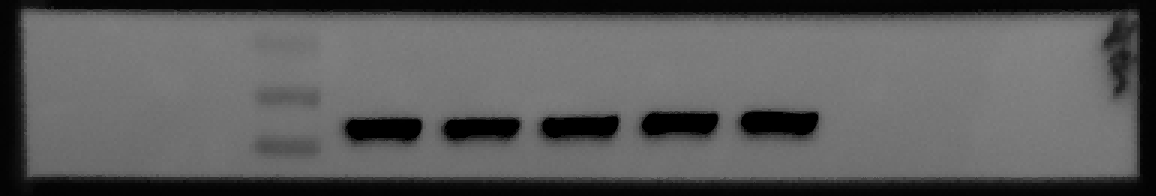

Supplement: Supplementary file 1 [file DataSheet1.zip › Data/Figure.3/Figure/Western Blot/Figure.3 2/actin 3.png]

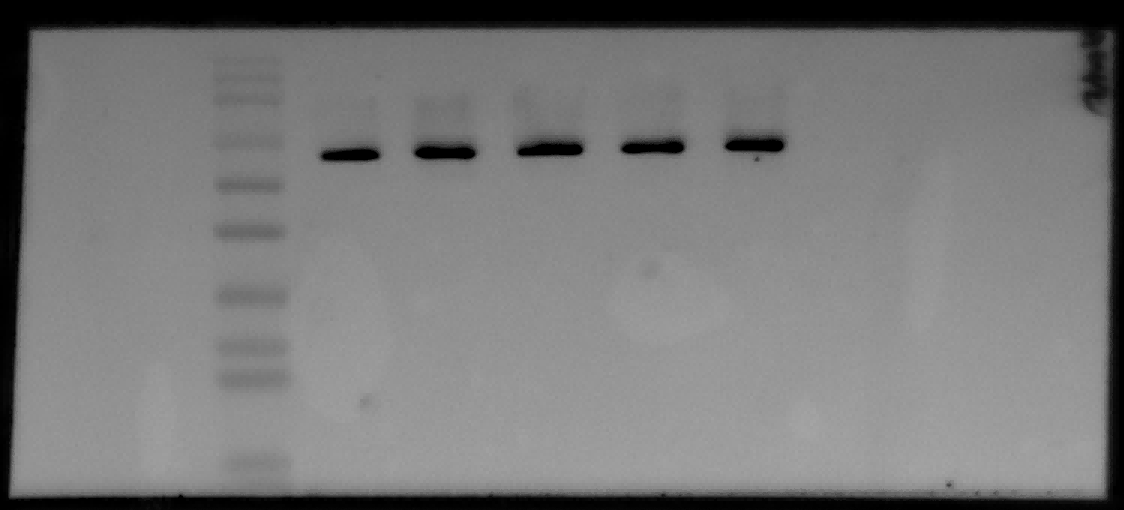

Supplement: Supplementary file 1 [file DataSheet1.zip › Data/Figure.3/Figure/Western Blot/Figure.3 2/AMPK 1.png]

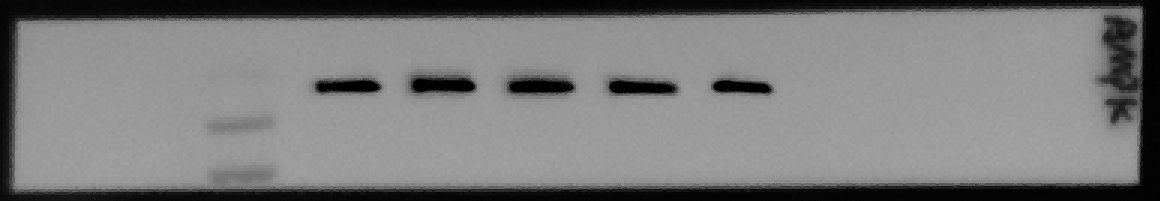

Supplement: Supplementary file 1 [file DataSheet1.zip › Data/Figure.3/Figure/Western Blot/Figure.3 2/AMPK 2.png]

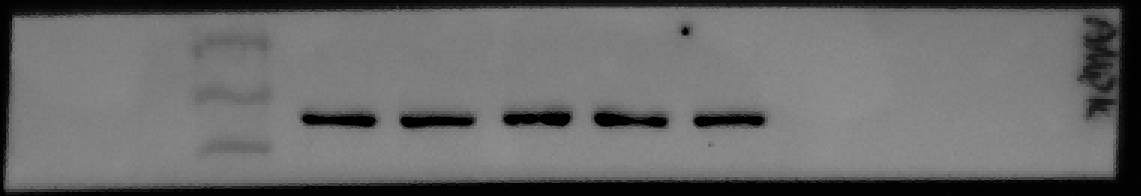

Supplement: Supplementary file 1 [file DataSheet1.zip › Data/Figure.3/Figure/Western Blot/Figure.3 2/AMPK 3.png]

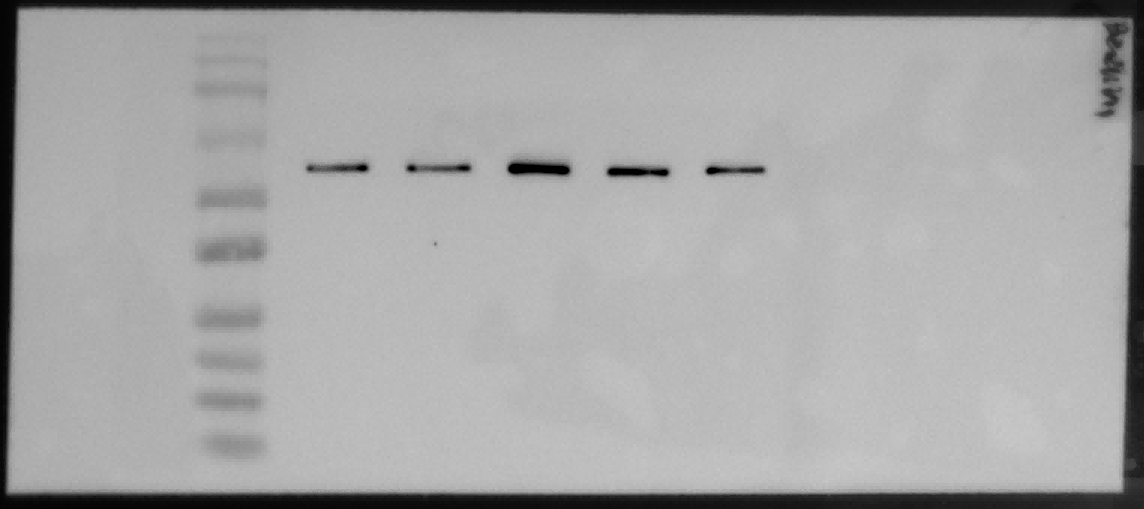

Supplement: Supplementary file 1 [file DataSheet1.zip › Data/Figure.3/Figure/Western Blot/Figure.3 2/beclin1 1.png]

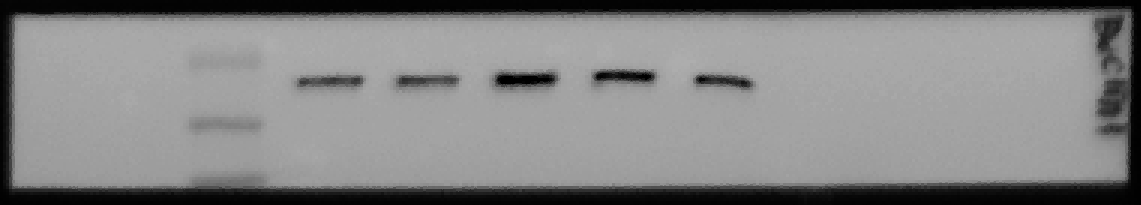

Supplement: Supplementary file 1 [file DataSheet1.zip › Data/Figure.3/Figure/Western Blot/Figure.3 2/beclin1 2.png]

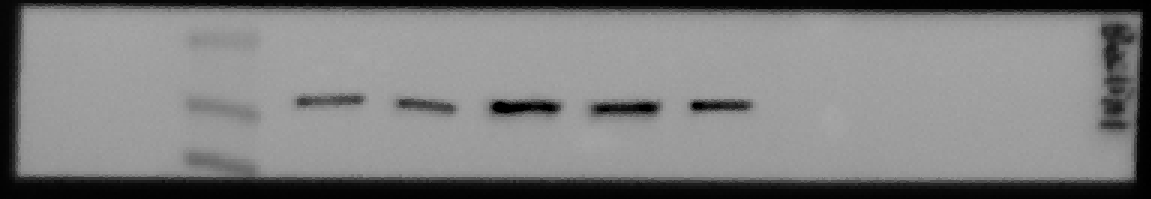

Supplement: Supplementary file 1 [file DataSheet1.zip › Data/Figure.3/Figure/Western Blot/Figure.3 2/beclin1 3.png]

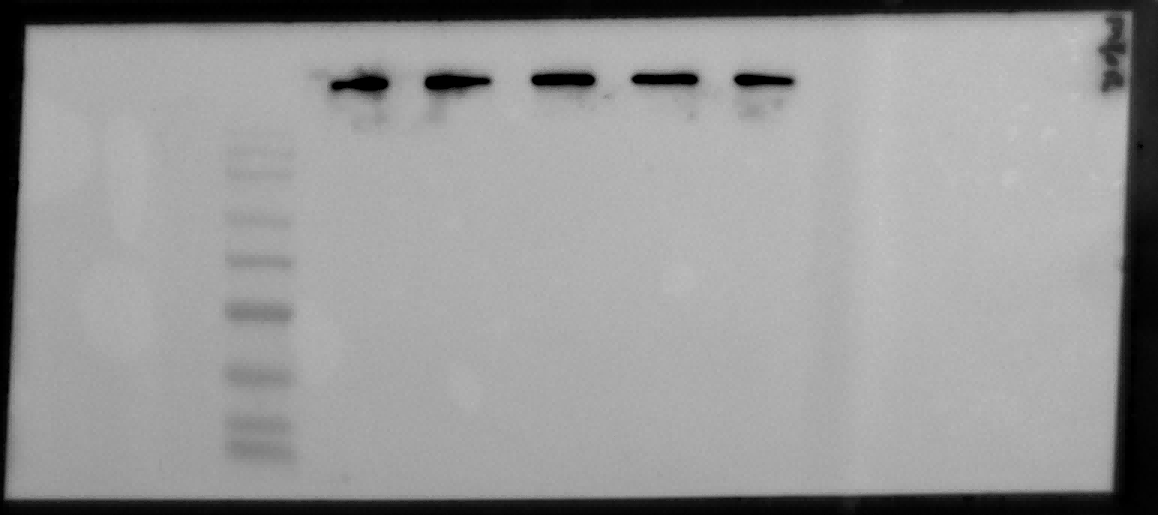

Supplement: Supplementary file 1 [file DataSheet1.zip › Data/Figure.3/Figure/Western Blot/Figure.3 2/MTOR 1.png]

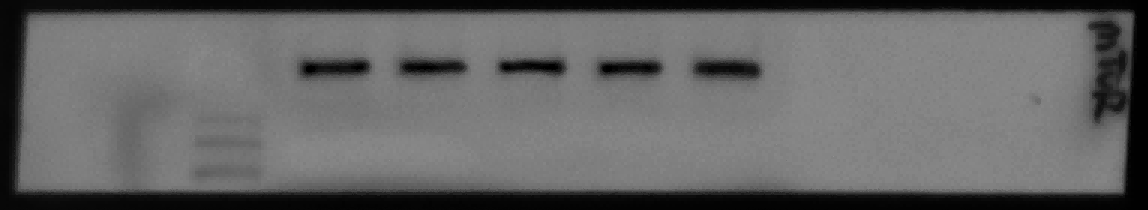

Supplement: Supplementary file 1 [file DataSheet1.zip › Data/Figure.3/Figure/Western Blot/Figure.3 2/MTOR 2.png]

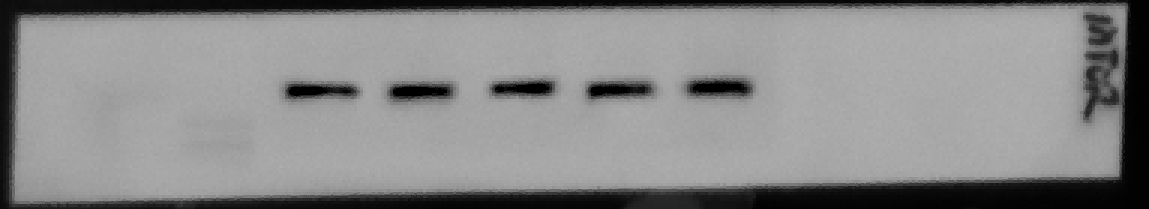

Supplement: Supplementary file 1 [file DataSheet1.zip › Data/Figure.3/Figure/Western Blot/Figure.3 2/MTOR 3.png]
